# Optimizing clinic consultations in primary biliary cholangitis: International consensus recommendations

**DOI:** 10.1097/HC9.0000000000000835

**Published:** 2025-11-12

**Authors:** Heike Bantel, Gideon M. Hirschfield, David E. Jones, Kris V. Kowdley, Ana Lleo, Robert Mitchell-Thain, Ann Moore, Carol Roberts, Atsushi Tanaka

**Affiliations:** 1 Department of Gastroenterology, Hepatology, Infectious Diseases and Endocrinology, Hannover Medical School, Hannover, Germany; 2 The Autoimmune and Rare Liver Programme, Division of Gastroenterology and Hepatology, Toronto General Hospital, Toronto, Ontario, Canada; 3 Translational and Clinical Research Institute, Newcastle University, Newcastle, UK; 4 Department of Clinical research, Liver Institute Northwest, Elson S. Floyd College of Medicine, Washington State University, Seattle, Washington State, USA; 5 Department of Biomedical Sciences, Humanitas University Pieve Emanuele, Milan, Italy; 6 Internal Medicine and Liver Unit, Department of Gastroenterology IRCCS Humanitas Research Hospital, Rozzano, Milan, Italy; 7 PBC Foundation, Edinburgh, UK; 8 Arizona Liver Health, Peoria, Arizona, USA; 9 PBCers Organization, Rochester, New York, USA; 10 Department of Medicine, Teikyo University of Medicine, Tokyo, Japan

**Keywords:** Delphi study, fatigue, primary biliary cholangitis, pruritus, sicca syndrome, symptom assessment

## Abstract

**Background:**

There is variability in primary biliary cholangitis (PBC) care delivery in practice, which can negatively impact disease management and patients’ quality of life. Patient–provider consultations are key to ensure optimal and comprehensive disease management; however, the quality and substance of this interaction may vary. A Delphi study was carried out to provide consensus recommendations to improve and standardize consultations between providers and patients with PBC.

**Methods:**

An international survey of 151 healthcare professionals was conducted with input from 9 PBC experts (6 physicians, 1 patient, 1 patient advocate, and 1 nurse practitioner) who formed the Delphi panel. Informed by findings of the survey, the panel used Delphi methodology to develop best practice recommendations with the aim of informing the broader care framework. Consensus was defined as ≥75% agreement among panel members.

**Results:**

We identified 15 best practice recommendations across 3 principal areas: “patient–provider discussions at diagnosis/first consultation,” “symptom assessment and ongoing management,” and “wider care and support.” These consensus-based recommendations are a resource for providers to help address patient needs around symptom burden, referrals to specialists other than hepatologists, and connection to patient support organizations.

**Conclusion:**

We provide specific recommendations for providers to optimize consultation for patients with PBC with the aim of improving care and patient satisfaction.

## BACKGROUND

Primary biliary cholangitis (PBC) is a chronic, autoimmune cholestatic liver disease. It impacts patients due to both the potential for progression to end-stage liver disease and its associated symptoms, for example, cholestatic pruritus (itch), sicca syndrome, and fatigue, which can profoundly affect their quality of life (QoL).^1^,^2^,^3^,^4^ Current PBC practice recommendations primarily focus on slowing disease progression and less on improving or maintaining health-related QoL through symptom management.^1^,^2^,^4^

As PBC is a rare disease (affecting 15/100,000 people globally), its management faces some of the challenges encountered by other rare diseases.^5^ For example, there is variability in PBC care, partially because most patients with PBC are seen by general gastroenterologists or hepatologists who have limited experience in managing PBC.^6^ To address these challenges, a Delphi study with a panel of clinical and patient experts was conducted to build consensus recommendations for consultations between providers and patients with PBC that could improve care and patient satisfaction.

## METHODS

### Study design and participants

**FIGURE 1:**
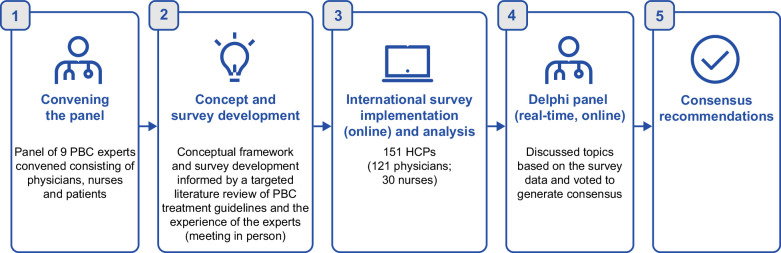
Overview of the study. Abbreviations: HCP, healthcare professional; PBC, primary biliary cholangitis.

The Delphi panel included 9 PBC experts [6 physicians (Canada, Germany, Italy, Japan, UK, and the US), a nurse practitioner (US), and 2 patient experts/advocates (UK and the US)] who were recruited by the study sponsor based on their expertise, ensuring representation from both clinical and patient perspectives in the management of PBC. To understand variability in clinical practice, an international survey of healthcare professionals (HCPs) was first conducted with input from the Delphi panel. This survey informed discussions to establish consensus recommendations on best practice for clinic consultations in PBC. Consensus was reached if ≥75% of the experts agreed. The study was implemented as shown in Figure 1. Detailed methods for the Delphi and survey are reported in Supplemental Material 1, http://links.lww.com/HC9/C158, and Supplemental Material 2, http://links.lww.com/HC9/C159, respectively.

## RESULTS

### Best practice recommendations

**FIGURE 2:**
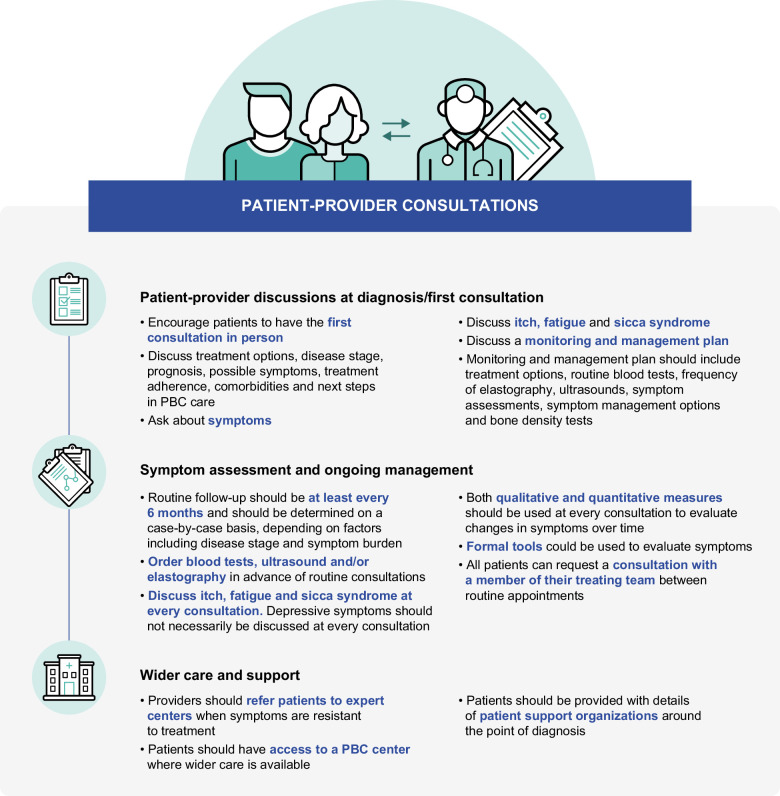
Consensus recommendations for clinic consultations. Abbreviation: PBC, primary biliary cholangitis.

Consensus was reached on 15 best practice recommendations across 3 key areas examined in the survey (Figure 2): “patient–provider discussions at diagnosis/first consultation,” “symptom assessment and ongoing management,” and “wider care and support.”

Additional details of the panel discussions are provided in Supplemental Material 1, http://links.lww.com/HC9/C158.

### Patient–provider discussions at diagnosis/first consultation

While acknowledging the potential role for telemedicine in the care pathway, panelists reached consensus (100% agreed) that patients should be encouraged to have their first consultation in person.

**FIGURE 3:**
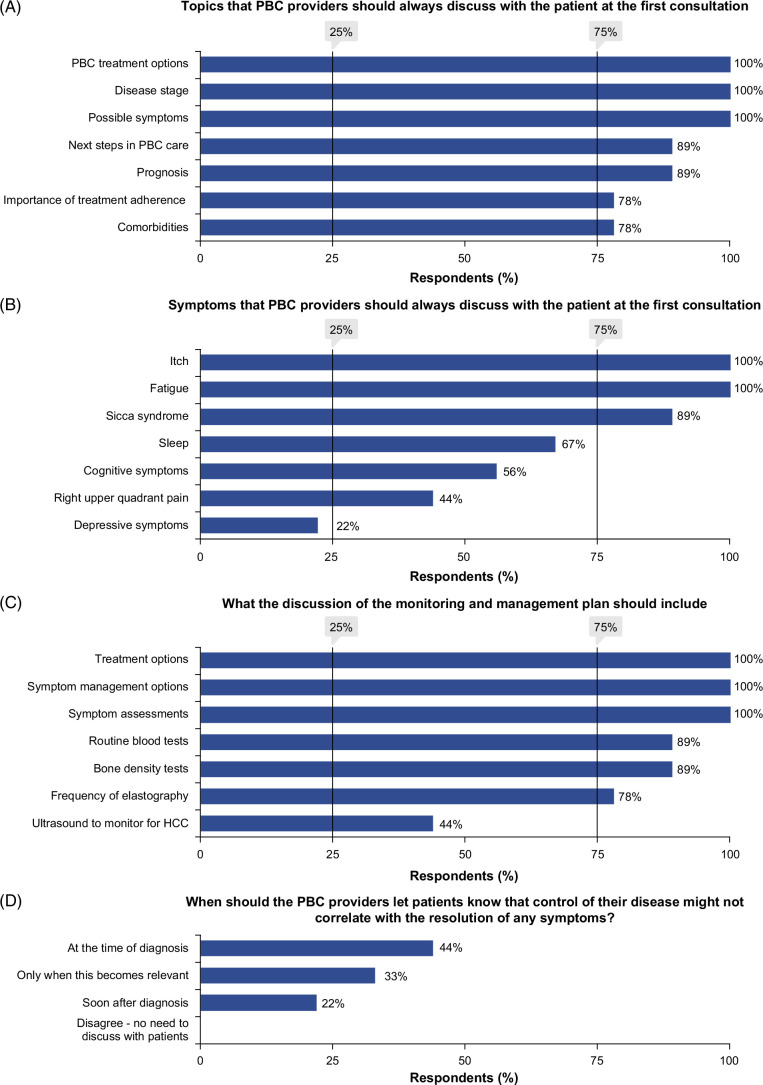
Patient–provider discussions at diagnosis/first consultation: Consensus voting results. Abbreviation: PBC, primary biliary cholangitis.

Consensus was also reached that the following topics should always be discussed at that first consultation (Figure 3A): disease stage (100%), prognosis (89%), treatment options (100%), importance of treatment adherence (78%), possible symptoms (100%), role of comorbidities (78%), and next steps in PBC care (89%). The panelists recognized time constraints as a key factor shaping the consultation topics, emphasizing the importance of prioritization based on clinical urgency, disease stage, and patient preferences. Panelists also discussed signposting patients to support organizations at the first consultation. Only 28% of survey respondents reported doing so, while 70% reported providing or directing patients to educational materials about PBC (Supplemental Material 2, Appendix: Q12, Q19, http://links.lww.com/HC9/C159).

Most surveyed HCPs (95%) reported proactively asking patients about symptoms at the first consultation (Supplemental Material 2, Appendix: Q13, http://links.lww.com/HC9/C159). Itch and fatigue were the most commonly discussed symptoms with patients (80% and 75%, respectively; Supplemental Material 2, Appendix: Q14, http://links.lww.com/HC9/C159). While consensus was reached (100%) that PBC providers should proactively ask about symptoms at first consultation, there was some disagreement regarding which symptoms to prioritize. Following the first round of voting, consensus was reached that itch and fatigue (100% each), as well as sicca syndrome (89%), should always be discussed (Figure 3B). In the second voting round, consensus was reached that depressive symptoms should not be discussed (78%), but if raised, patients should be signposted to the correct care provider.

Consensus was reached (100%) that PBC providers should always discuss a monitoring and management plan with patients during first consultation covering treatment options (100%), routine blood tests (89%), frequency of elastography (78%), symptom assessments (100%), symptom management options (100%), and bone density tests (89%) (Figure 3C). Panelists agreed that second-line therapies should only be discussed when clinically necessary. In the survey, symptom management (85%) was among the top 3 discussion topics covered in the management plan, alongside hepatocellular carcinoma (HCC) screening (89%) and cirrhosis prevention (93%) (Supplemental Material 2, Appendix: Q17, http://links.lww.com/HC9/C159). Consensus was not initially reached that discussions should include ultrasound to monitor HCC, as this is performed only for patients with cirrhosis or a high risk of developing it. Further discussions on liver ultrasounds indicated their utility in diagnosing bile duct obstruction and monitoring disease progression. The panel re-voted on whether liver ultrasound, not specifically for HCC monitoring, should be discussed, and reached consensus (78%). In the survey, the top 3 reported tests were routine blood for disease activity and liver function (89%), liver elastography (76%), and liver ultrasound (56%) (Supplemental Material 2, Appendix: 18a, http://links.lww.com/HC9/C159).

All experts agreed that PBC providers should inform patients that disease control rarely correlates with symptom resolution; but consensus on when to discuss this was not reached [at the point of diagnosis (44%), soon after diagnosis (22%), only when it becomes relevant (33%)] (Figure 3D). The panelists noted that acknowledging the lack of such an association might improve treatment adherence, as some patients believe worsening symptoms indicate treatment failure.

**Table:** 

**Consensus on best practice recommendations: Patient–provider discussions at diagnosis/first consultation**
Patients should be encouraged to have the first consultation in person. Best practice is to discuss disease stage, prognosis, treatment options, treatment adherence, possible symptoms, comorbidities, and next steps in PBC care at the first consultation. PBC providers should proactively ask about symptoms at the first consultation. Itch, fatigue, and sicca syndrome should always be discussed at the first consultation. PBC providers should always discuss a monitoring and management plan with patients during the first consultation. The monitoring and management plan should include treatment options, routine blood tests, frequency of elastography, ultrasounds, symptom assessments, symptom management options, and bone density tests.

### Symptom assessment and ongoing management

**FIGURE 4:**
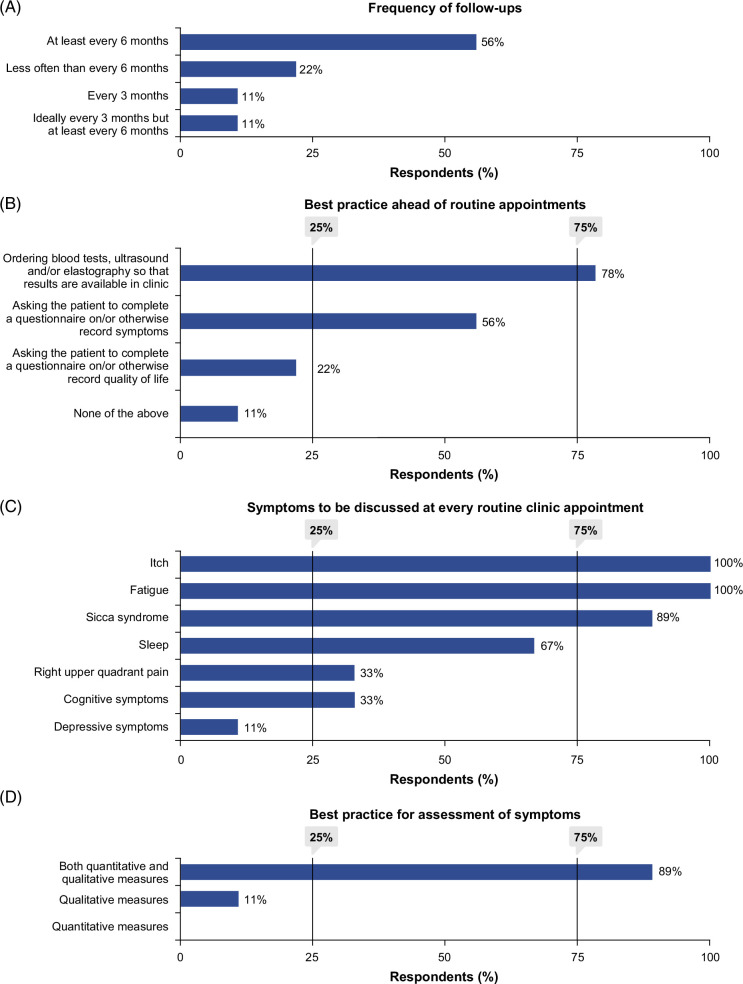
Symptom assessment and ongoing management: Consensus voting results.

Consensus was not reached on an exact frequency for routine follow-up; however, 78% agreed it should be determined on a case-by-case basis and should be at least every 6 months (Figure 4A). The experts considered that the frequency of follow-up should be determined by individual patient factors, including disease stage, symptom burden, and treatment response. Consensus was reached (78%) that the optimal approach would be for blood tests, ultrasound, and/or elastography to be ordered in advance of routine patient–provider consultations (Figure 4B), if the healthcare system renders it feasible, to discuss the results with the patient during the consultation.

In line with discussions regarding the first consultation, panel clinicians acknowledged the need to prioritize which symptoms can be discussed in the consultation time available, with a focus on itch and fatigue, consistent with the survey results (Supplemental Material 2, Appendix: Q24, http://links.lww.com/HC9/C159). They also considered sicca syndrome to be important to discuss at every consultation, though less than a third of surveyed HCPs reported always discussing this. There was, however, acknowledgment from the patient experts that additional symptoms, including sleep interference, should be discussed at every consultation, due to their impact on patients’ lives. Consensus was reached that itch (100%), fatigue (100%), and sicca syndrome (89%) should be discussed at every consultation. As above, consensus was reached (89%) that depressive symptoms should not be discussed at every consultation (Figure 4C).

Most surveyed HCPs (65%) reported leading symptom discussions with patients, while 34% rely solely on patient-initiated conversations (Supplemental Material 2, Appendix: Q22a, http://links.lww.com/HC9/C159). The survey also highlighted variability in symptom assessment, including quantitative measures (eg, scoring symptoms on a scale of 1–10), qualitative methods (ie, listening to and understanding the patient experience), or a combination of both (Supplemental Material 2, Appendix: Q22b, Q22c, http://links.lww.com/HC9/C159). Consensus was reached (89%) that both quantitative and qualitative measures should be used at every consultation to evaluate changes in symptoms over time (Figure 4D). In addition, 78% agreed that formal tools (eg, numerical rating scales, visual analog scales, 5-D itch, and PBC-40) could be used in clinical practice to assess symptoms. Notably, only 54% of survey respondents reported familiarity with formal tools, and fewer than 50% of those reported using them. Reasons for not using these tools included beliefs that other methods better measure symptoms, or perceptions that these are impractical in clinical settings (Supplemental Material 2, Appendix: Q23, Q23a, Q23b, http://links.lww.com/HC9/C159). Panel clinicians noted that qualitative measures are key in capturing how symptoms affect patients’ lives, while quantitative assessments can monitor treatment effects and/or severity of symptoms over time.

Consensus was reached (89%) that the best practice is for all patients to be able to request a consultation with a member of their treating team between routine appointments, especially when there are significant symptom changes, and HCPs have management options. The panel acknowledged that electronic means of contacting providers, including online portals, can be valuable resources for patients, provided they are accessible.

**Table:** 

**Consensus on best practice recommendations: Symptom assessment and ongoing management**
The frequency of routine follow-up should be at least every 6 months and should be determined on a case-by-case basis, depending on factors including disease stage and symptom burden. Best practice is to order blood tests, ultrasound, and/or elastography in advance of routine patient–provider consultations. Itch, fatigue, and sicca syndrome should be discussed at every consultation. Depressive symptoms should not be discussed at every consultation. Both qualitative and quantitative measures should be used at every consultation to evaluate changes in symptoms over time. Formal tools could be used in clinical practice to evaluate symptoms. Best practice is that all patients can request a consultation with a member of their treating team between routine appointments.

### Wider care and support

During discussions, panel clinicians noted the need to refer patients to specialists for specific symptoms when the PBC provider does not have expertise in their management. In contrast, 75% of surveyed HCPs believed they could manage PBC symptoms themselves (Supplemental Material 2, Appendix: Q31c, http://links.lww.com/HC9/C159). Consensus was reached (89%) that PBC providers should refer patients to expert centers when symptoms are resistant to treatment.

While the panelists recognized that access to wider care team members may be limited in some healthcare systems, they reached consensus (78%) that all patients should ideally have access to a PBC center offering wider care. They also acknowledged that there will likely be regional differences in access to wider care, depending on the treatment center.

Panelists stressed that patient support organizations have an important role to play in providing information on topics that providers may not be able to discuss during initial consultations, as well as supporting patients in coping with symptoms in between consultations. Consensus was reached (89%) that all patients should receive information about reliable patient support organizations at diagnosis or subsequent routine appointments, as it is beneficial to signpost patients toward additional support at any stage of care.

**Table:** 

**Consensus on best practice recommendations: Wider care and support**
Providers should refer patients to expert centers when symptoms are resistant to treatment. Patients should have access to a PBC center where wider care is available. All patients should be provided with details of patient support organizations around the point of diagnosis.

## DISCUSSION

This Delphi study identified 15 best practice recommendations that should help providers optimize the time spent with patients with PBC and enhance the patient–provider partnership. These recommendations complement those outlined by the European Reference Network in 2025 regarding key questions patients could ask their physicians to improve their PBC management.^7^ Together, they can establish a standardized baseline for discussions and serve as guidance to enhance the quality of communication, especially for less experienced PBC providers, to facilitate comprehensive care, and improve patient experience and outcomes, including better QoL. Furthermore, they could help overcome and minimize discrepancies in care by enabling more consistent consultations across providers.

The survey underlined a lack of familiarity among HCPs with formal tools/scales for symptom evaluation, consistent with studies showing that symptoms are underreported and undertreated.^3^,^7^,^8^ Panel providers noted that simple 0–10 numerical rating scales constitute a formal tool and should be integrated into routine clinical practice. Apps to assess symptoms are in development and could form part of future symptom evaluation approaches.^9^

This Delphi study provided insight into current PBC clinic consultation practices. Despite the many strengths of this rigorous process, some limitations should be acknowledged. The consensus recommendations focused on 3 key topics that were validated by the panel experts. While additional areas in PBC care may exist, these topics allowed the study to develop feasible recommendations to improve care and support current practice guidelines. The Delphi included experts from 6 countries from North America, Europe, and Asia; however, not all regions were represented. Inclusion of both clinicians and patient representatives in the Delphi panel allowed a patient perspective to inform the best practice recommendations. However, with a 7:2 clinician-to-patient ratio, it was possible to reach consensus without patient agreement. Considering the nature of a Delphi panel (anonymized voting and aggregate responses), responses could not be linked to individuals, though discussions revealed differing perspectives on some topics.

Overall, these recommendations can become a dynamic document that could continue to develop and evolve over time to optimize the patient–provider partnership in PBC. As such, a future step could be to validate these recommendations through an additional Delphi study involving non-PBC specialists or a larger patient group.

## CONCLUSIONS

This international group of PBC experts provided recommendations on which topics to prioritize to support comprehensive consultations between PBC patients and providers, while allowing for an individualized, patient-centric approach. Together with the European Reference Network recommendations about what patients should ask their providers, these provide an outline for how best to use consultations to address patient needs around symptom burden and set a standardized baseline to improve the patient–provider partnership, and hence patient experience, care, and outcomes, including QoL. Ultimately, it would be beneficial for such recommendations to be integrated into practice guidelines to ensure comprehensive patient care.

## Supplementary Material

## Data Availability

Data used for this publication were generated by M3 Global Research. For access to anonymized subject-level data, please contact M3 Global Research. All authors contributed to drafting and critically revising the manuscript. All authors are accountable for the work and have provided their agreement for submission to the journal and approval for publication. This study was funded by GSK (study number: 223362). The funder did not put any restrictions on the recommendations provided.
