# Supplementary material for: Optimizing clinic consultations in primary biliary cholangitis: International consensus recommendations
Source: Hepatol Commun. 2025 Nov 12;9(12):e0835. doi: 10.1097/HC9.0000000000000835 (PMC12614670; doi:10.1097/HC9.0000000000000835)
Supplement: Supplementary file 1 [file hc9-9-e0835-s001.docx]

# Supplementary Material 1

# Content

[Supplementary Material 1 1](#_Toc203119232)

[Content 1](#_Toc203119233)

[Methods (Delphi study) 2](#_Toc203119234)

[1.1 Conceptual framework 2](#_Toc203119235)

[Supplementary Table 1. Conceptual framework 2](#_Toc203119236)

[1.2 Online survey 3](#_Toc203119237)

[1.3 Delphi panel 3](#_Toc203119238)

[Results 4](#_Toc203119239)

[1.1 Patient-provider discussions at diagnosis/first consultation 4](#_Toc203119240)

[1.2 Symptom assessment and ongoing management 6](#_Toc203119241)

[1.3 Wider care and support 7](#_Toc203119242)

[References 9](#_Toc203119243)

#

## Methods (Delphi study)

### 1.1 Conceptual framework

Based on targeted literature review of PBC treatment guidelines and relevant literature, as well as input from the PBC experts, a conceptual framework for the study was developed, which captured 3 key themes and related potential consensus topics that were to be explored in the study with the aim of informing the broader care framework (**Supplementary Table 1**): (1) patient-provider discussions at diagnosis/first consultation: what should be discussed at the first consultation; (2) symptom assessment and ongoing management: which symptoms should be assessed, how symptoms should be assessed in practice, and how frequently; (3) wider care and support: how allied care professionals and patient support organizations could best benefit patients.

### Supplementary Table 1. Conceptual framework

| **Patient-provider discussions at diagnosis/first consultation** | - - What should be included in the discussion at diagnosis/first consultation?     - Should discussions include an explanation of prognosis, risk of transplant, and separation of symptoms and disease stage?   - Should providers proactively discuss symptoms, even if not raised by patients?     - Should a comprehensive list of symptoms be discussed? Should a specific list be discussed, and, if so, which symptoms, and how is this decided?   - Should patients be made aware of symptoms that may arise over the course of the disease?   - How should goals be discussed?     - Should the discussion about treatment objectives incorporate symptom management? |
| --- | --- |
| **Symptom assessment and ongoing management** | - - How frequently should routine consultations take place?   - Which symptoms should be assessed (all, or just selected symptoms)?     - Should providers proactively ask about all symptoms or rely on a patient to self-report any new symptoms?     - Are there any symptoms that should be asked about each time?   - Should formal tools be used to aid symptom identification and management?     - If so, what would these tools look like (e.g., length, qualitative vs quantitative assessment, symptom-specific vs more general) and for which symptoms? If not, how should symptoms be evaluated (e.g., ad hoc conversations)?   - Should the approach to symptom management differ depending on whether PBC is stable or not?   - If a patient develops a symptom (or symptoms worsen) between clinic visits, how could they report this?     - Should patients have an opportunity for an earlier consultation if they develop symptoms? How does this differ by symptom? What factors determine the need for an early consultation? |
| **Wider care and support** | - - What allied HCP resource is available, and what is the optimal role for these HCPs in disease management and patient support?   - Should providers suggest that patients access patient support organizations?     - If so, how and when should this happen?   - Is there any situation where a patient would require referral for symptom management?   - Should symptoms trigger referral to a tertiary center / PBC specialist? Does this differ by symptom? |

HCP, healthcare professional; PBC, primary biliary cholangitis.

### 1.2 Online survey

The conceptual framework, and further input from the panel, helped shape the international survey, which was conducted online (for survey methods, see **Supplementary Material 2, Methods**). Using the results from the survey (**Supplementary Material 2, Appendix**), a series of questions were developed for a real-time virtual Delphi study.

### 1.3 Delphi panel

One Delphi panel meeting was conducted. During the meeting, the results of the online survey were presented to inform panelists’ discussions on current clinical practice in PBC management, with the intent of gaining consensus on key issues. The panel then had the opportunity to vote anonymously on draft consensus statements via an online platform (Vevox, Auga Technologies Ltd). Responses to each question were anonymous to both the panel attendees and the moderators and were not attributable to individual participants, unless a participant willingly disclosed their votes. Consensus was reached if at least 75% (i.e., 7 out of 9) of the experts were in agreement. If no consensus was reached following the first round of responses, a discussion took place to further explore opinions. Voting was then repeated for a second round, and if the panelists were still not in agreement, consensus was not reached. For any panelists unable to attend on the day, their votes were collected in an offline manner and combined with the rest of the votes to determine consensus. The Delphi panel meeting was conducted on October 16^th^, 2024; however, it took an additional week to reach consensus on all statements due to the reasons outlined above.

Given that it was possible to gain consensus by ascertaining agreement among at least 7 out of 9 panelists, consensus was already gained during the panel meeting for some statements; however, for others, confirmation of consensus came following offline receipt of the final panel members’ votes. Therefore, the final results were shared with all participants a week following the panel meeting.

## Results

Further details of the Delphi panel discussions are provided below for additional context to the consensus recommendations reached.

### 1.1 Patient-provider discussions at diagnosis/first consultation

**Recommendation 1: Patients should be encouraged to have the first consultation in person**

Advantages of face-to-face first interaction, aligned with patient preference, include building a relationship with the patient, sensing how comfortable they are and what questions they have, being able to alleviate their concerns and provide reassurance. Panelists acknowledged differences in individual patient choice, as well as potential cultural or geographic differences, and agreed that virtual consultations should be offered when appropriate.

**Recommendation 2: Best practice is to discuss disease stage, prognosis, treatment options, treatment adherence, possible symptoms, comorbidities, and next steps in PBC care at the first consultation**

Panelists noted that trusted patient support organizations should be discussed (for example the PBC Foundation, PBCers Organization, and American Liver Foundation),^1-3^ as they can provide much of the relevant disease background information that patients are likely to want to access and direct patients to local support groups. Providers are also encouraged to explore the latest available resources on the websites of the American Association for the Study of Liver Diseases (AASLD), European Association for the Study of the Liver (EASL), and the Asian Pacific Association for the Study of the Liver (APASL).^4-6^

**Recommendation 3 & 4: PBC providers should proactively ask about symptoms at the first consultation & itch, fatigue, and sicca syndrome should always be discussed at the first consultation**

There was a recognition that from a patient perspective, different symptoms could be considered important. For example, a patient may be experiencing fatigue but would not necessarily be able to link this to affected cognition. Moreover, patients may also be unaware that certain cognitive symptoms are linked to their PBC, even if their daily life is being impacted. Therefore, some panel experts considered that cognitive symptoms should always be discussed in practice, in addition to sleep and fatigue, while acknowledging that the approach to the discussions will be individual to each patient. From a patient’s perspective, discussion of potential depressive symptoms at first consultation could facilitate validating patients’ feelings around the psychological impact of diagnosis, including anxiety, which is experienced by many symptomatic patients due to delayed time to diagnosis. Furthermore, the importance of interrogating sleep, cognitive symptoms, and right upper quadrant pain was also discussed, but no consensus was reached about whether these symptoms should or should not be addressed at the first visit.

**Recommendation 5: PBC providers should always discuss a monitoring and management plan with patients during the first consultation**

Consensus was not reached regarding the timepoint at which PBC clinicians should discuss the risk/likelihood of needing a liver transplant. Some clinicians noted they rarely discuss liver transplant at the first consultation because the risk of needing a liver transplant is low in most patients with PBC, and raising it proactively may increase the patient’s levels of concern. Other clinician panel members disagreed and noted that they proactively mention the low likelihood of liver transplant at the first consultation to reassure patients or dispel out-of-context information that a patient might read online about the risk of needing a liver transplant. Patient representatives on the panel noted that patients may be concerned about different aspects of their disease, particularly fears of liver failure and the potential need for a transplant. Reassuring patients that they will be able to live as normal a life as possible should be a key priority during the first consultation. Although the panel did not come to an agreement as to whether the low likelihood of requiring a liver transplant should always be raised at first consultation, there was agreement that this is an important topic for future consultations to reassure patients and dispel out-of-context information a patient may have encountered.

Overall, the first consultation was recognized as the start of a joint patient-provider journey, requiring patient-centric consultations and discussions tailored to the individual patient’s disease stage, prior treatment experience, and preferences for information.

### 1.2 Symptom assessment and ongoing management

Practice guidance/guidelines do not specify how or when to evaluate symptoms. The panel of experts explored these topics and identified areas for further exploration.

**Recommendation 2: Best practice is to order blood tests, ultrasound, and/or elastography in advance of routine patient-provider consultations**

The panel did not reach consensus on whether best practice should include asking patients to complete questionnaires on symptoms and/or QoL ahead of time, as many organizations lack the systems necessary to provide or record these. However, it was noted that, where possible, PBC providers could attempt to utilize patient time in the waiting room ahead of consultations.

**Recommendation 3: Itch, fatigue, and sicca syndrome should be discussed at every consultation. Depressive symptoms should not be discussed at every consultation**

The panel discussions highlighted some differences of opinion between clinician and patient representatives with regards to discussing sleep disturbances. From a patient’s perspective, sleep was highlighted as an important symptom that should be discussed in every consultation alongside itch, fatigue, and sicca syndrome. However, while nearly all panel members agreed that sleep should be addressed at every routine clinic visit (6 out of 9 respondents), some clinicians expressed concerns that this may not be feasible due to limited consultation times. It was suggested that providers could inquire about sleep if patients report suffering from other symptoms. Ultimately, consensus was not reached on whether sleep should or should not be discussed in every routine consultation. Furthermore, the panel did not reach consensus on whether right upper quadrant pain or cognitive symptoms should or should not be discussed at every routine consultation and highlighted that PBC providers may not have the expertise to manage cognitive symptoms.

**Recommendation 4: Both qualitative and quantitative measures should be used at every consultation to evaluate changes in symptoms over time**

The survey highlighted some variation in how symptoms are assessed, i.e. using quantitative measures, qualitative measures, or a mix of both. Few HCPs surveyed solely used quantitative measures to either evaluate symptoms or changes over time (13% and 10%, respectively; **Supplementary Material 2, Appendix: Q22b, Q22c**); instead, qualitative measures (45% and 36%, respectively) or a mix of qualitative and quantitative measures (42% and 50%, respectively) were more often used.

**Recommendation 6: Best practice is that all patients can request a consultation with a member of their treating team between routine appointments**

The survey asked HCPs about available communication options for patients experiencing changes in symptoms between scheduled consultations. The majority (75%) responded that patients can request a consultation with a physician (**Supplementary Material 2, Appendix: Q27**). However, a substantial minority (25%) reported that patients under their care would have to either contact their general practitioner or wait several months until their next routine appointment to discuss these changes.

### 1.3 Wider care and support

**Recommendation 1: Providers should refer patients to expert centers when symptoms are resistant to treatment**

The consensus-based recommendation that PBC providers should refer patients to expert centers when symptoms are resistant to treatment is in contrast to the HCP survey results, where many HCPs believed they are able to manage PBC symptoms themselves (**Supplementary Material 2, Appendix: Q31c**). This potentially highlights a knowledge gap, where some PBC providers do not fully understand the broad range of possible PBC symptoms may include those that lie outside their area of expertise.

**Recommendation 3: All patients should be provided with details of patient support organizations around the point of diagnosis**

The importance of patient support organizations in providing information on topics that providers may not be able to discuss at diagnosis due to time constraints, as well as helping patients cope with symptoms between consultations, was highlighted in the discussions. Many support organizations have medical specialists advising them and can provide information that providers are unable to due to time constraints. However, the survey results showed that many HCPs (42%) are not discussing support organizations with patients (**Supplementary Material 2, Appendix: Q30**). One clinician noted that there was likely an awareness gap regarding patient support organizations, where providers who are less familiar with PBC may be less aware that local or national organizations exist, and are therefore not providing details to patients, a view that was at least partly supported by the survey data (**Supplementary Material 2, Appendix: Q30c**). Overall, the key role of patient support organizations in transforming the patient journey was acknowledged during discussions.

Given the chronic nature of PBC, care should be seen as an ongoing partnership between providers and patients, and the consultations should be personalized to the individual needs of each patient. However, what the patients discuss with the physician and what they discuss with other HCPs involved in the day-to-day management of their PBC may vary. Furthermore, the majority of PBC-related care may rest with different roles in different settings.

Overall, these recommendations could help overcome and minimize discrepancies in care across care providers by providing an opportunity for more uniform consultations, thereby improving the patient’s experience.

## References

1. PBC Foundation. https://www.pbcfoundation.org.uk/. Accessed March 3, 2025.

2. PBCers Organization. https://pbcers.org/. Accessed March 5, 2025.

3. American Liver Foundation. https://liverfoundation.org/. Accessed March 14, 2025.

4. American Association for the Study of Liver Diseases (AASLD). https://www.aasld.org/. Accessed March 6, 2025.

5. European Association for the Study of the Liver (EASL). https://easl.eu/. Accessed March 6, 2025.

6. Asian Pacific Association for the Study of the Liver (APASL). https://www.apasl.info/. Accessed March 14, 2025.
