# Supplementary material for: Optimizing clinic consultations in primary biliary cholangitis: International consensus recommendations
Source: Hepatol Commun. 2025 Nov 12;9(12):e0835. doi: 10.1097/HC9.0000000000000835 (PMC12614670; doi:10.1097/HC9.0000000000000835)
Supplement: Supplementary file 2 [file hc9-9-e0835-s002.docx]

# Supplementary Material 2

# Content

[Supplementary Material 2 1](#_Toc199933359)

[Content 1](#_Toc199933360)

[Methods (Survey) 4](#_Toc199933361)

[Supplementary Table 1. Survey inclusion criteria 5](#_Toc199933362)

[Supplementary Table 2. Planned survey participant recruitment 6](#_Toc199933363)

[Statistical methods 6](#_Toc199933364)

[Subgroup analyses 7](#_Toc199933365)

[Ethics approval and consent to participate 7](#_Toc199933366)

[Data management 7](#_Toc199933367)

[Changes in the conduct of the study 8](#_Toc199933368)

[Appendix - Survey results 9](#_Toc199933369)

[Role and experience 9](#_Toc199933370)

[Q1. In which country do you currently work? 9](#_Toc199933371)

[Q2. How many years’ experience do you have in managing PBC? 9](#_Toc199933372)

[Q3. Which of the options below best describes your role? 10](#_Toc199933373)

[Q4. Which clinical setting best describes where you primarily see patients with PBC? 10](#_Toc199933374)

[Q5. Typically, how many patients with PBC do you see within 12 months? 11](#_Toc199933375)

[Patient-treater discussions at diagnosis 11](#_Toc199933376)

[Q6. What proportion of first consultations take place virtually or via telephone as opposed to face-to-face? 11](#_Toc199933377)

[Q7. Are you involved in confirming or making a diagnosis of PBC? (nurses only) 12](#_Toc199933378)

[Q8. What proportion of patients already have a diagnosis of PBC when you first see them? 12](#_Toc199933379)

[Q8a. For patients who already have a diagnosis of PBC, where did this diagnosis typically happen? 13](#_Toc199933380)

[Q9. Which tests or assessments do you require to be confident of a diagnosis of PBC? 13](#_Toc199933381)

[Q10. For those patients who already have a diagnosis of PBC, do you typically require any tests to confirm the diagnosis of PBC? 15](#_Toc199933382)

[Q11. What do you consider to be the primary driver(s) of prognosis for PBC? 16](#_Toc199933383)

[Q12. Which of the following do you discuss with patients once you have established their diagnosis of PBC (at the first consultation with them)? 18](#_Toc199933384)

[Q13. At the first consultation with patients when you established their diagnosis of PBC, do you proactively ask patients about whether or not they have symptoms? 22](#_Toc199933385)

[Q14. At the first consultation with patients when you established their diagnosis of PBC, which symptoms do you discuss with patients? 23](#_Toc199933386)

[Q15. Do you discuss that symptoms may appear or change over the course of the disease? 27](#_Toc199933387)

[Q16. When discussing symptoms, do you make patients aware that the control of their disease (in response to treatment) might not correlate with the resolution of any symptoms which may be present? 27](#_Toc199933388)

[Q17. When discussing a PBC management plan with patients, which of the following do you include? 28](#_Toc199933389)

[Q18. Do you discuss a monitoring plan with patients? 29](#_Toc199933390)

[Q18a. Do you discuss a monitoring plan with patients? If yes, which of the following potential tests do you discuss? 29](#_Toc199933391)

[Q19. During this first interaction, do you provide or direct patients to educational materials about PBC? 30](#_Toc199933392)

[Q19a. During this first interaction, do you provide or direct patients to educational materials about PBC? If yes, what do the materials cover? 30](#_Toc199933393)

[Q19b. During this first interaction, do you provide or direct patients to educational materials about PBC? If no, why not? 31](#_Toc199933394)

[Symptom assessment and ongoing management 32](#_Toc199933395)

[Q20. What would you consider routine follow-up for patients with PBC (acknowledging that this may vary depending on disease-specific factors such as treatment response, disease course and stage)? 32](#_Toc199933396)

[Q21. In advance of a routine patient consultation, what action do you take? 32](#_Toc199933397)

[Q22. Do you evaluate symptoms during routine consultations with patients? 33](#_Toc199933398)

[Q22a. Do you evaluate symptoms during routine consultations with patients? If yes, how do you evaluate symptoms? 33](#_Toc199933399)

[Q22b. If you evaluate symptoms during routine consultations with patients, how do you evaluate symptom severity? 34](#_Toc199933400)

[Q22c. If you do evaluate symptoms during consultations, how do you assess changes over time? 34](#_Toc199933401)

[Q23. Are you familiar with any formal tools or scales to measure symptoms and / or the impact of PBC on patients? 35](#_Toc199933402)

[Q23a. Are you familiar with any formal tools or scales to measure symptoms and / or the impact of PBC on patients? If so, which tools? 36](#_Toc199933403)

[Q23b. What are the reasons for not using a tool that you are familiar with in the clinic? 40](#_Toc199933404)

[Q24. For the patients with PBC that you manage, how often do you discuss these specific symptoms with the patient? 41](#_Toc199933405)

[Q25. When you identify a patient with symptoms, how do you prioritize which ones you should proactively manage? 45](#_Toc199933406)

[Q26. Does your approach to symptom management for a patient differ depending on the stage of PBC? 45](#_Toc199933407)

[Q27. If a patient had a change in symptoms between scheduled consultations, which of the following options are available to them? 46](#_Toc199933408)

[Q28. Would quality improvement (QI) metrics that are linked to the management of PBC be useful in your practice? 46](#_Toc199933409)

[Q28a. Would quality improvement (QI) metrics that are linked to the management of PBC be useful in your practice? If yes, please specify which aspects they should cover 47](#_Toc199933410)

[Care and support providers 48](#_Toc199933411)

[Q29. Which wider care team members are available for your patients with PBC? 48](#_Toc199933412)

[Q30. Do you routinely suggest that patients should connect with Patient Organizations? 52](#_Toc199933413)

[Q30a. Do you routinely suggest that patients should connect with Patient Organizations? If so, how is this done? 52](#_Toc199933414)

[Q30b. Do you routinely suggest that patients should connect with Patient Organizations? If so, when is this done? 53](#_Toc199933415)

[Q30c. Do you routinely suggest that patients should connect with Patient Organizations? If not, why not? 53](#_Toc199933416)

[Q31. Do you ever refer a patient to a separate specialist for symptom management? 54](#_Toc199933417)

[Q31a. Do you ever refer a patient to a separate specialist for symptom management? If so, what determines this? 54](#_Toc199933418)

[Q31b. Do you ever refer a patient to a separate specialist for symptom management? If yes, which are the symptoms where you would most commonly refer to a specialist? 55](#_Toc199933419)

[Q31c. Do you ever refer a patient to a separate specialist for symptom management? If no, why not? 55](#_Toc199933420)

[Q33. Who do you consider is responsible for managing extrahepatic complications of PBC (thyroid, bone density, oral health)? 56](#_Toc199933421)

[Reference 56](#_Toc199933422)

#

# Methods (Survey)

The targeted review of clinical guidelines and relevant literature was used to help shape consensus framework and inform the questions for the online survey. Findings from the review were developed into a conceptual framework to ensure that the concepts which were addressed in the survey would inform the consensus discussions in the Delphi element of the study. The conceptual framework was refined following input from the scientific steering committee, where the key topics relating to the comprehensive management of PBC were identified. Based on the conceptual framework, and further input from the scientific steering committee, a survey questionnaire was developed, which was programmed and implemented online.

The survey collected data from healthcare practitioners (HCPs, both physicians and nurses) with experience of treating PBC and provided an understanding of the current clinical practice with regard to diagnosis, symptom screening, and determining treatment response. Survey inclusion criteria were developed in order to ensure that HCPs that were currently involved in managing PBC were recruited (**Supplementary Table 1**). In addition, to ensure that HCPs with a range of familiarity with PBC were recruited, an approximate quota based on caseload formed part of the study criteria. Before participating in the online survey, all participants were required to complete a screening survey to determine their eligibility based on the survey criteria.

## Supplementary Table 1. Survey inclusion criteria

| **Nurses** | **Physicians** |
| --- | --- |
| In role (or clinical practice) for at least 5 years | In role (or clinical practice) for at least 5 years |
| Currently or recently (in the past 6 months) involved in consultations with patients with PBC | Personally responsible for treating patients with PBC, including prescribing drug treatments  Treated at least 2 patients in the last year |
| Treated at least 10 patients in the last year or work in a specialist autoimmune clinic | **Experience / familiarity**  50% treated 10 patients or fewer in prior  12 months; 50% treated more than 10 patients  in past 12 months |
| **Exclusion criteria**  Have authored any national or international guidelines relating to the management of PBC | **Exclusion criteria**  Have authored any national or international guidelines relating to the management of PBC |

PBC, primary biliary cholangitis.

In total, 120 HCPs were planned to be recruited, with specific quotas for role (nurses or physicians), country of practice and experience / familiarity with PBC (**Supplementary Table 1** and **2**). Prospective participants were identified from a database of HCPs, when a potential participant’s profile matched the study inclusion criteria. Participants were then contacted via email and asked to complete a screener questionnaire if they were interested in participating in the survey. The screener questionnaire confirmed if a potential participant met the criteria and did not exceed any country, role or experience quota. Upon meeting the criteria, participants were sent a link to the online survey via email. Recruitment continued until the target number of participants by role and experience in managing PBC was reached.

## Supplementary Table 2. Planned survey participant recruitment

| **Country (participants, n)** | **Participants in the online survey** |
| --- | --- |
| United States (n=55) | 40 Physicians  15 Nurses |
| Germany (n=20) | 15 Physicians  5 Nurses |
| Japan (n=20) | 20 Physicians |
| China (n=20) | 20 Physicians |
| United Kingdom (n=10) | 5 Physicians  5 Nurses |
| Canada (n=10) | 5 Physicians  5 Nurses |
| Italy (n=5) | 5 Physicians |
| Spain (n=5) | 5 Physicians |
| France (n=5) | 5 Physicians |

Two survey questionnaires were developed, one for physicians and one for nurses. The questionnaire development followed an established methodology that included question-set development, validity checking, and questionnaire piloting.^1^ Survey questions were both qualitative and quantitative and responses were categorical, continuous data, or qualitative responses as appropriate. The draft survey content was reviewed by the scientific steering committee to ensure that the questions adequately probed the conceptual framework.

The survey was implemented with support from a third-party vendor (M3 Global Research). Questions were initially developed in English for all markets, and the final surveys were translated into local languages for certain markets (Germany, Japan, and China). They were translated by subject-specialist native-speaker translators, proofread by a second qualified native-speaker translator (to ensure that the content had not changed during the translation) and quality assessed by a third-party linguist.

The results from the survey were analyzed (both qualitatively and quantitatively) following a pre-agreed analysis plan. Only fully completed surveys were included in the analyses.

## Statistical methods

For the analysis of the survey data, quantitative data were summarized using measures of central tendency including mean, median, mode, the level of dispersion (i.e., ranges). Categorical data were summarized in terms of number and percentage of respondents. Questions with numerical responses (continuous variables) were summarized mainly using descriptive statistics (e.g., mean, median, range, standard deviation) in order to understand the distribution of results. These summary statistics were calculated from the aggregated responses of all participants for each country in the sample. For categorical questions with pre-defined answers (a single option or multiple options), including Likert scales, the number and percentage of respondents were calculated for each category. Open-ended questions were recorded as free text. They were analyzed to identify recurring themes and to provide context for the quantitative data collected. Where appropriate, the themes will be treated as categorical data and the number and percentage of respondents in each category will be summarized.

## Subgroup analyses

The results from the online survey were analyzed separately for all participants as well as by pre-specified subgroups: analysed by the type of participants (physicians and nurses), region of interest (North America [US and Canada]; Asia [Japan and China]; Europe [France, Germany, Italy, Spain, and UK]), and level of expertise (caseload).

## Ethics approval and consent to participate

The study protocol was approved through Institutional Review Board, Ethics Review Committee (IRB / IEC) prior to commencing recruitment. Salus IRB and Veritas IRB provided ethical review for the US and Canada, respectively. It was determined that this study was not subject for IRB review in all other markets. Informed consent was obtained from the survey participants before recruitment. Participants could withdraw from the study at any time and for any reason. Before a participant was enrolled in the study, the aims, methods, anticipated benefits, and potential hazards of the study were explained.

## Data management

The survey data was collected through Forsta Surveys (<https://www.forsta.com/resources/forsta-surveys>), which is a cloud-based application hosted by Rackspace, Microsoft Azure, and Amazon Web Services, and is Health Insurance Portability and Accountability Act and General Data Protection Regulation compliant. The survey itself did not collect any sensitive personal data. Survey data was de-identified through assignment of an identifier per participant by the third-party vendor before sending to the research team and research team. The identifier shared with the study team did not include identifiable information, only the third-party vendor was able to link survey responses back to individual participants. Neither the research team, nor the Sponsor was able to link responses back to individual participants.

Participants were sent an email link to the survey from the third-party vendor. The survey was programmed in such a way as to require respondents to answer each question before proceeding to the next which should avoid part completion of responses.

## Changes in the conduct of the study

There were several changes made to the conduct of the study, from those specified in the original protocol. A second IRB was consulted (Veritas) after it was determined that the original IRB was unable to provide an ethical review for Canada. No Canadian survey participants were recruited until ethical approval from Veritas IRB was granted.

Due to slow recruitment of survey participants with low expertise / familiarity in managing PBC, the approximate criteria was changed from treating 5 or fewer patients in the past
12 months to 10 or fewer. The overall target of 50% of physicians in each expertise / familiarity category was maintained.

The third-party vendor erroneously recruited one additional participant from Spain, resulting in six, as opposed to five participants being recruited in this country. As these participant’s answers passed the quality assurance stage, their responses were included in the data analysis. Therefore, 151 HCPs were included in the survey (30 nurses and 121 physicians).

# Appendix - Survey results

## Role and experience

### Q1. In which country do you currently work?


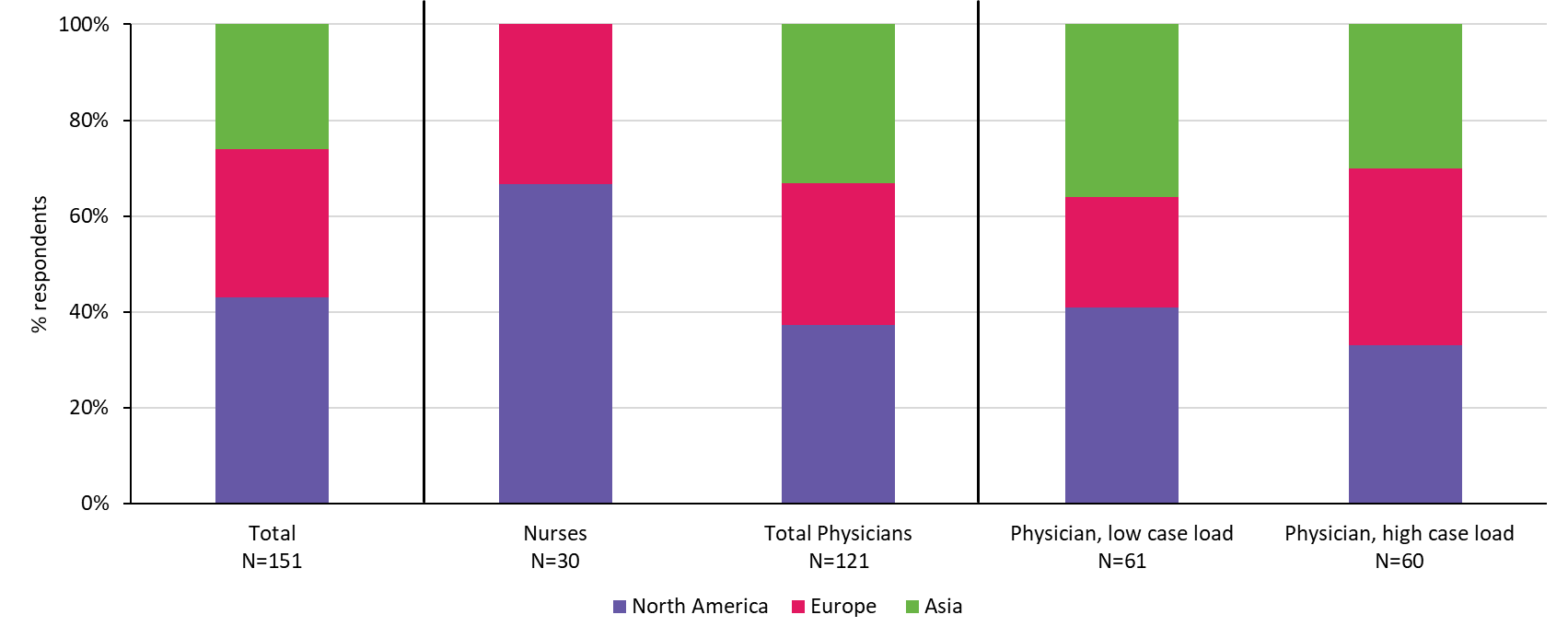


### Q2. How many years’ experience do you have in managing PBC?


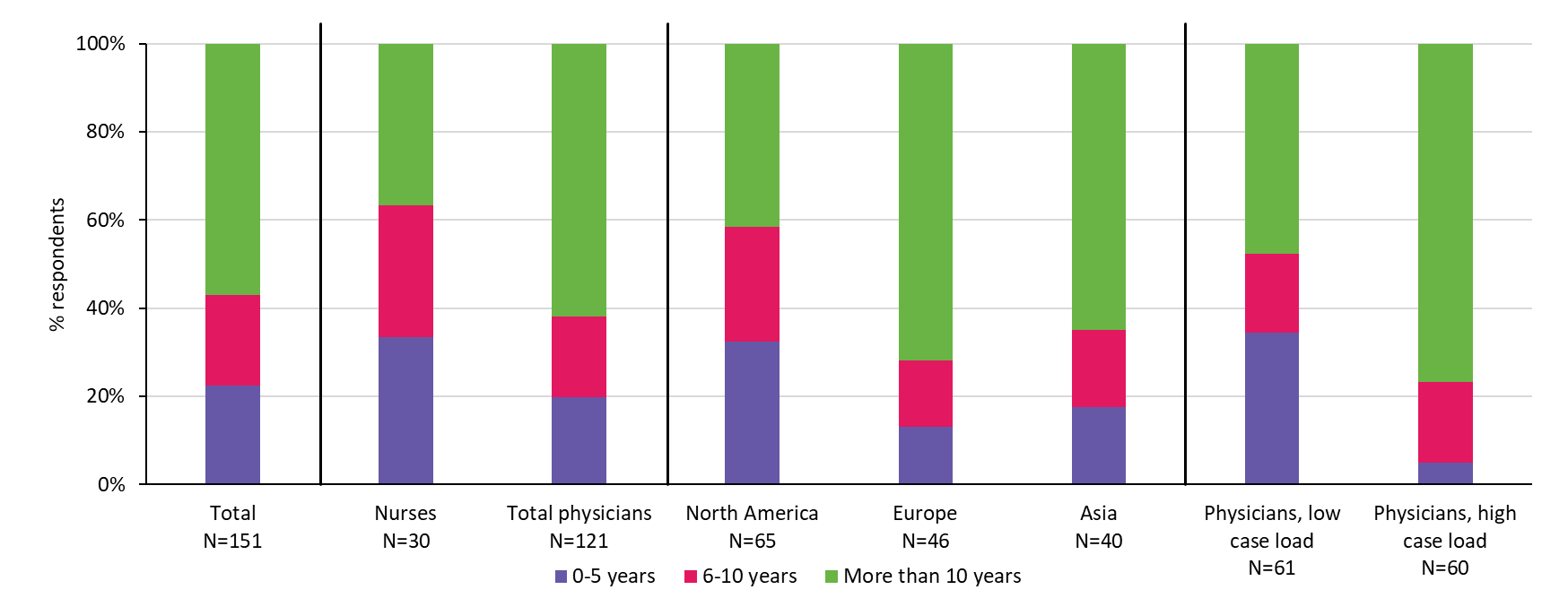


### Q3. Which of the options below best describes your role?


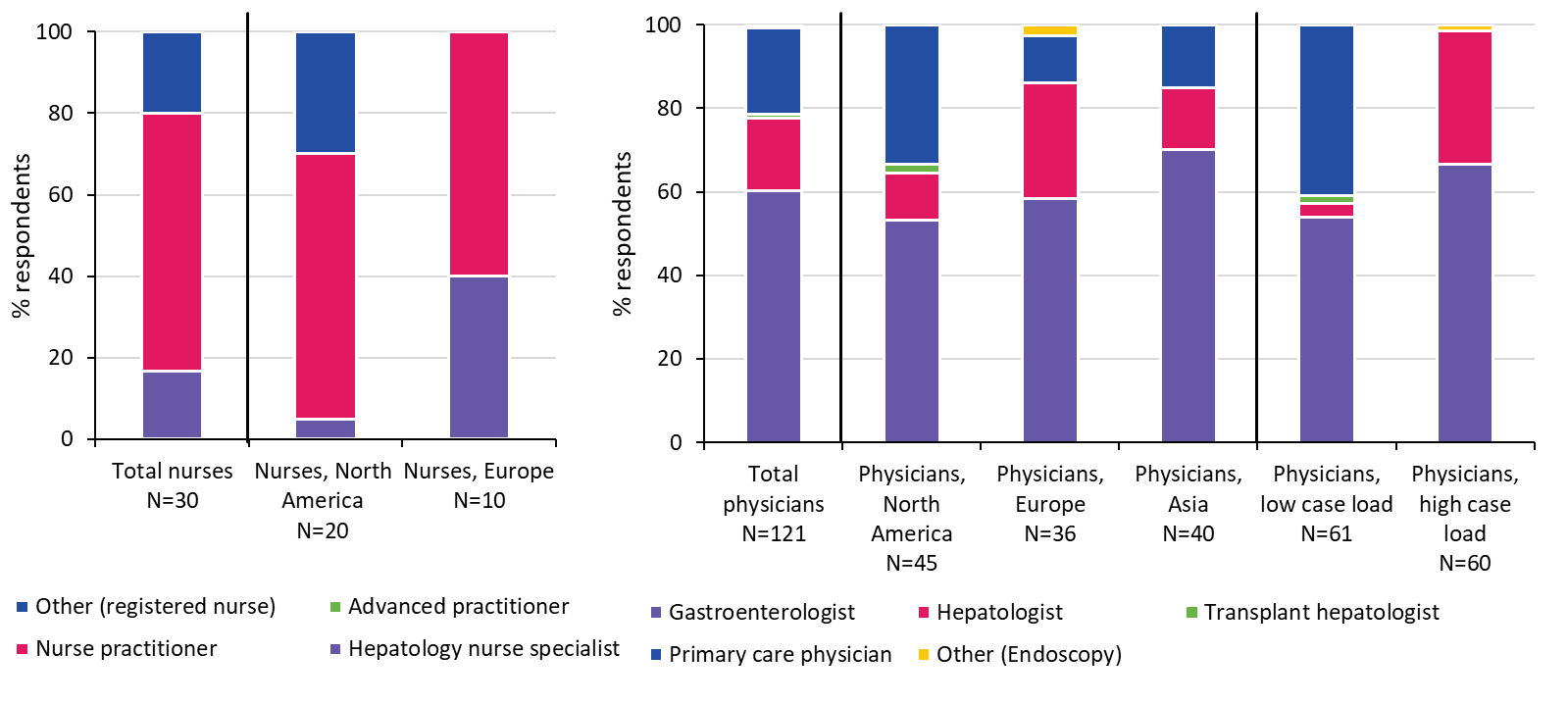


### Q4. Which clinical setting best describes where you primarily see patients with PBC?


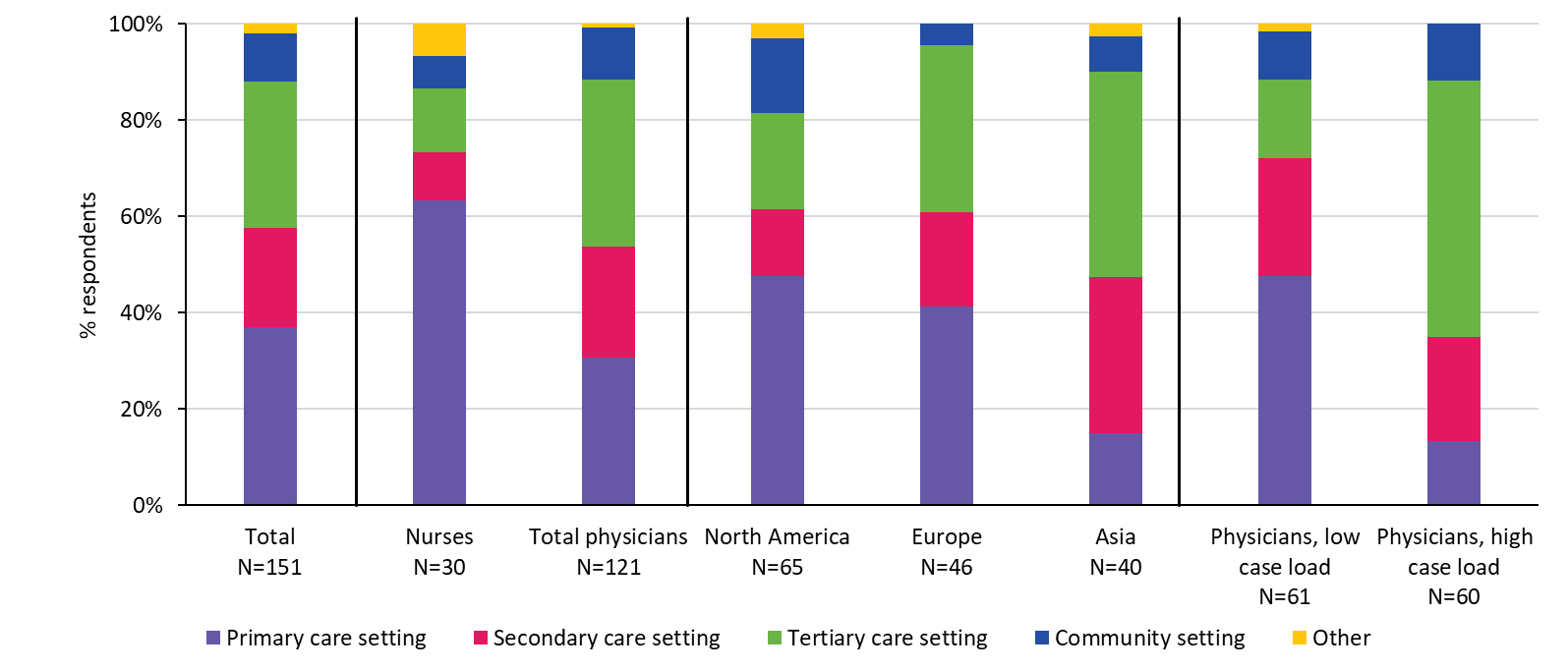


Other responses: both primary and tertiary settings (N=1); geriatric health setting (N=1); inpatient hospital (N=1).

### Q5. Typically, how many patients with PBC do you see within 12 months?


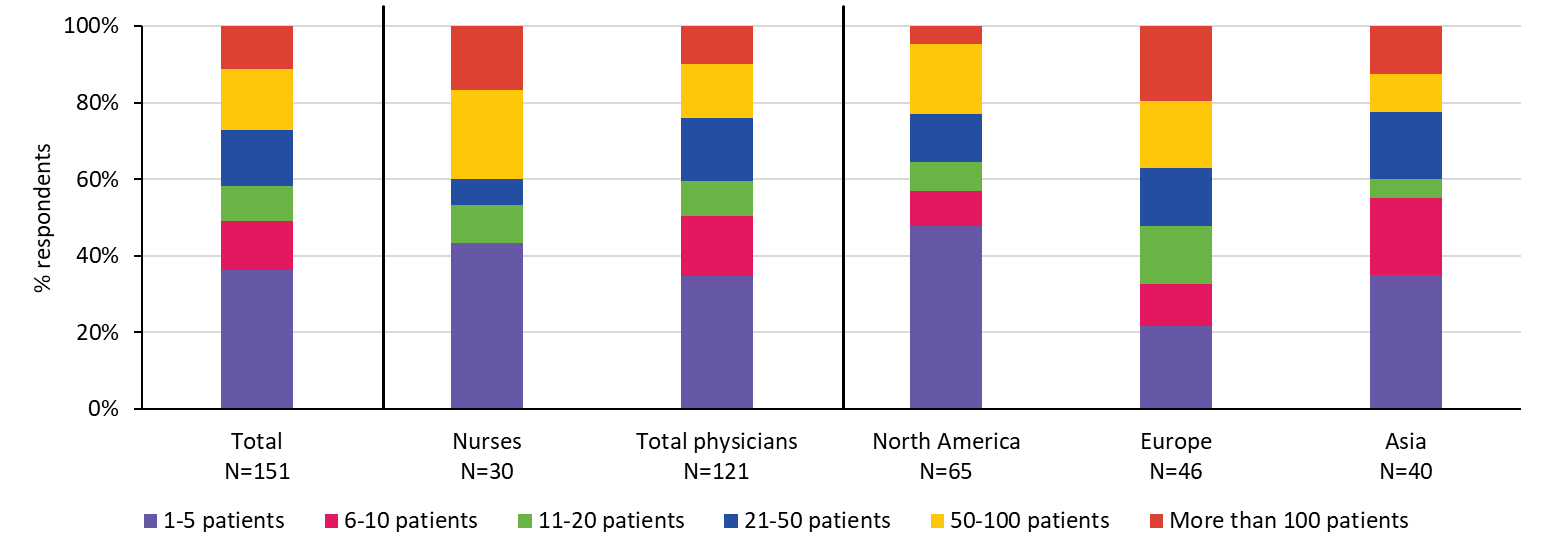


## Patient-treater discussions at diagnosis

### Q6. What proportion of first consultations take place virtually or via telephone as opposed to face-to-face?


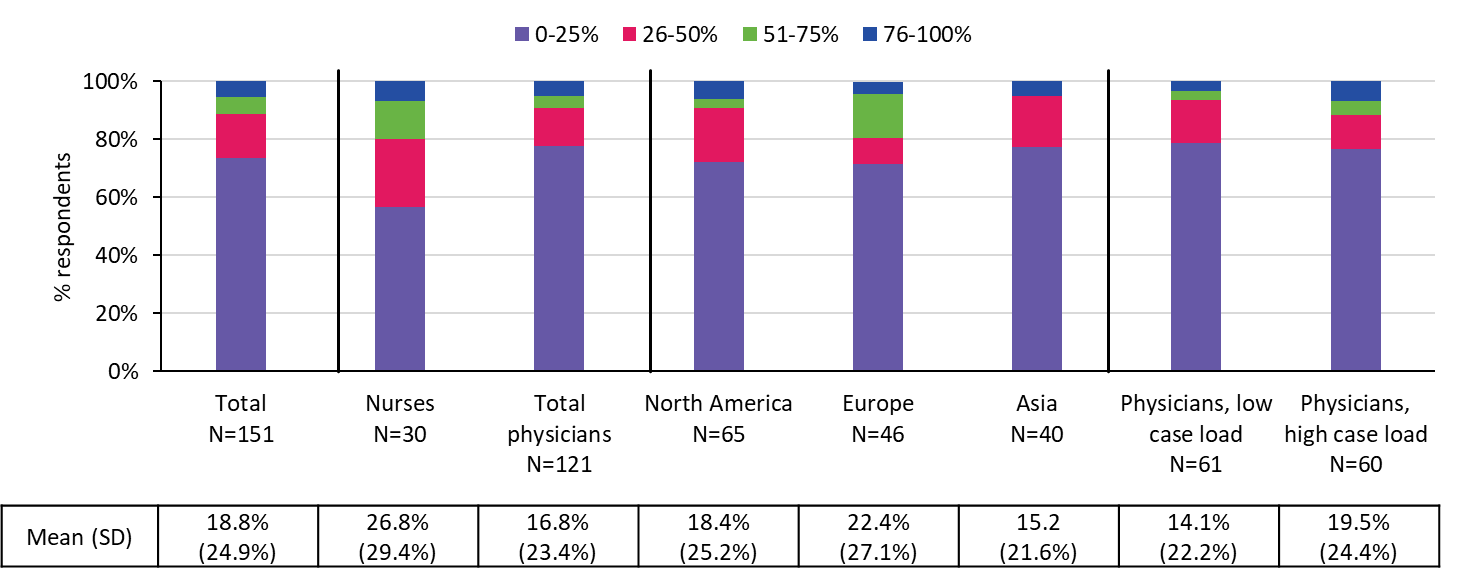


### Q7. Are you involved in confirming or making a diagnosis of PBC? (nurses only)


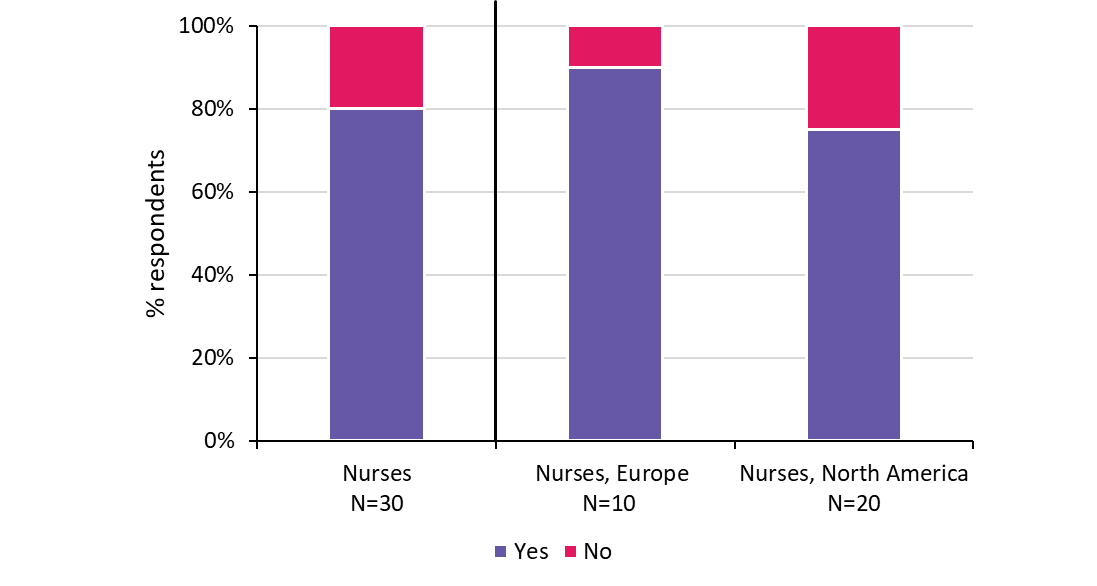


### Q8. What proportion of patients already have a diagnosis of PBC when you first see them?


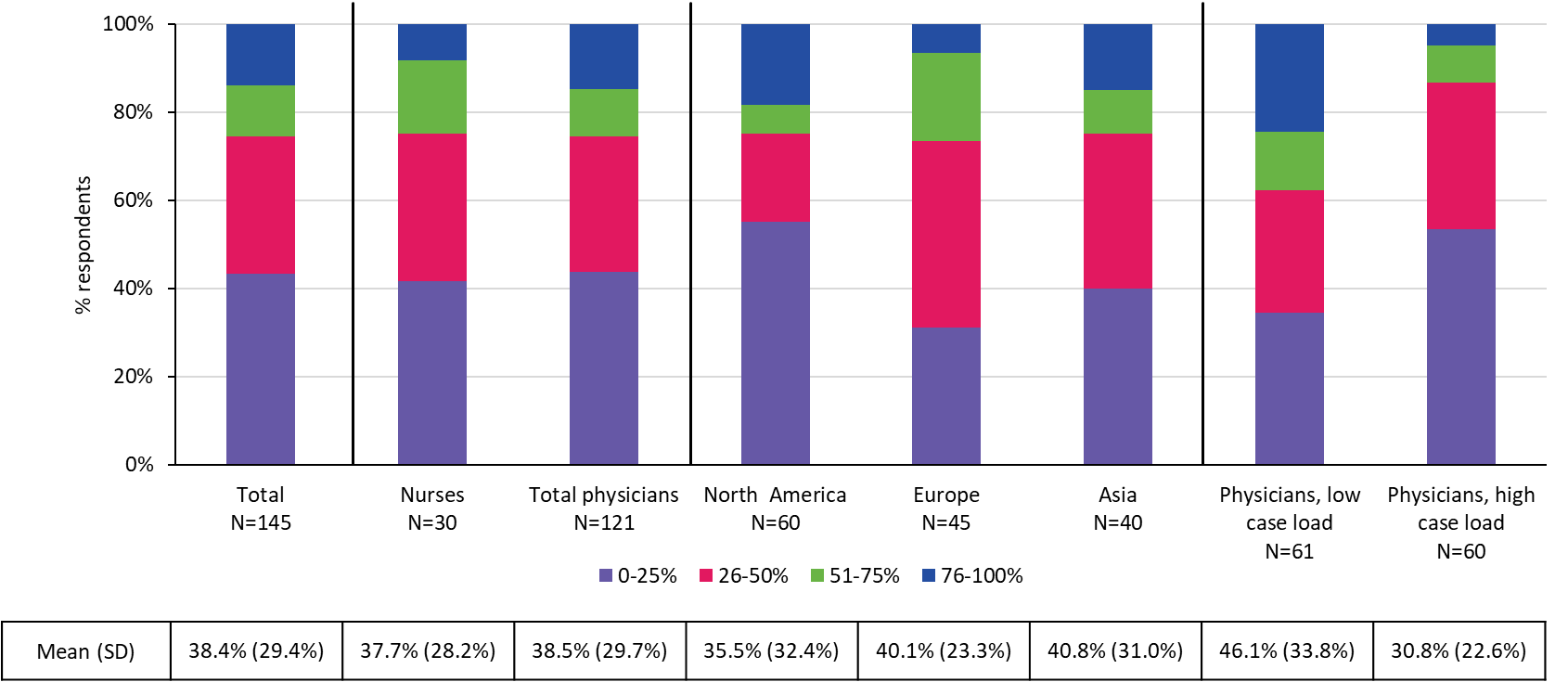


### Q8a. For patients who already have a diagnosis of PBC, where did this diagnosis typically happen?


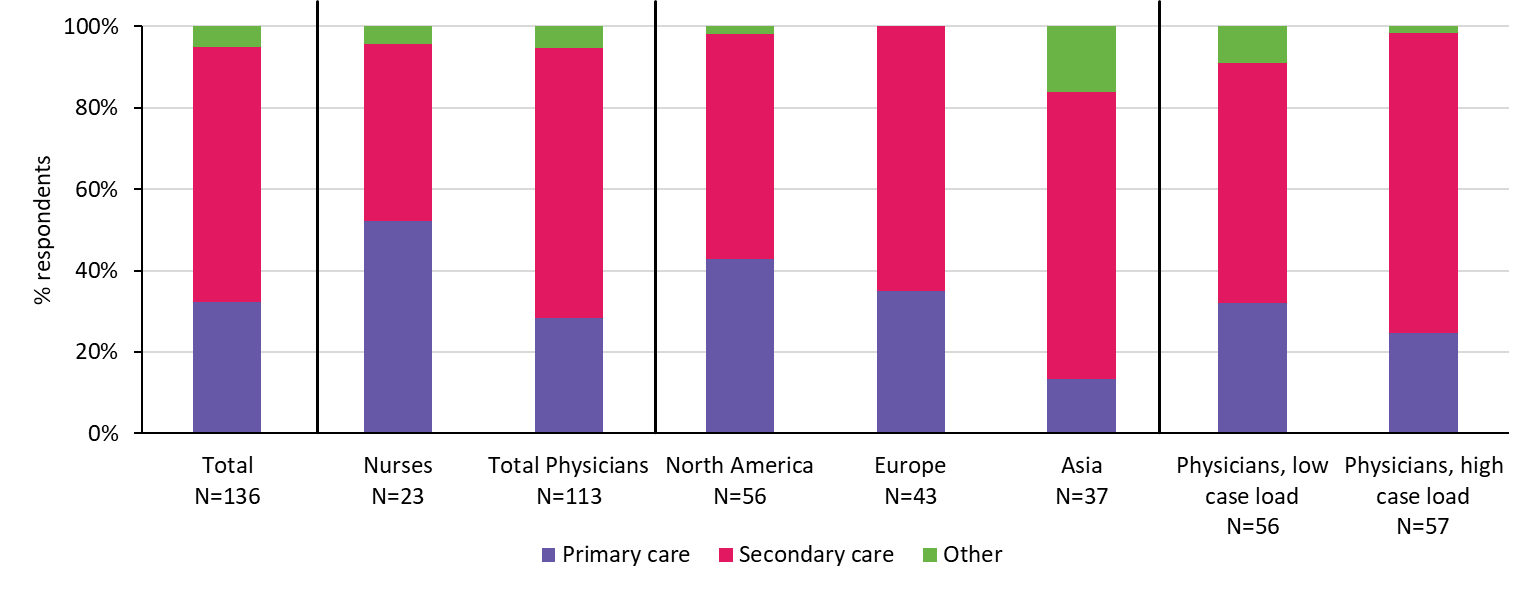


Other responses: tertiary hospital (N=5); other hospital (N=2).

### Q9. Which tests or assessments do you require to be confident of a diagnosis of PBC?


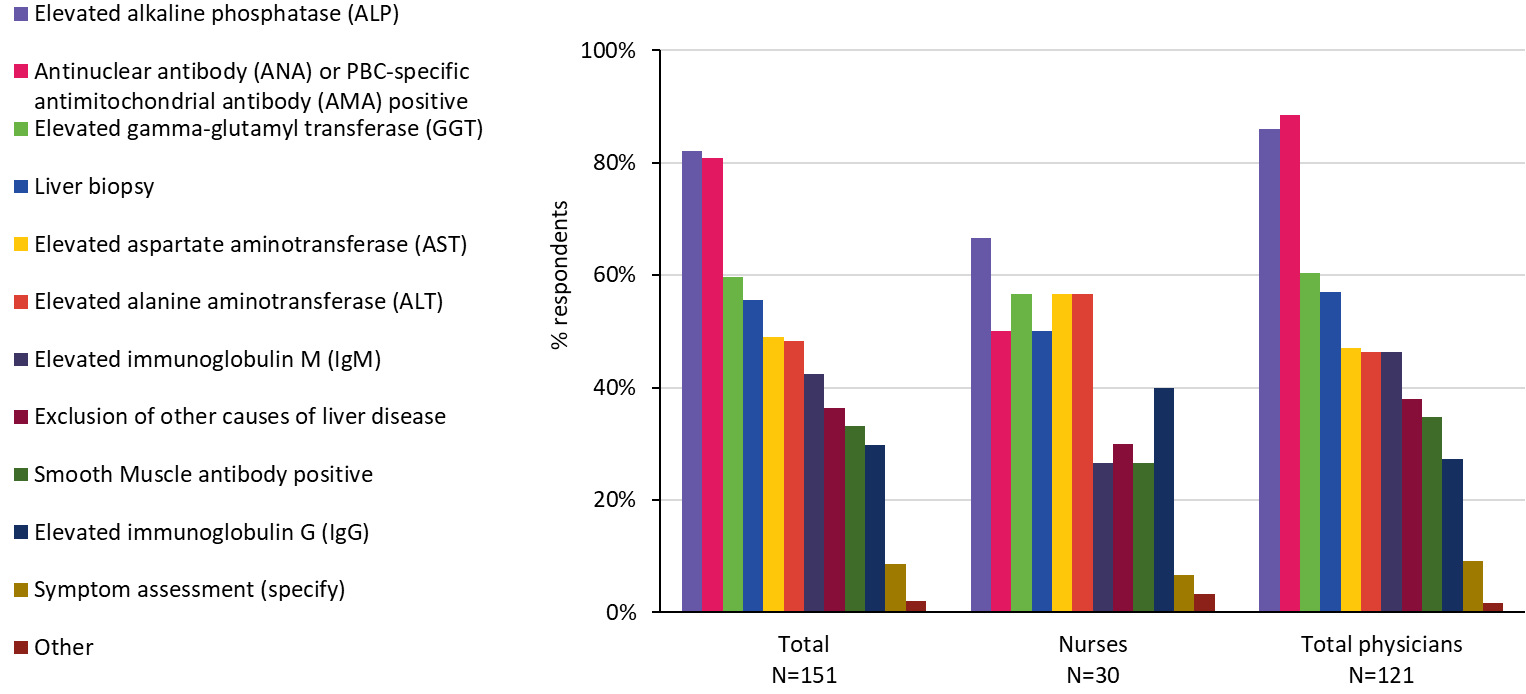


Symptoms identified to be confident of a PBC diagnosis: itch (N=9); fatigue (N=3); jaundice (N=3) and skin symptoms, fever, abdominal pain, fatty liver N=1 each. Other responses: liver ultrasound, elevated lipids;
anti GP210-antibody (N=1 each).


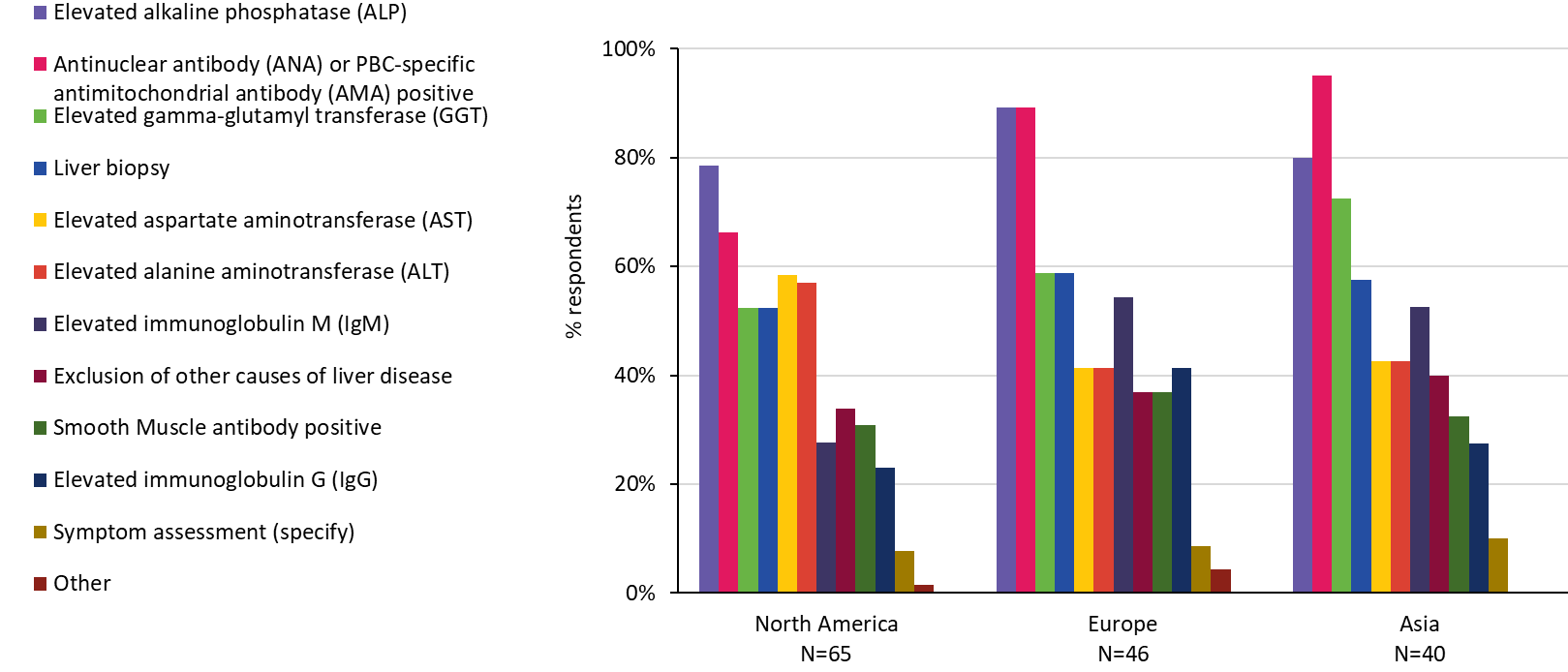


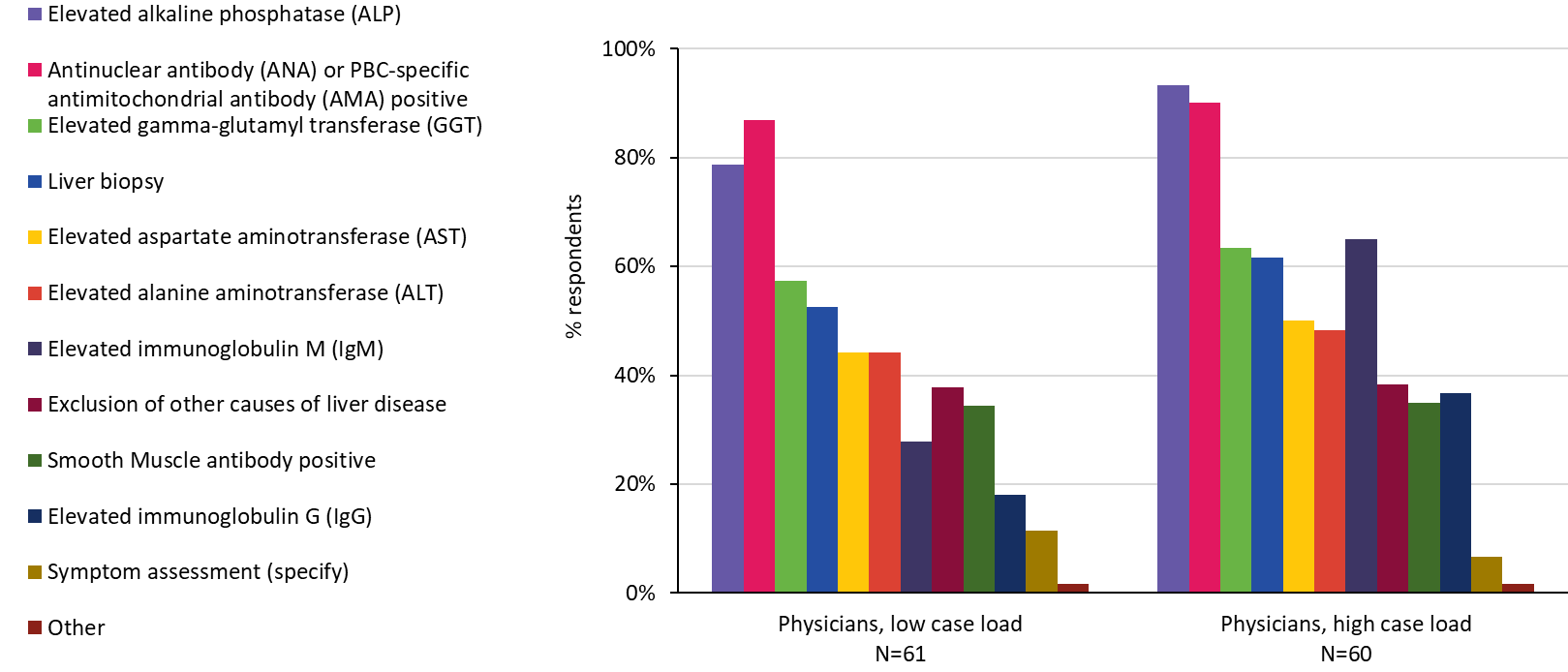


### Q10. For those patients who already have a diagnosis of PBC, do you typically require any tests to confirm the diagnosis of PBC?


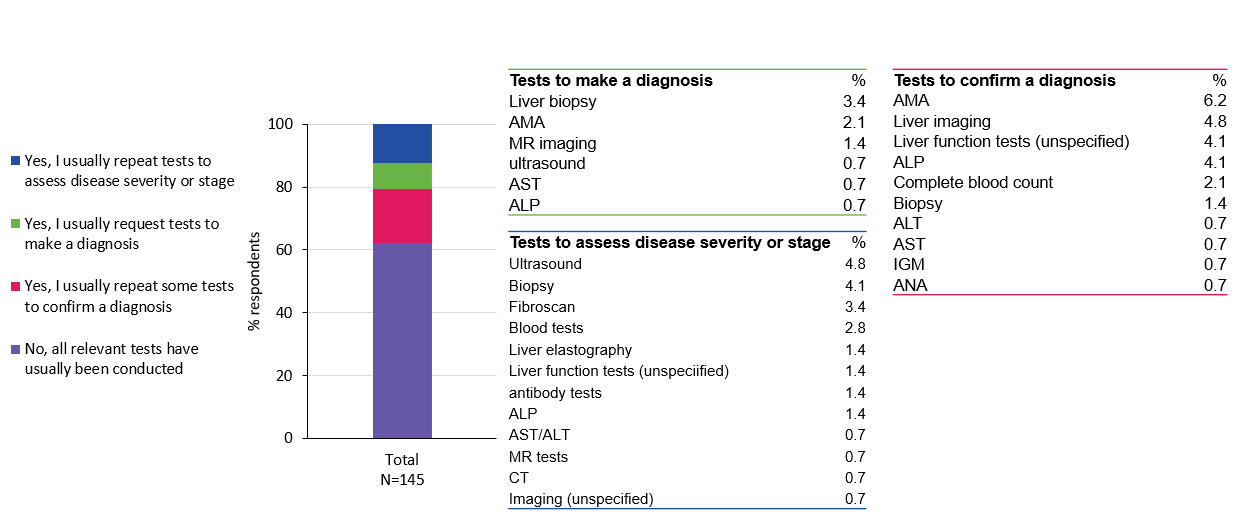


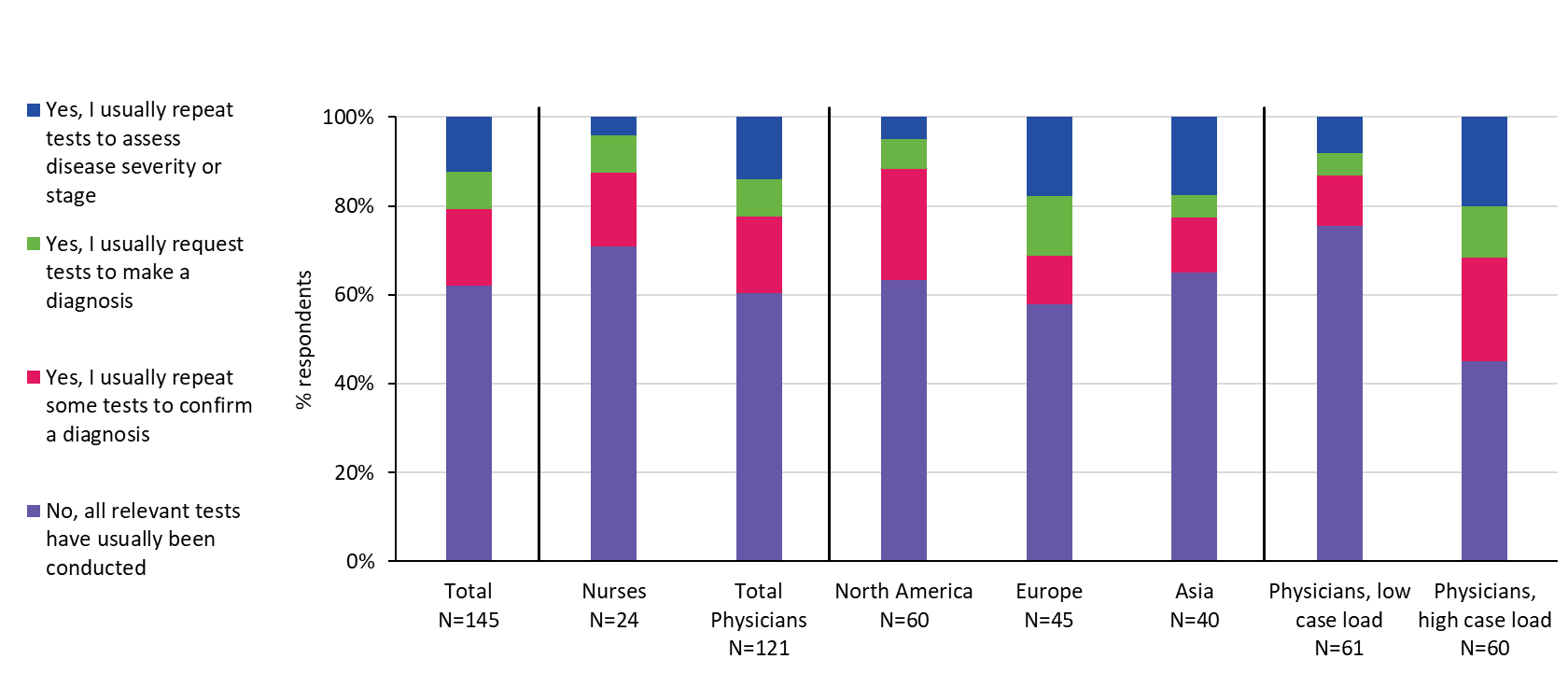


### Q11. What do you consider to be the primary driver(s) of prognosis for PBC?


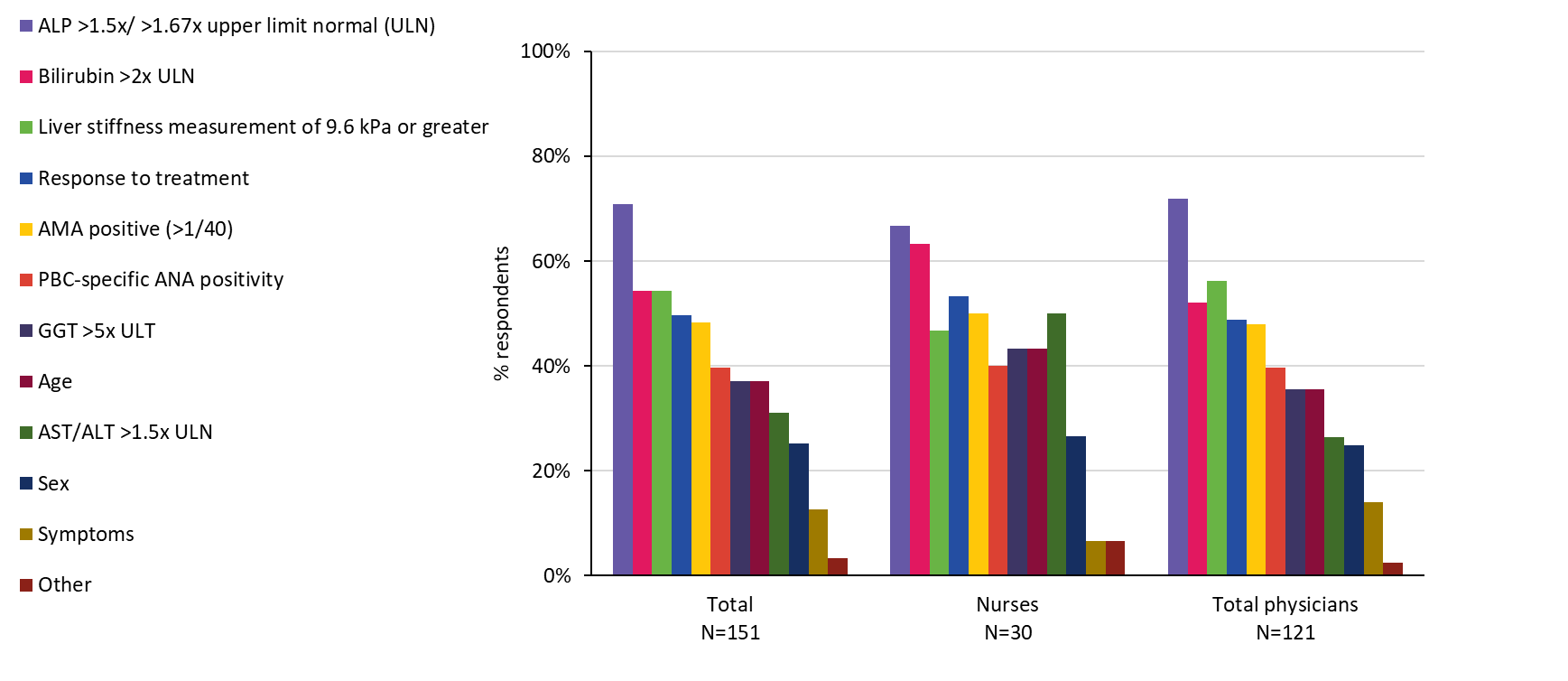


Other responses: albumin, N=2; smoking / drinking (N=2); obesity (n=1); comorbidities (N=1).


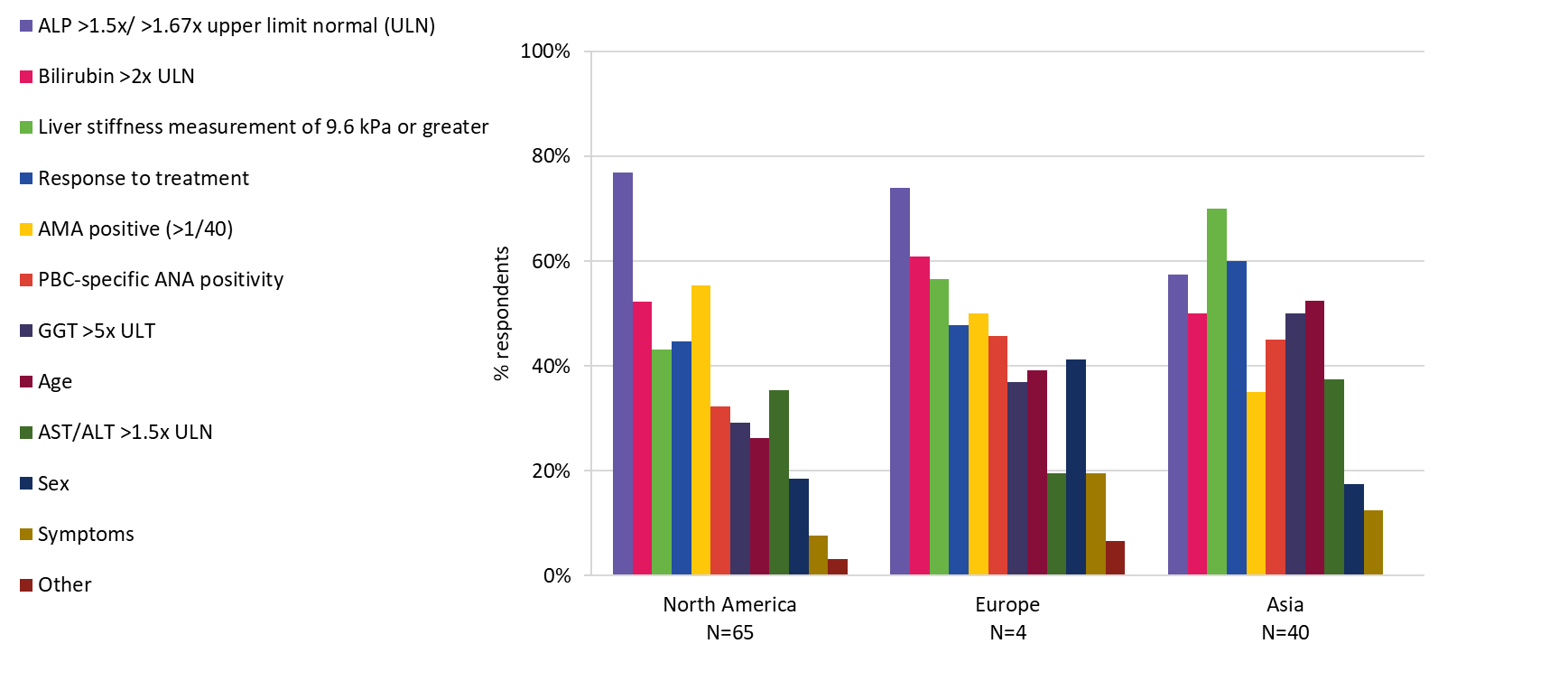


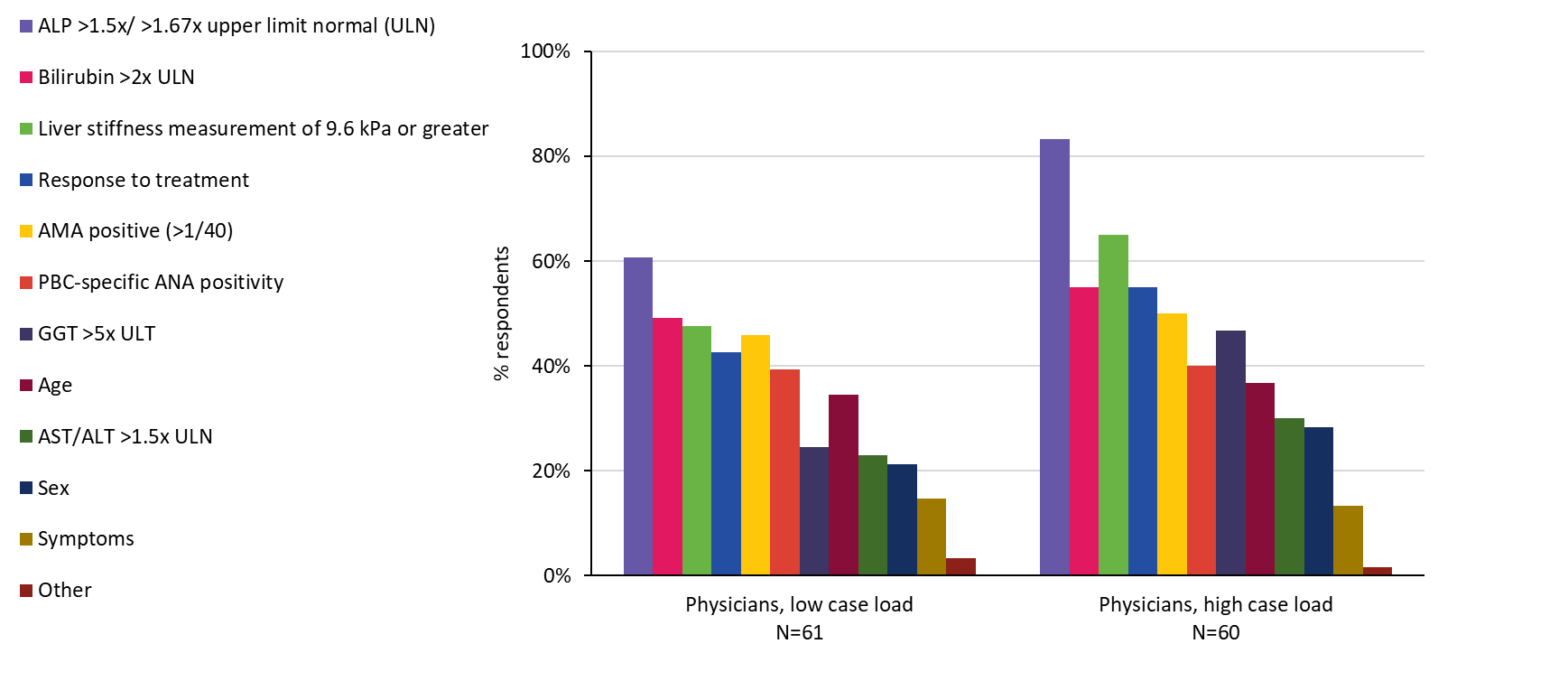


### Q12. Which of the following do you discuss with patients once you have established their diagnosis of PBC (at the first consultation with them)?


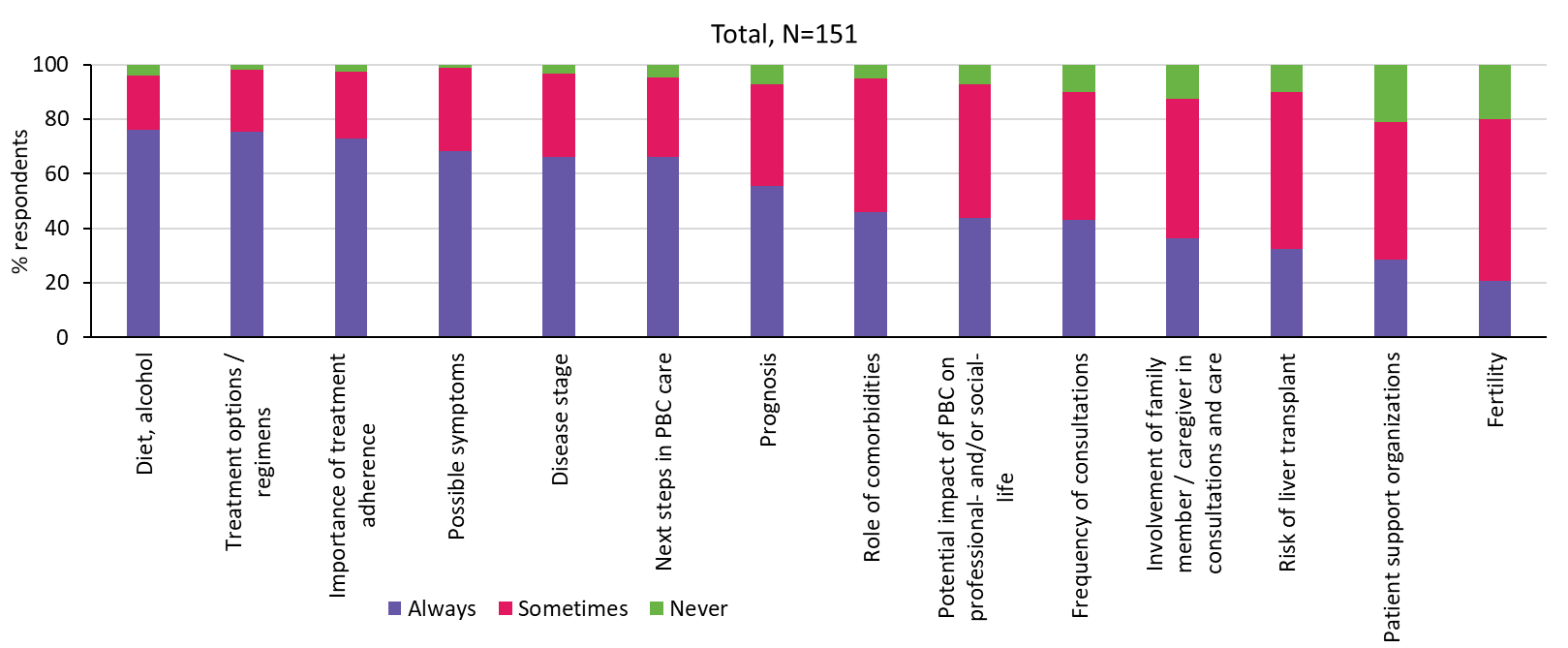


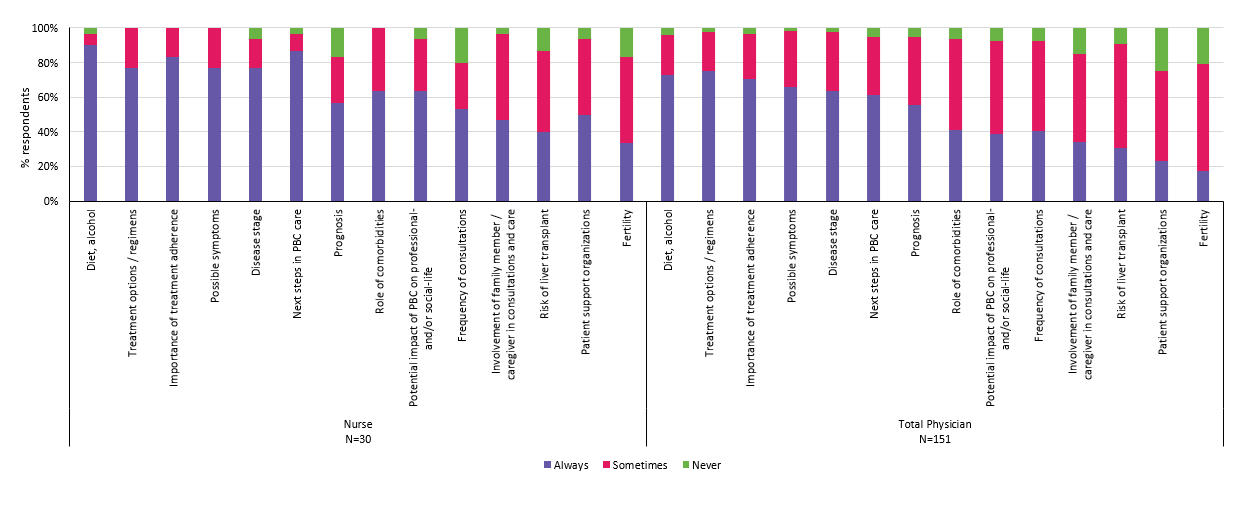


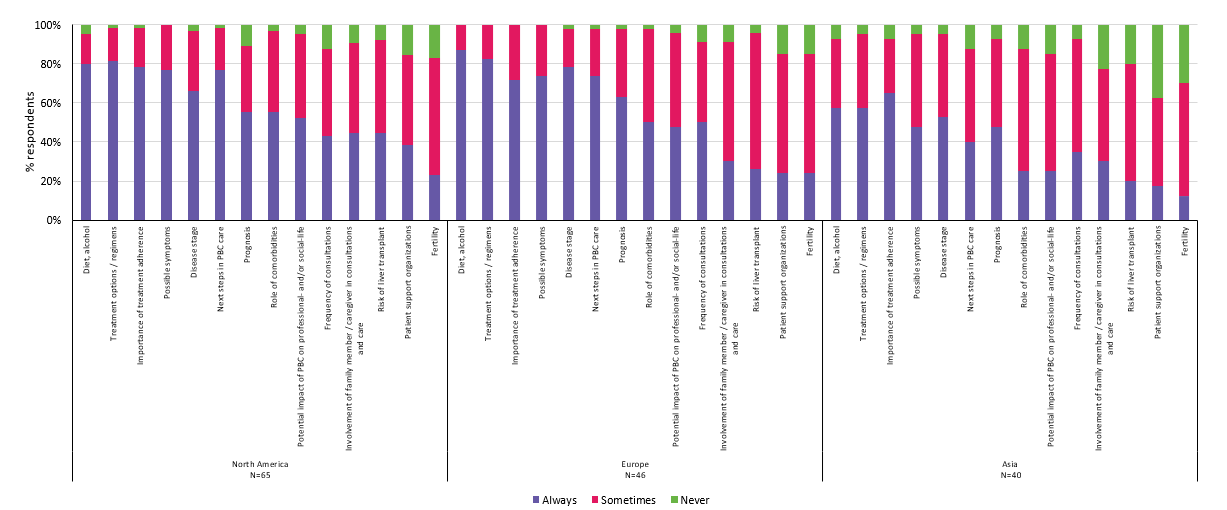


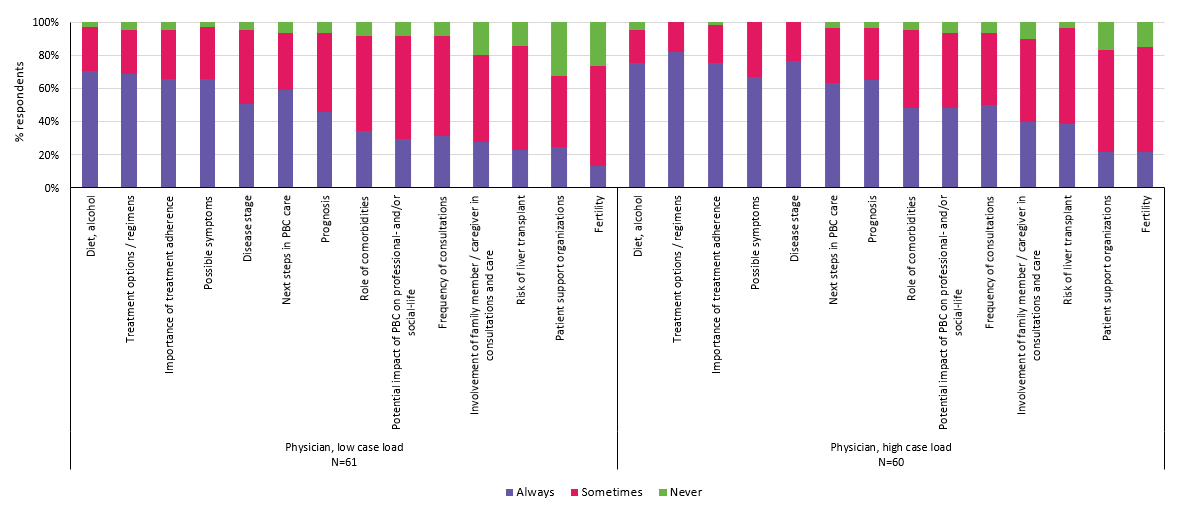


### Q13. At the first consultation with patients when you established their diagnosis of PBC, do you proactively ask patients about whether or not they have symptoms?


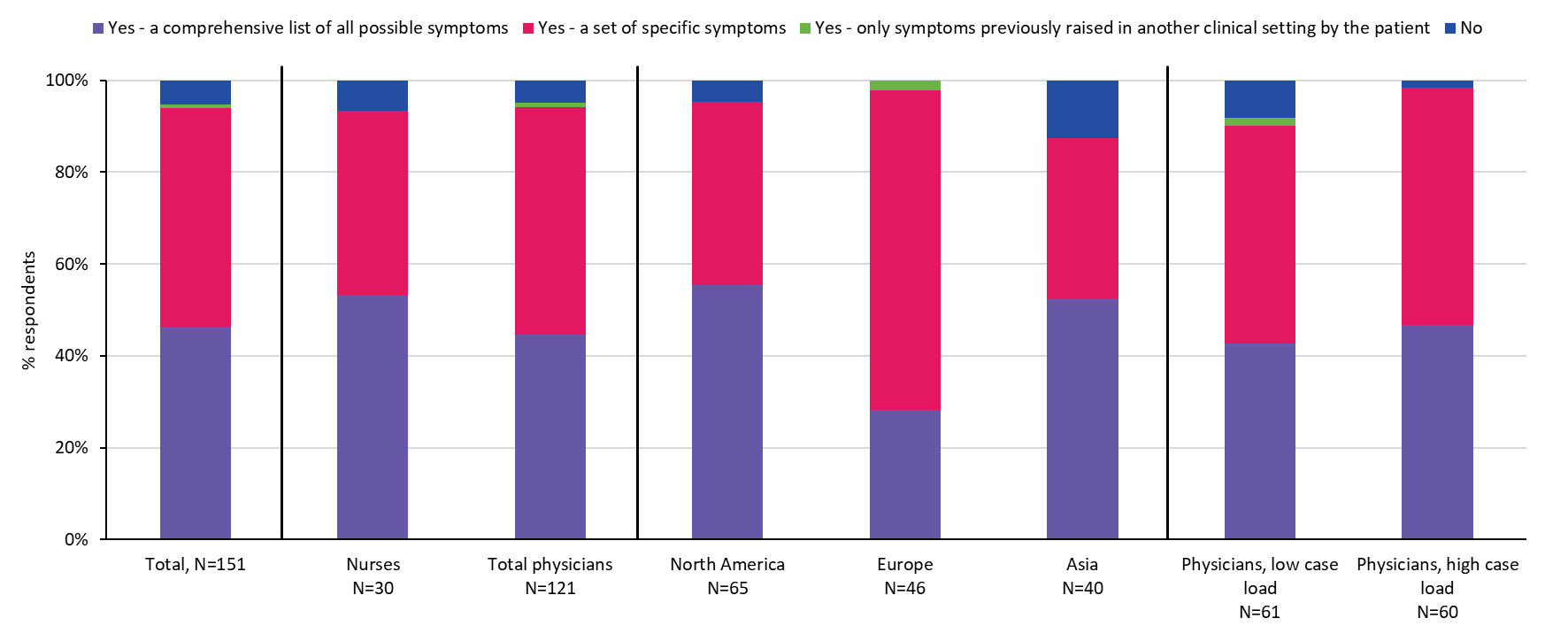


### Q14. At the first consultation with patients when you established their diagnosis of PBC, which symptoms do you discuss with patients?


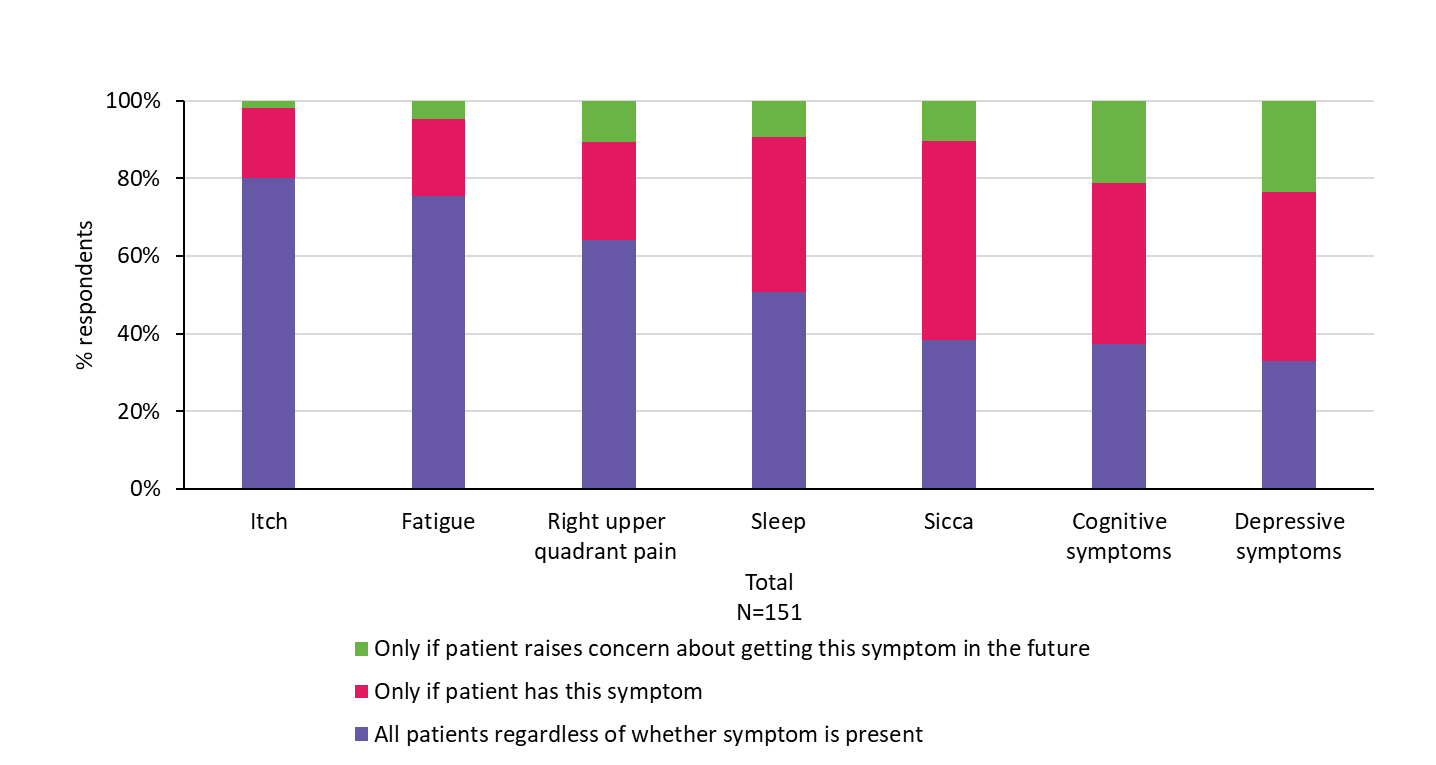


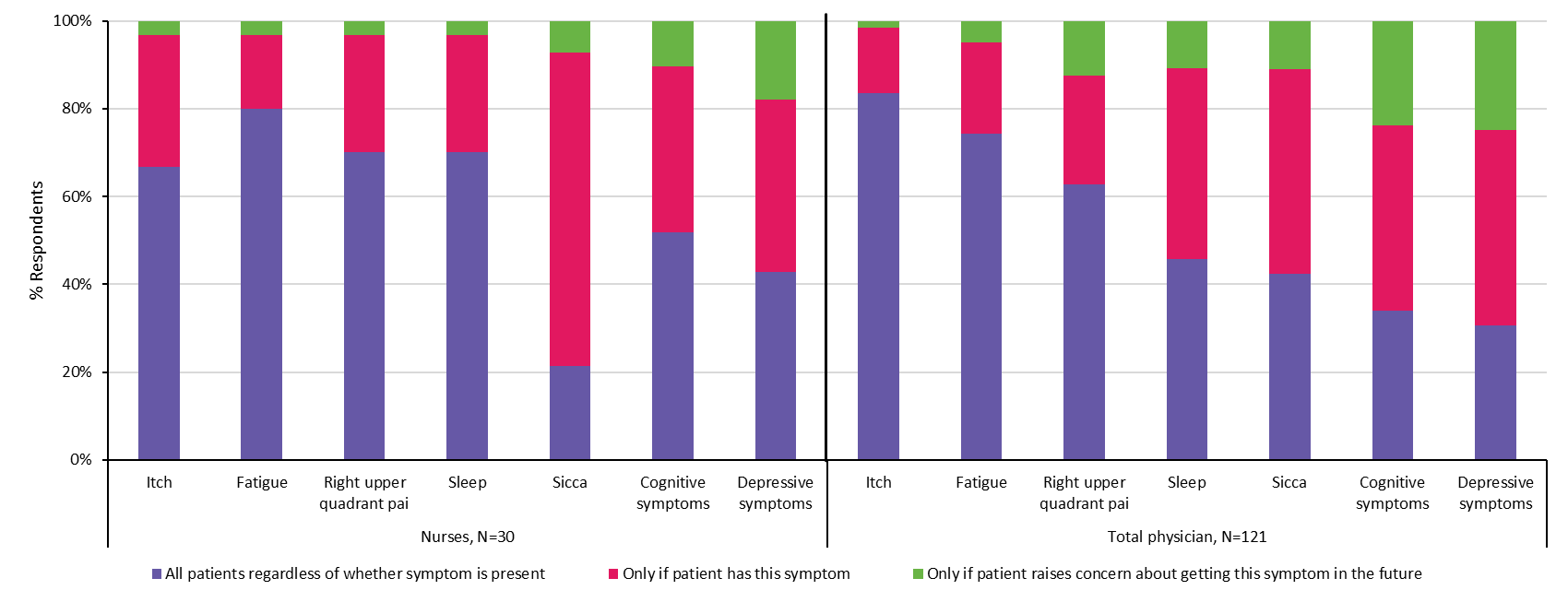


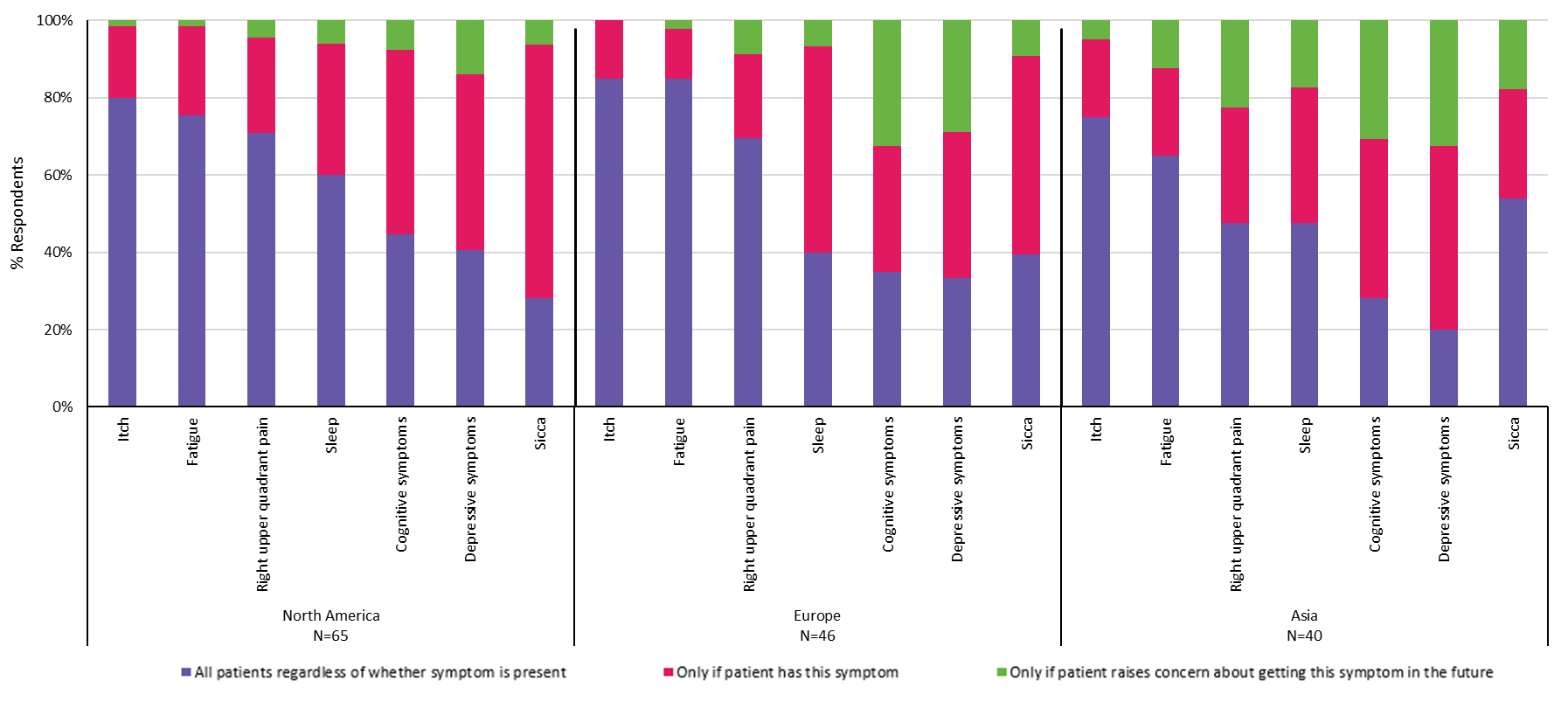


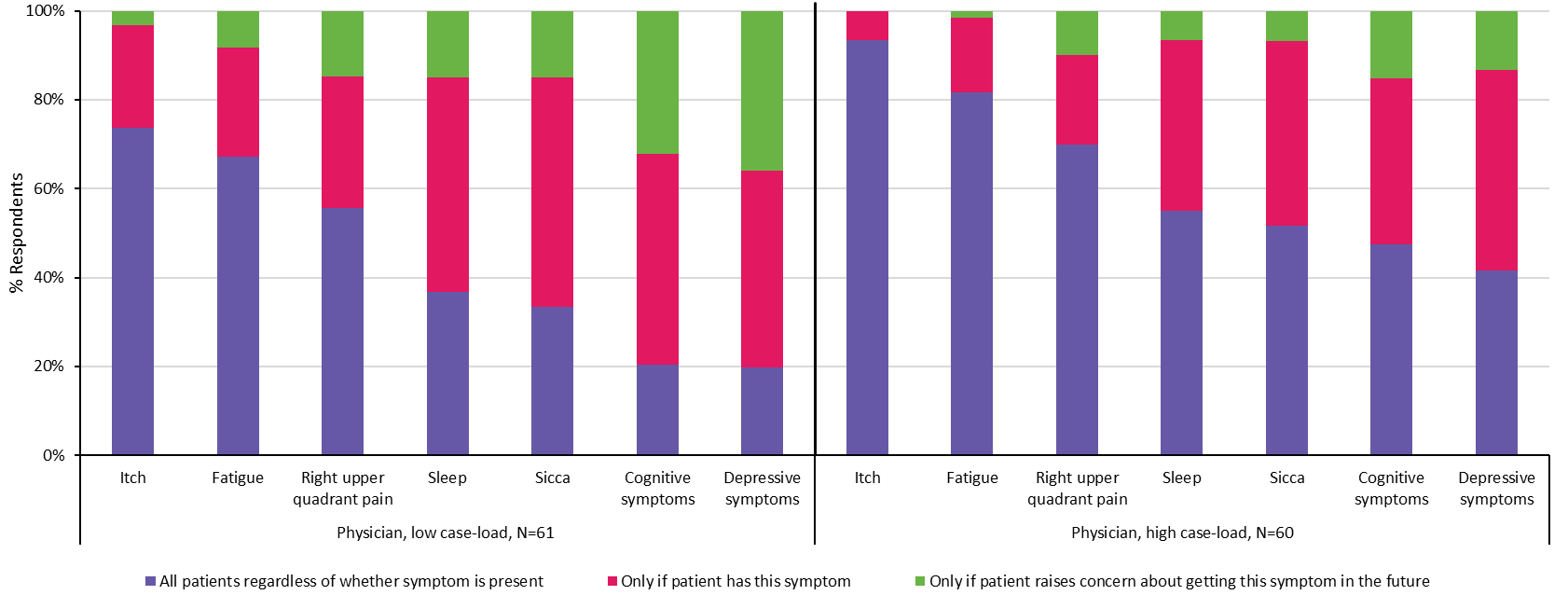


### Q15. Do you discuss that symptoms may appear or change over the course of the disease?


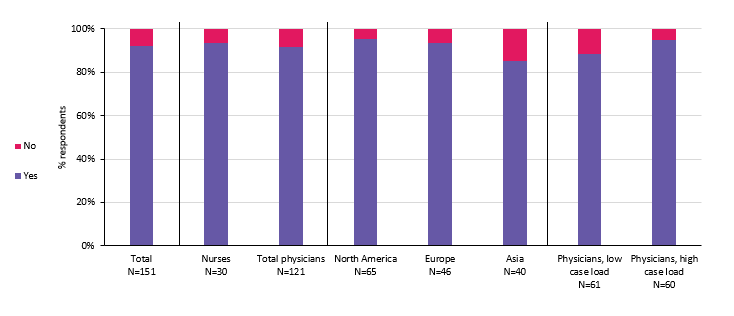


### Q16. When discussing symptoms, do you make patients aware that the control of their disease (in response to treatment) might not correlate with the resolution of any symptoms which may be present?


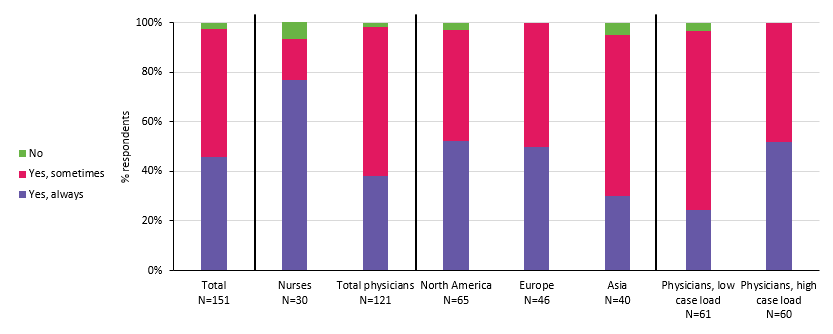


### Q17. When discussing a PBC management plan with patients, which of the following do you include?


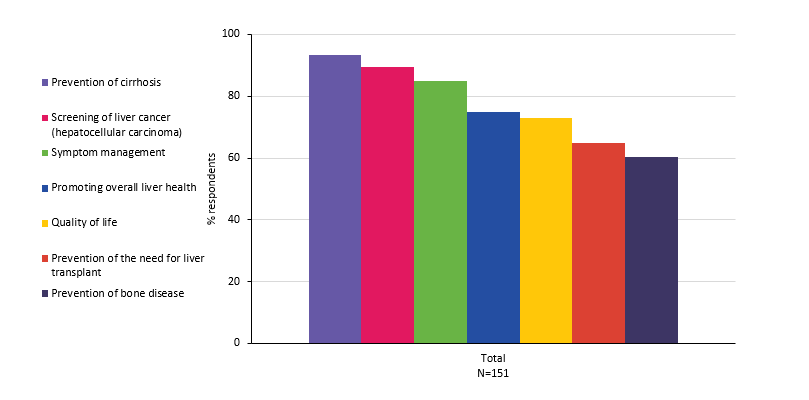


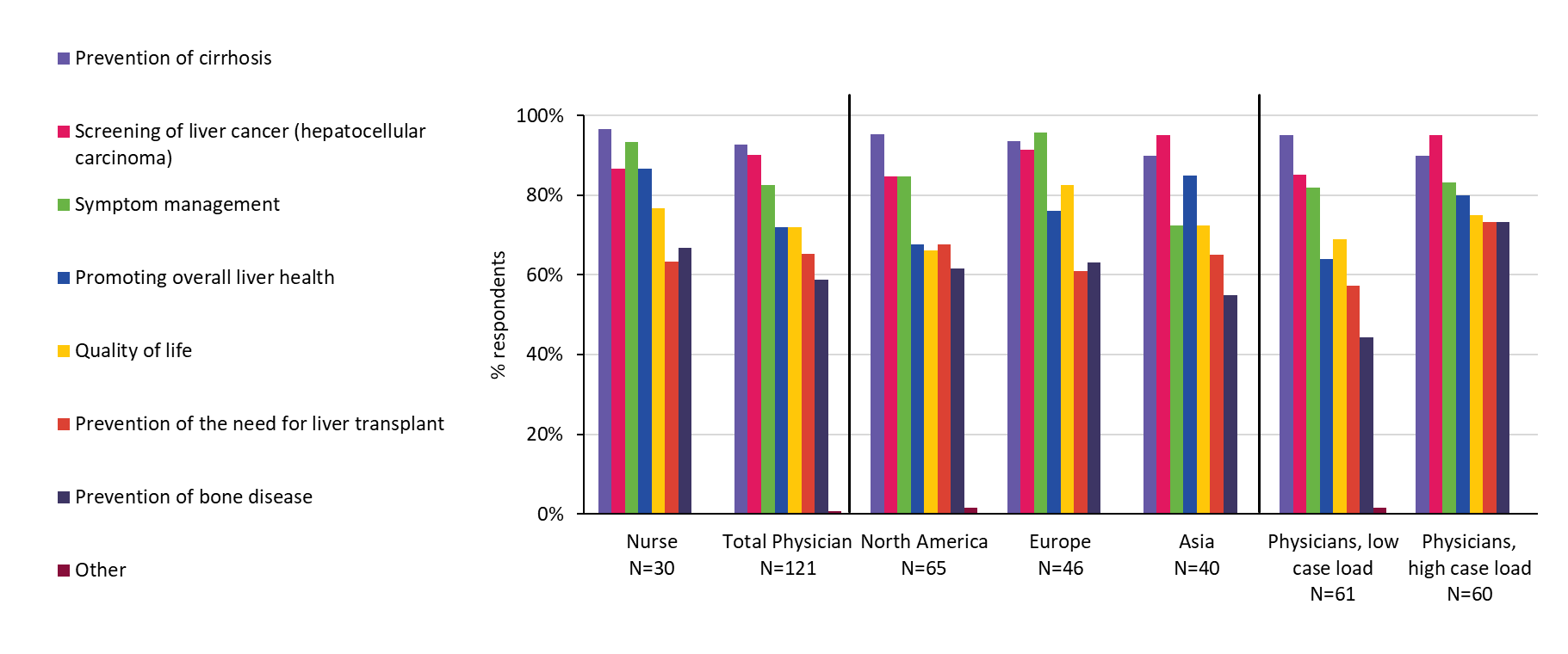


### Q18. Do you discuss a monitoring plan with patients?


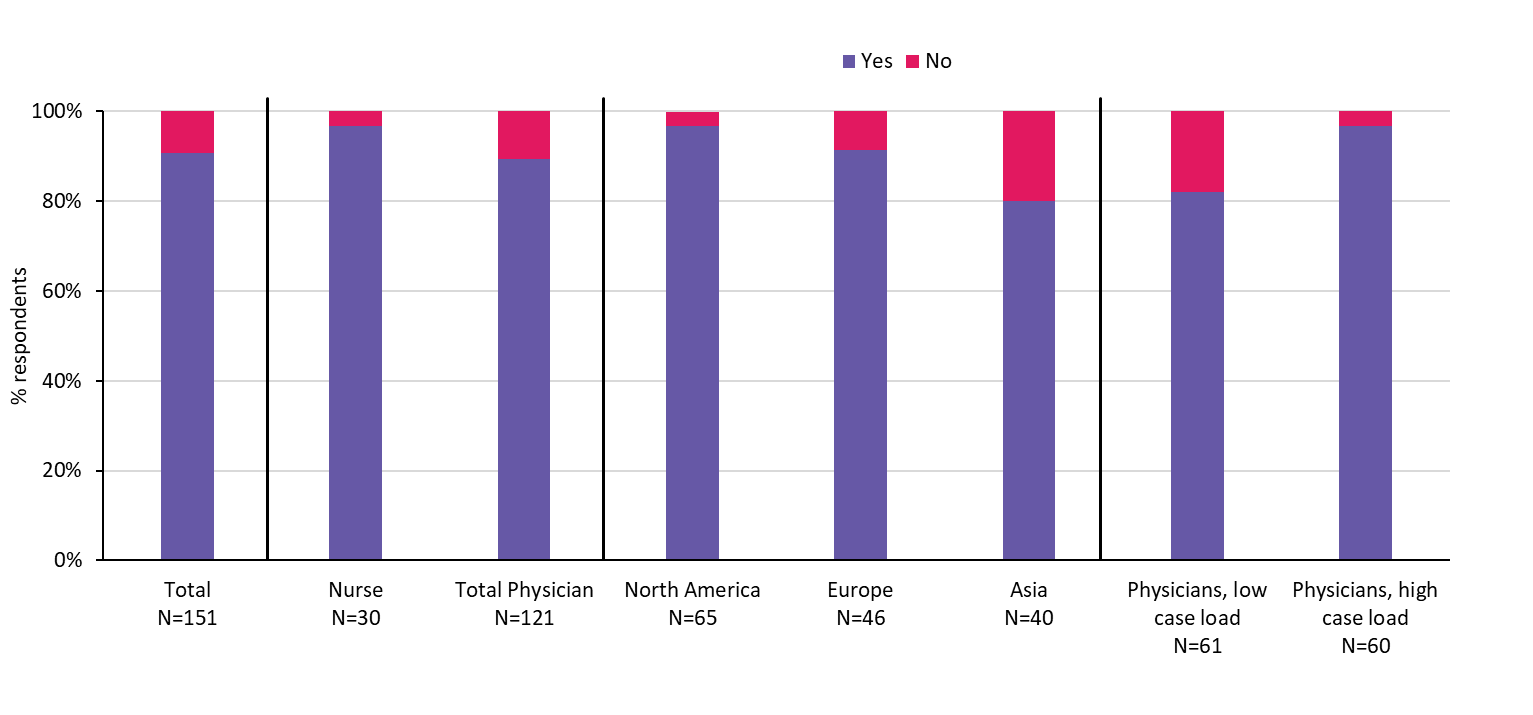


### Q18a. Do you discuss a monitoring plan with patients? If yes, which of the following potential tests do you discuss?


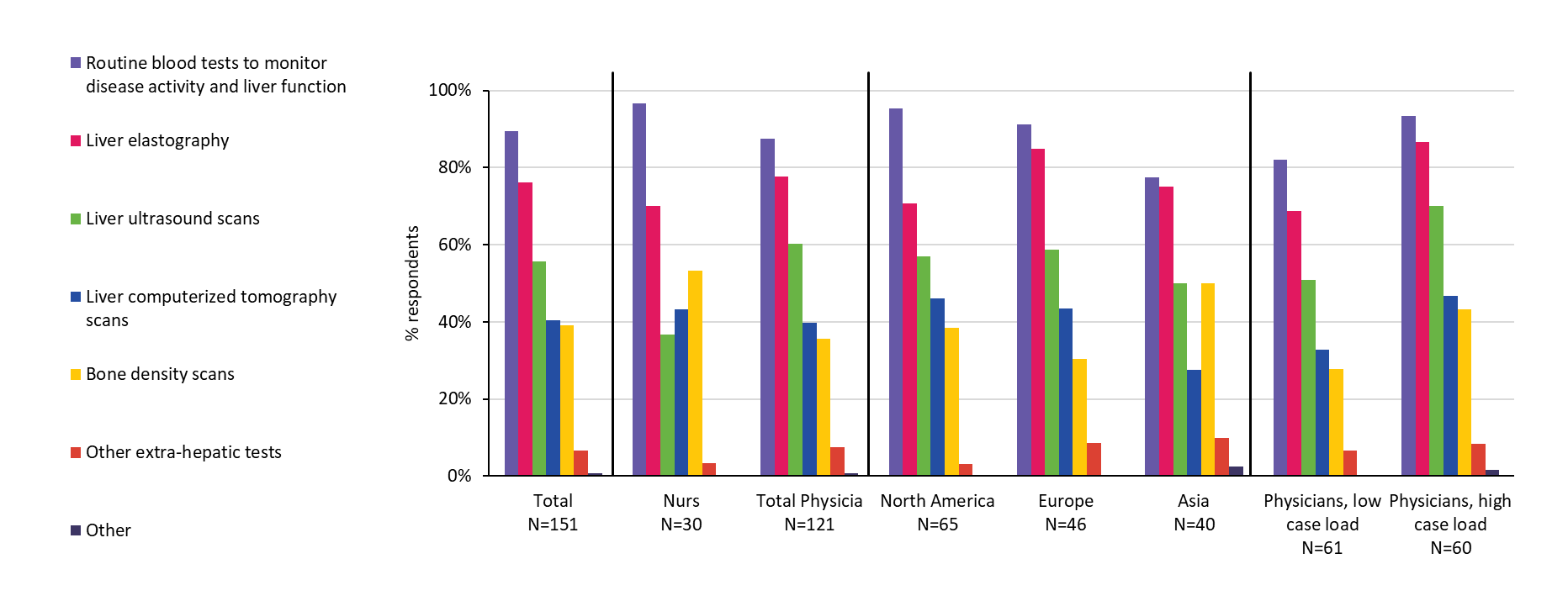


### Q19. During this first interaction, do you provide or direct patients to educational materials about PBC?


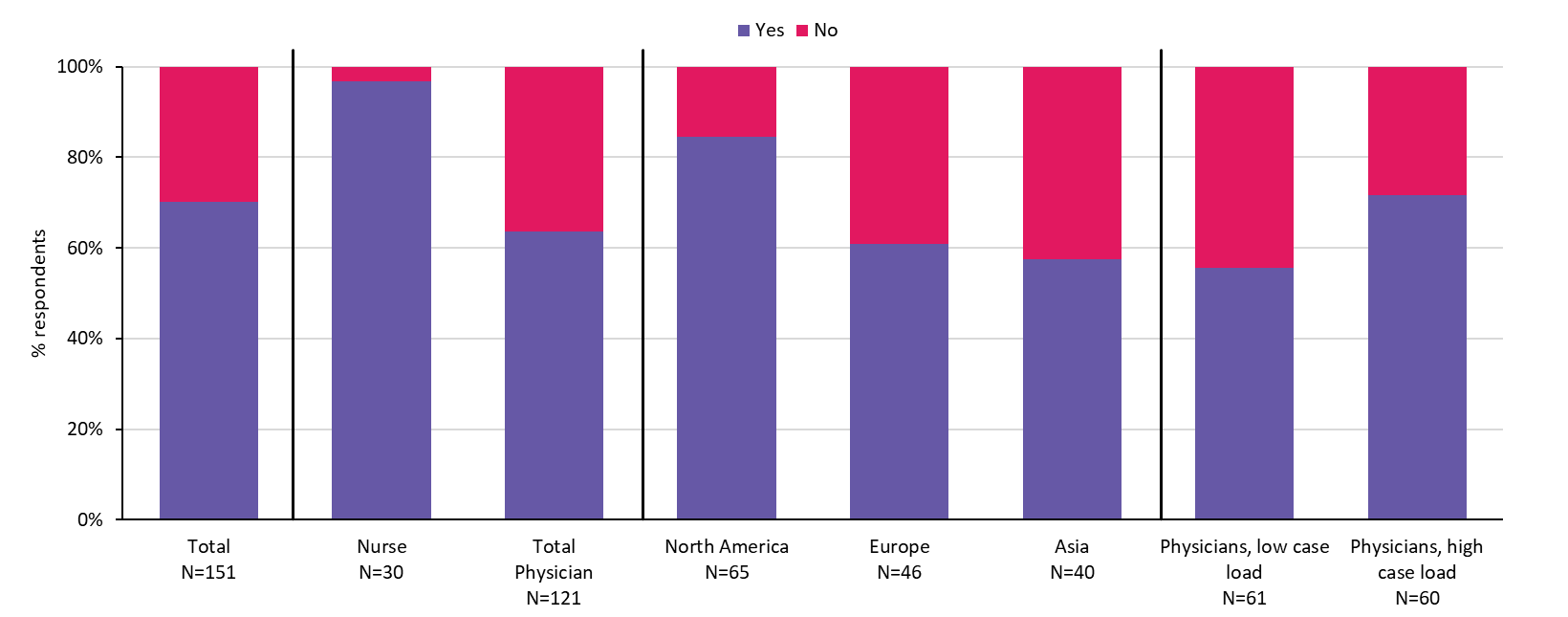


### Q19a. During this first interaction, do you provide or direct patients to educational materials about PBC? If yes, what do the materials cover?


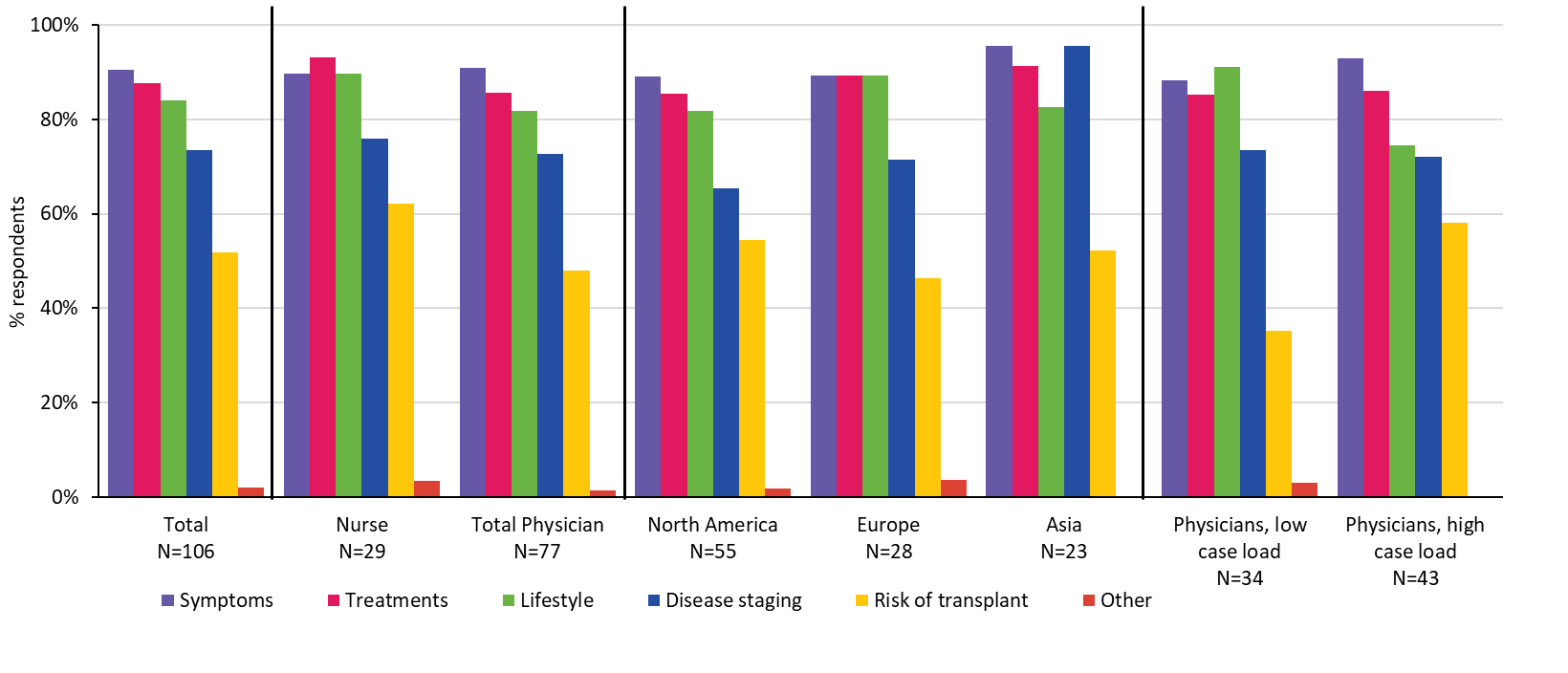


Other responses: support groups (N=2), referral to other care providers (N=1).

### Q19b. During this first interaction, do you provide or direct patients to educational materials about PBC? If no, why not?


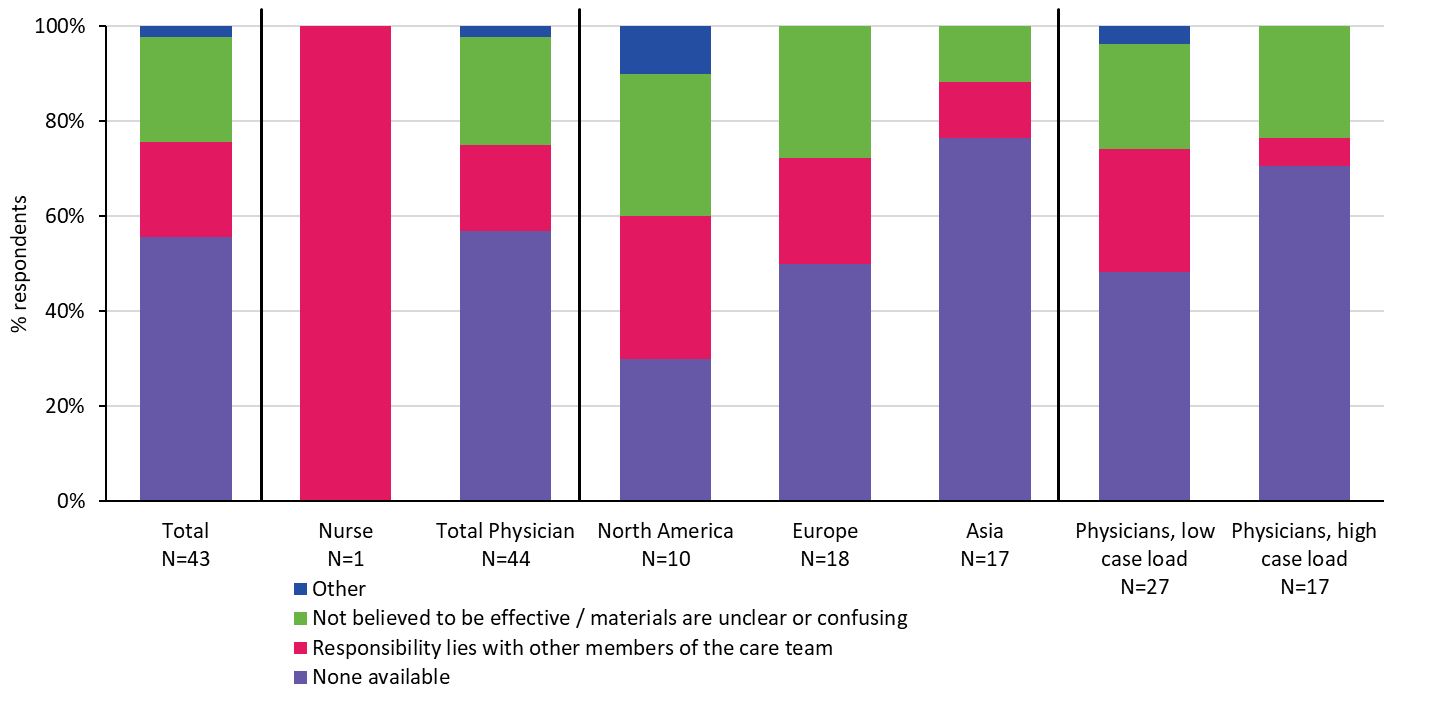


Other responses: difficult to produce materials (N=1), not aware of any materials (N=1).

## Symptom assessment and ongoing management

### Q20. What would you consider routine follow-up for patients with PBC (acknowledging that this may vary depending on disease-specific factors such as treatment response, disease course and stage)?


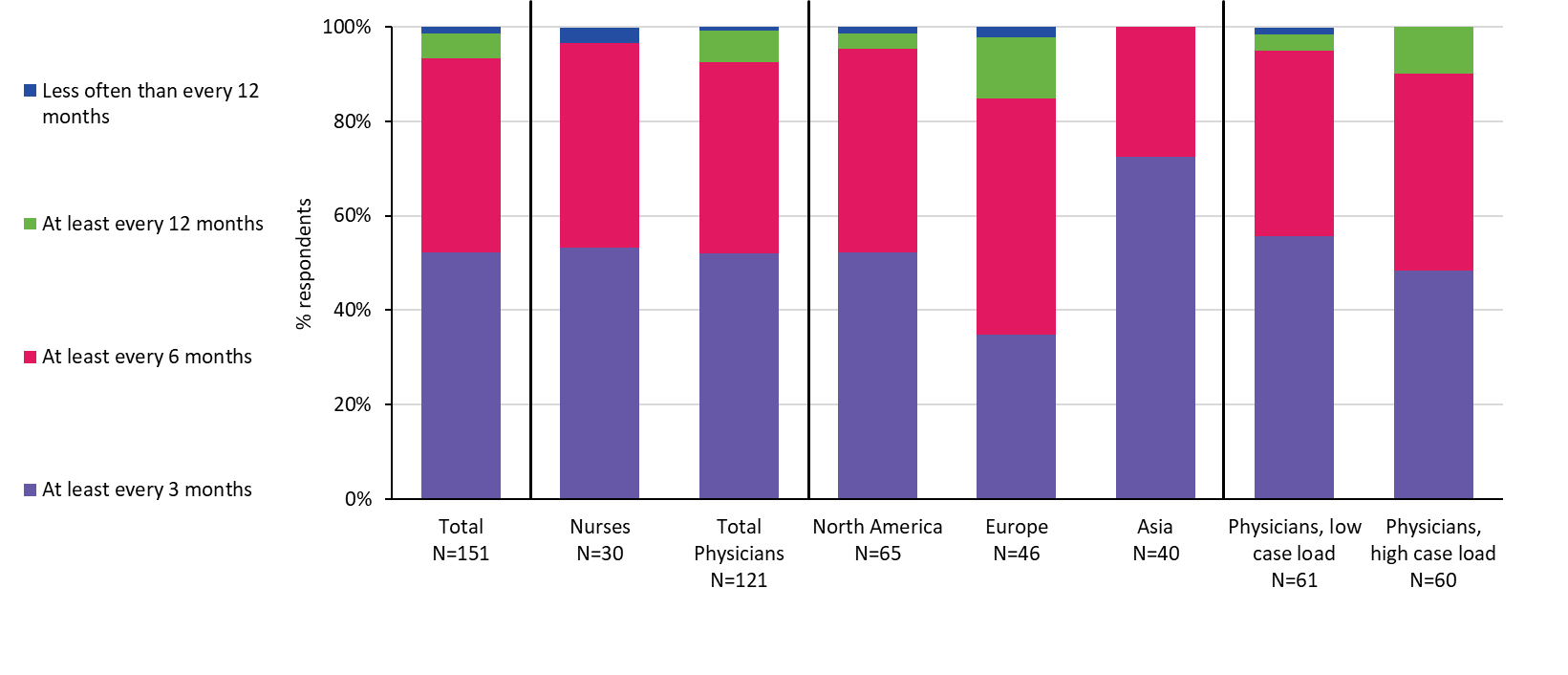


### Q21. In advance of a routine patient consultation, what action do you take?


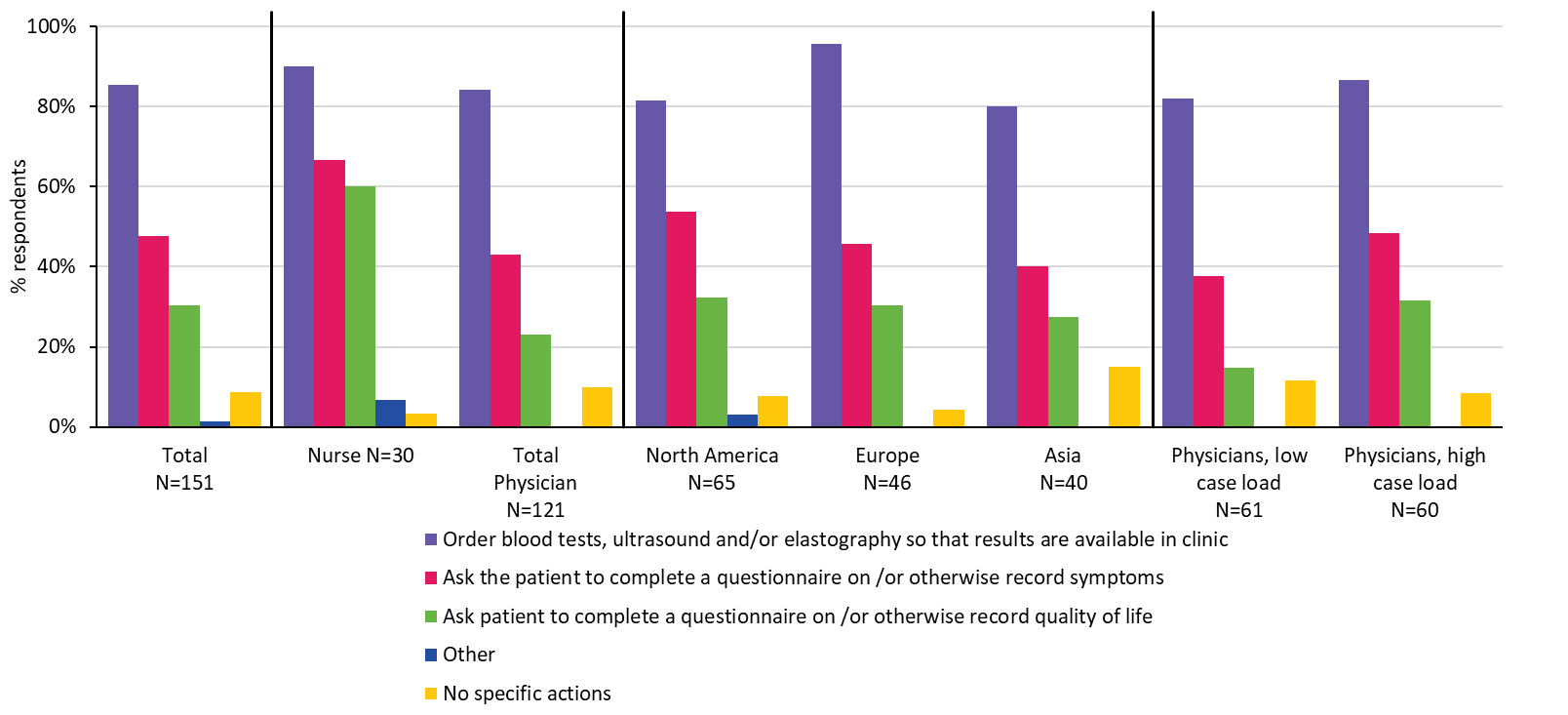


Other responses: review symptoms and lifestyle, medication adherence between appointments, review information before discharge from acute setting (N=1 each).

### Q22. Do you evaluate symptoms during routine consultations with patients?


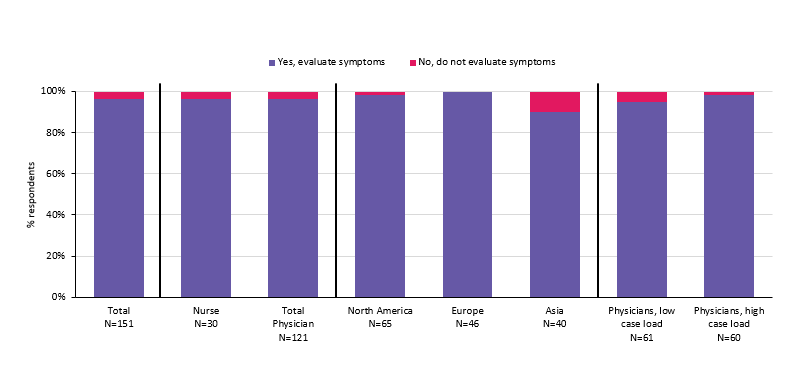


### Q22a. Do you evaluate symptoms during routine consultations with patients? If yes, how do you evaluate symptoms?


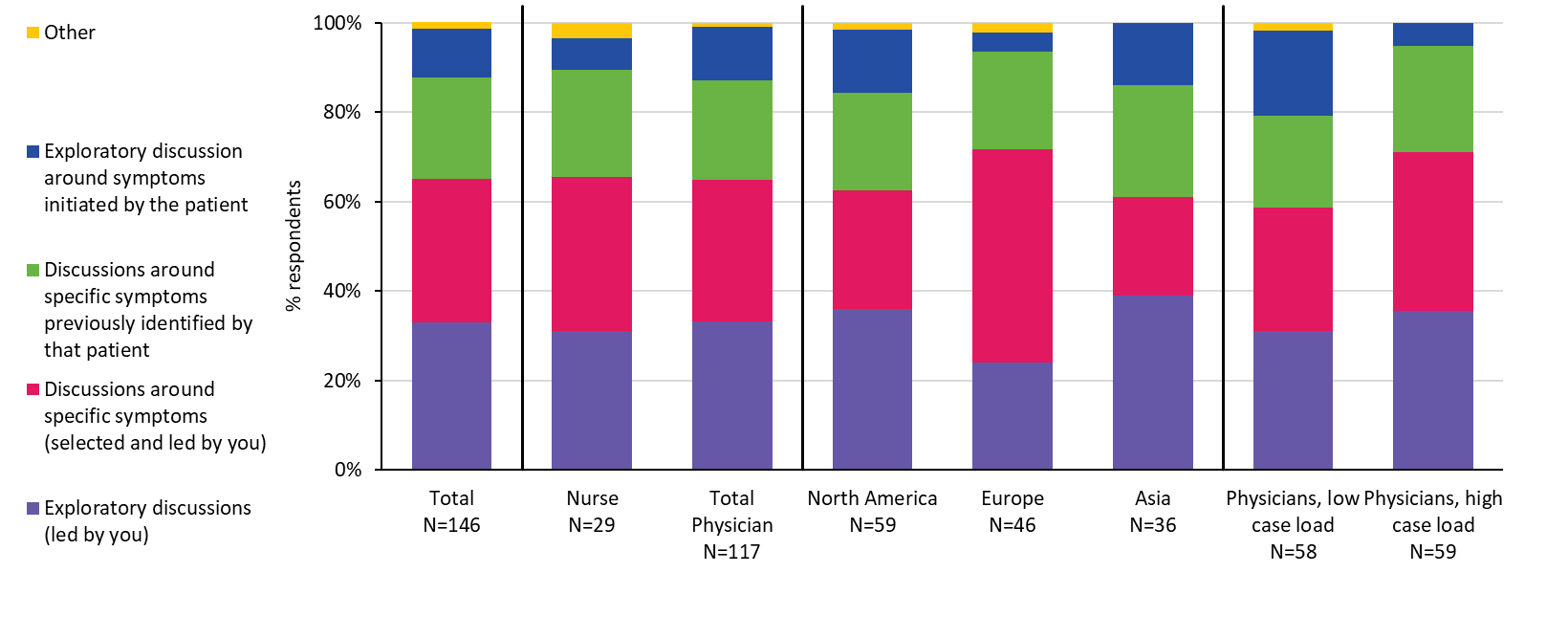


Other responses: mixed approach, always ask about fatigue and itching (N=1 each).

### Q22b. If you evaluate symptoms during routine consultations with patients, how do you evaluate symptom severity?


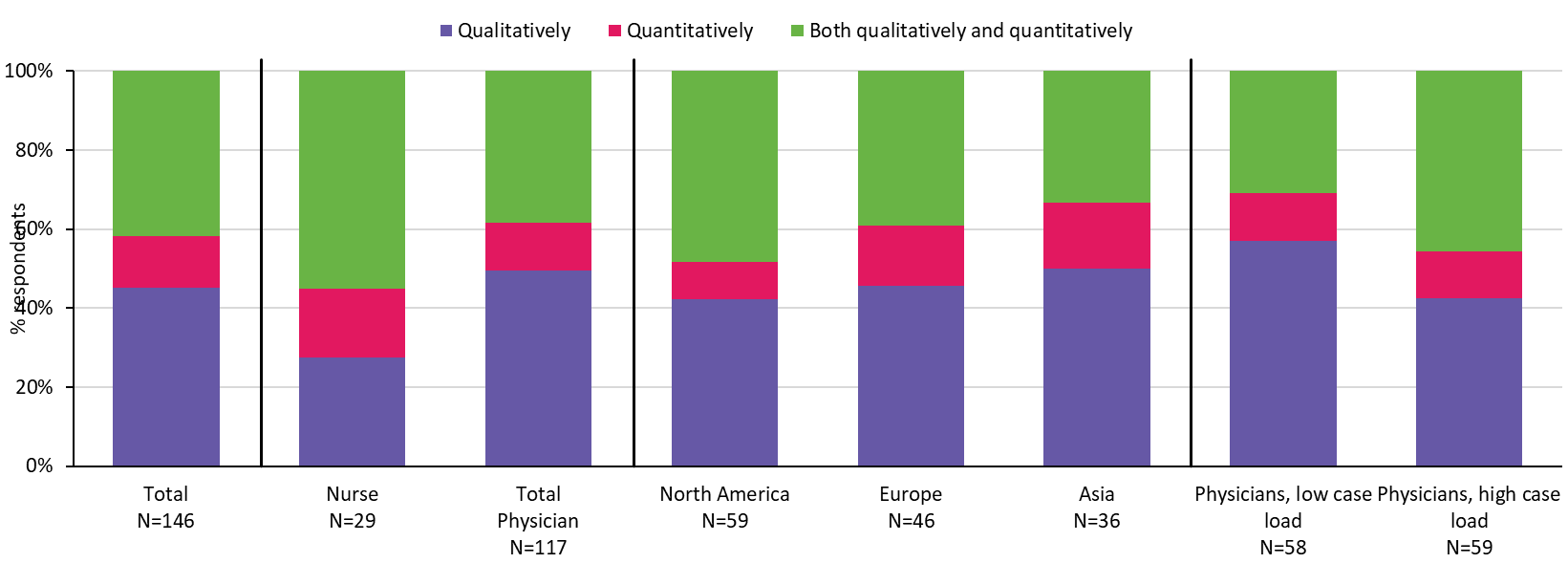


### Q22c. If you do evaluate symptoms during consultations, how do you assess changes over time?


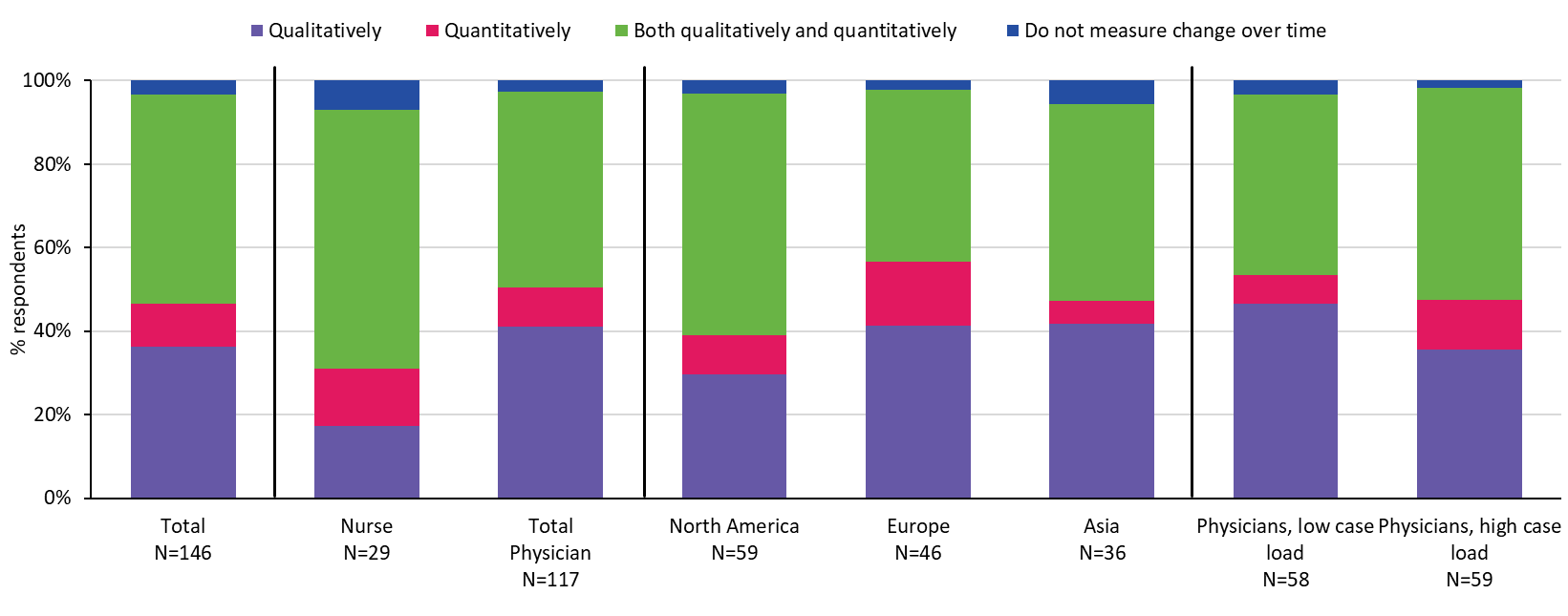


### Q23. Are you familiar with any formal tools or scales to measure symptoms and / or the impact of PBC on patients?


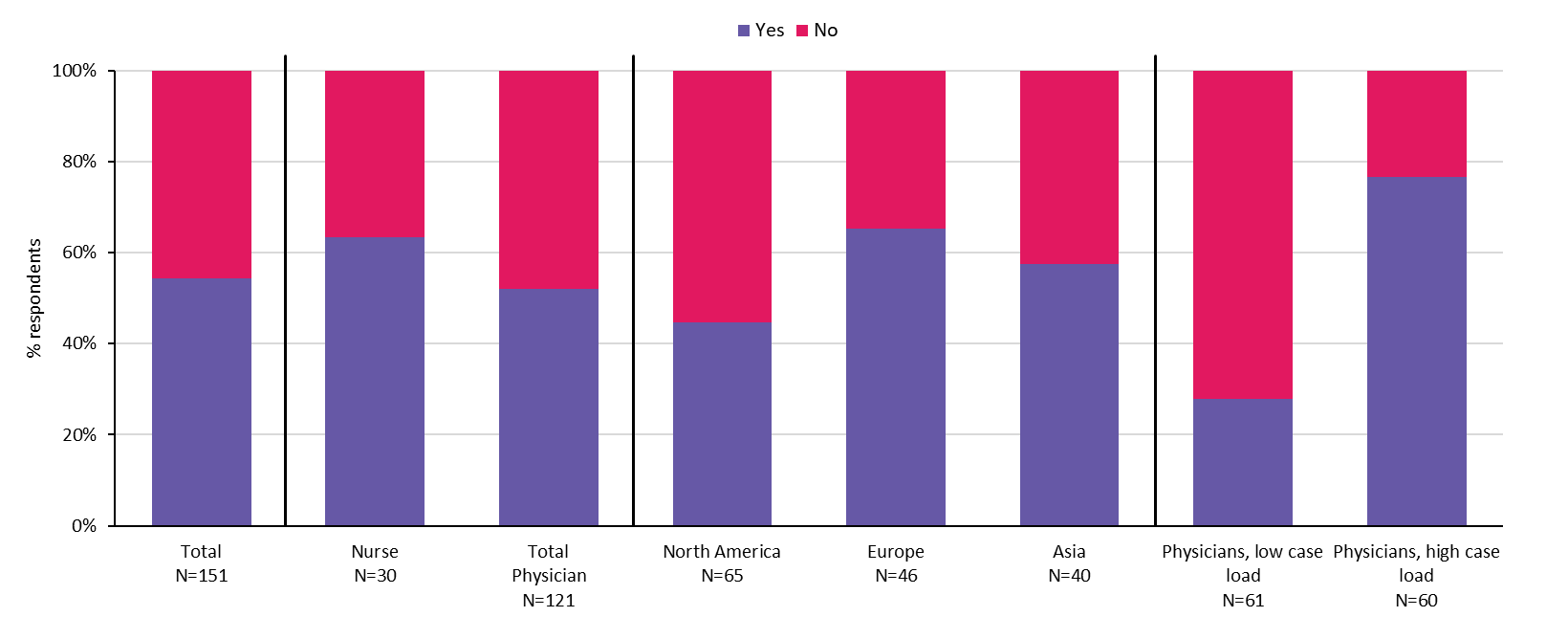


### Q23a. Are you familiar with any formal tools or scales to measure symptoms and / or the impact of PBC on patients? If so, which tools?


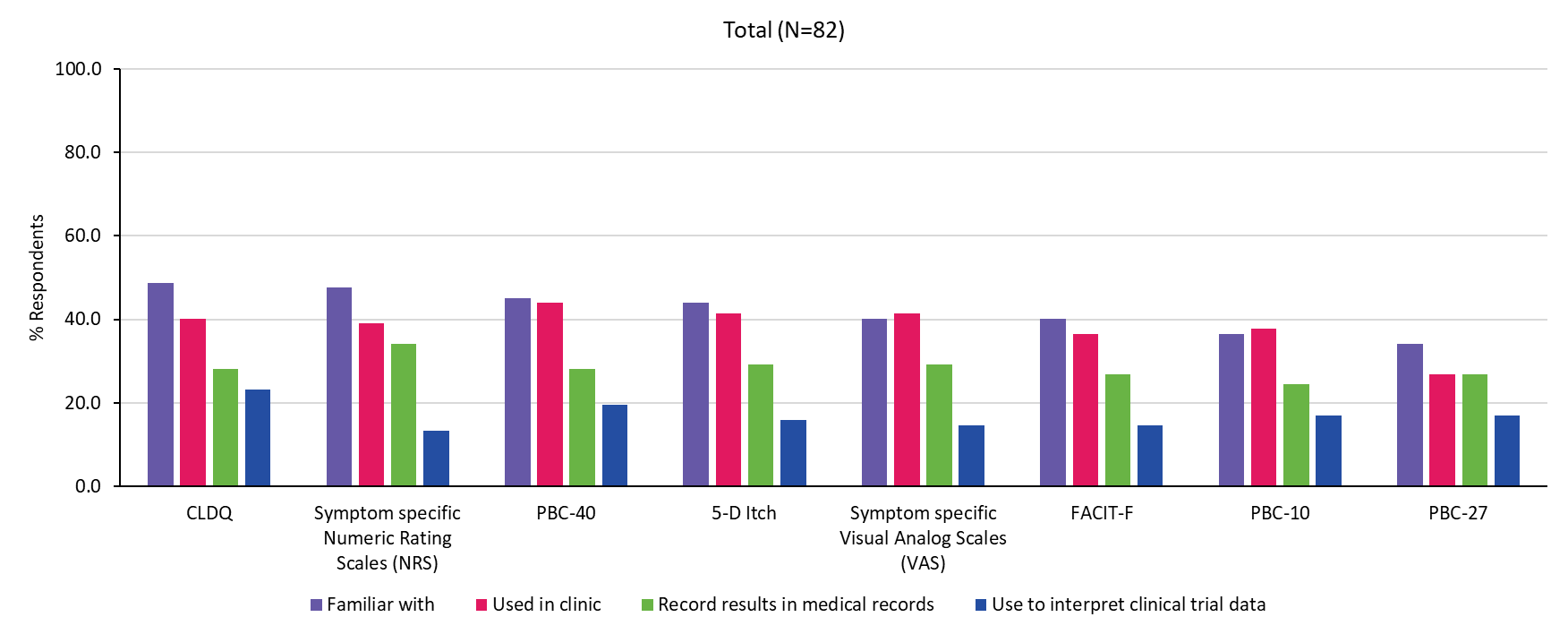


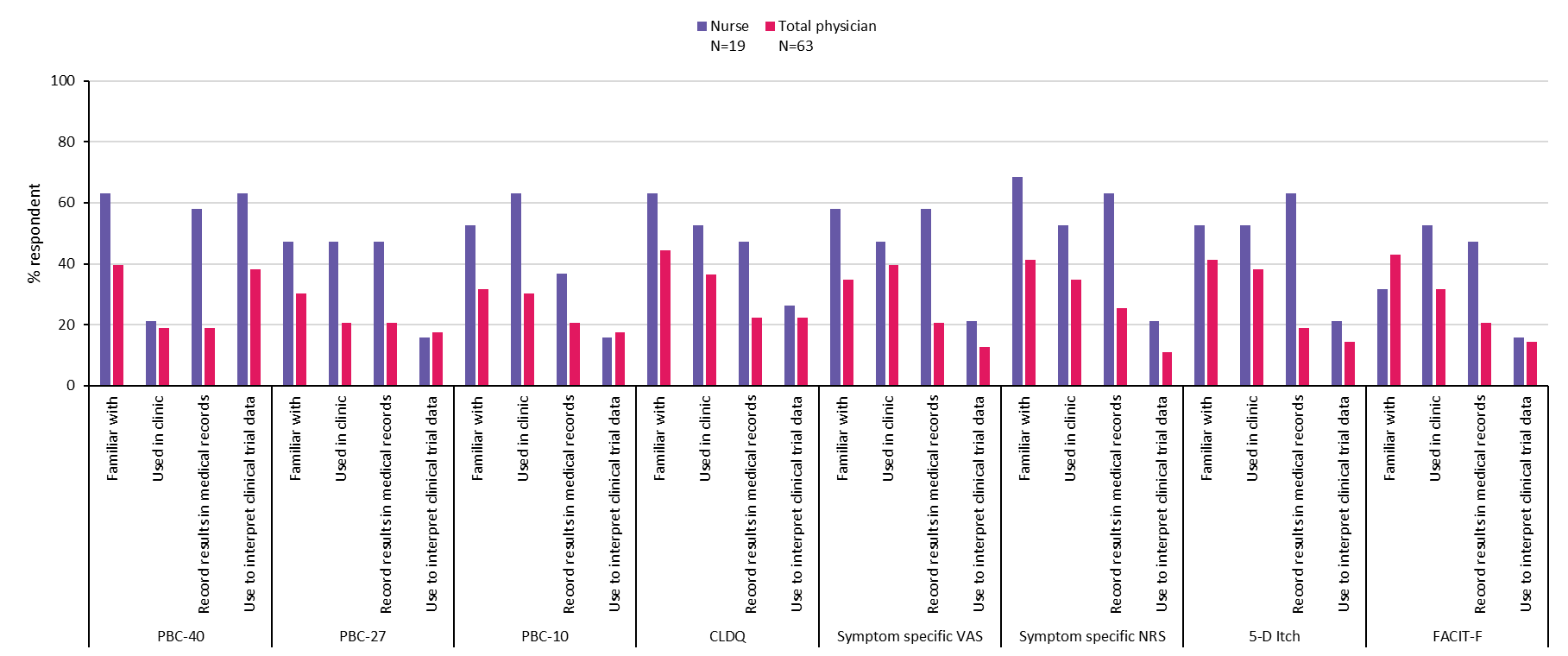


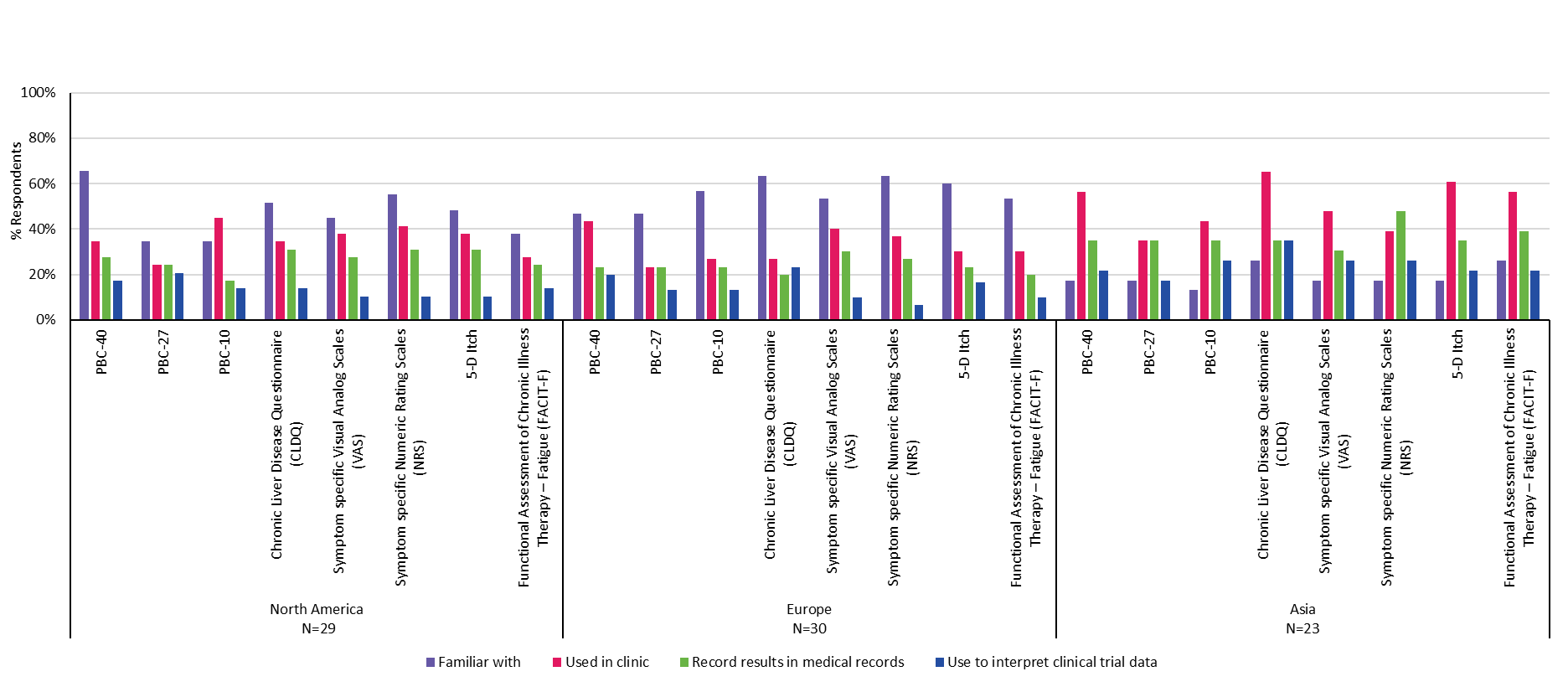


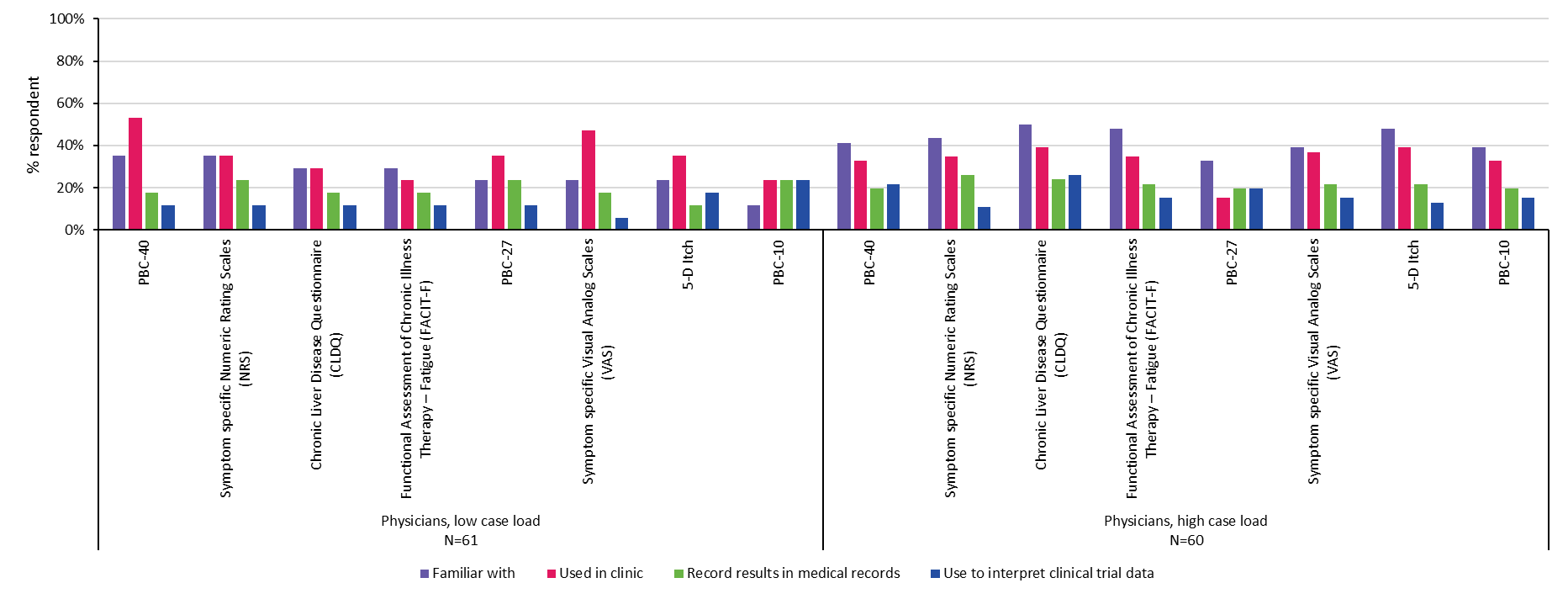


### Q23b. What are the reasons for not using a tool that you are familiar with in the clinic?


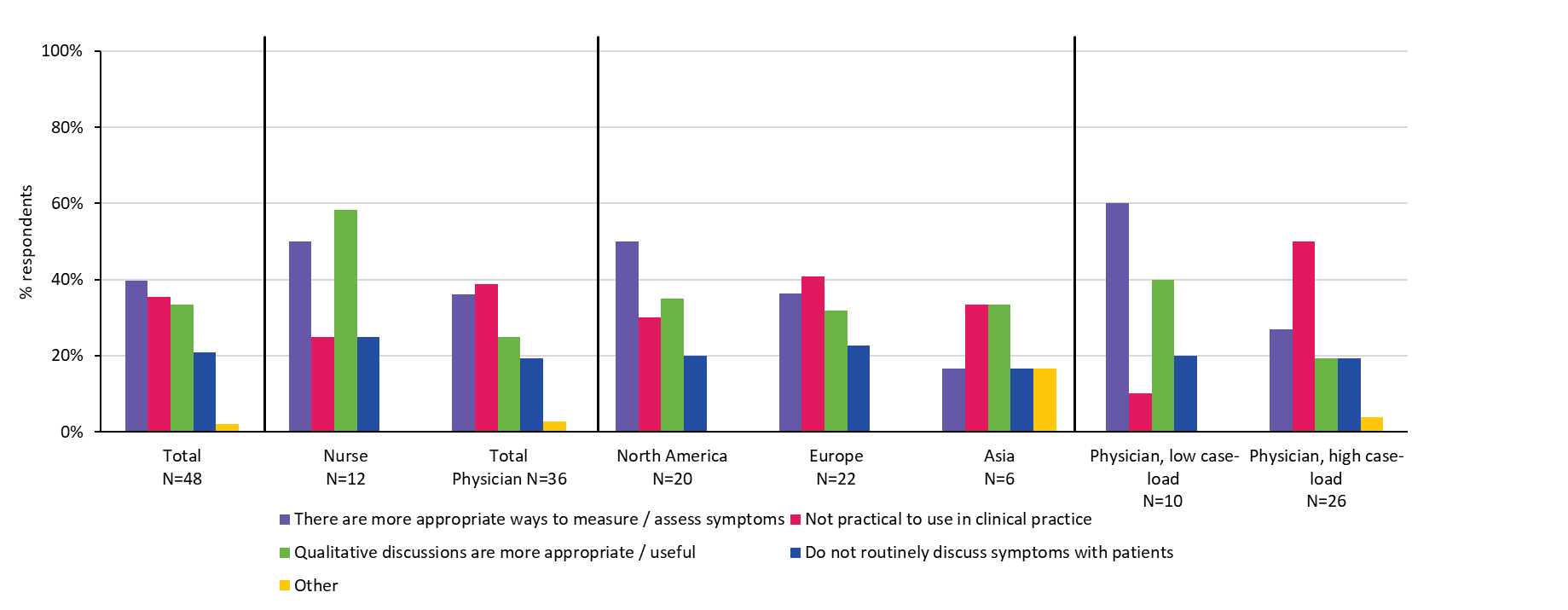


Other responses: more important to capture patient's own words (N=1).

### Q24. For the patients with PBC that you manage, how often do you discuss these specific symptoms with the patient?


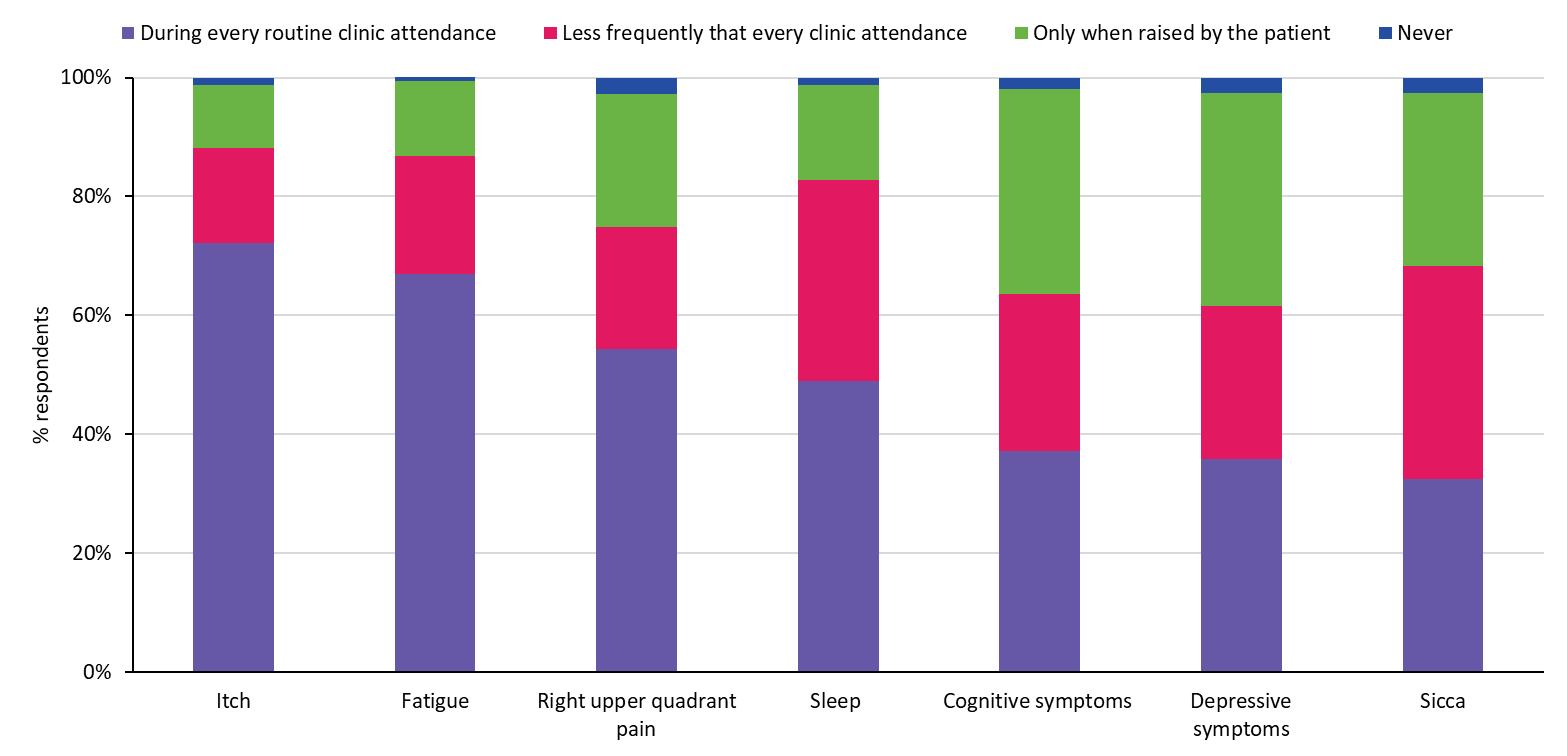


Other responses: diet (N=2); fertility, jaundice, weight change, swelling, neuropathy, urine color, nausea (N=1 each).


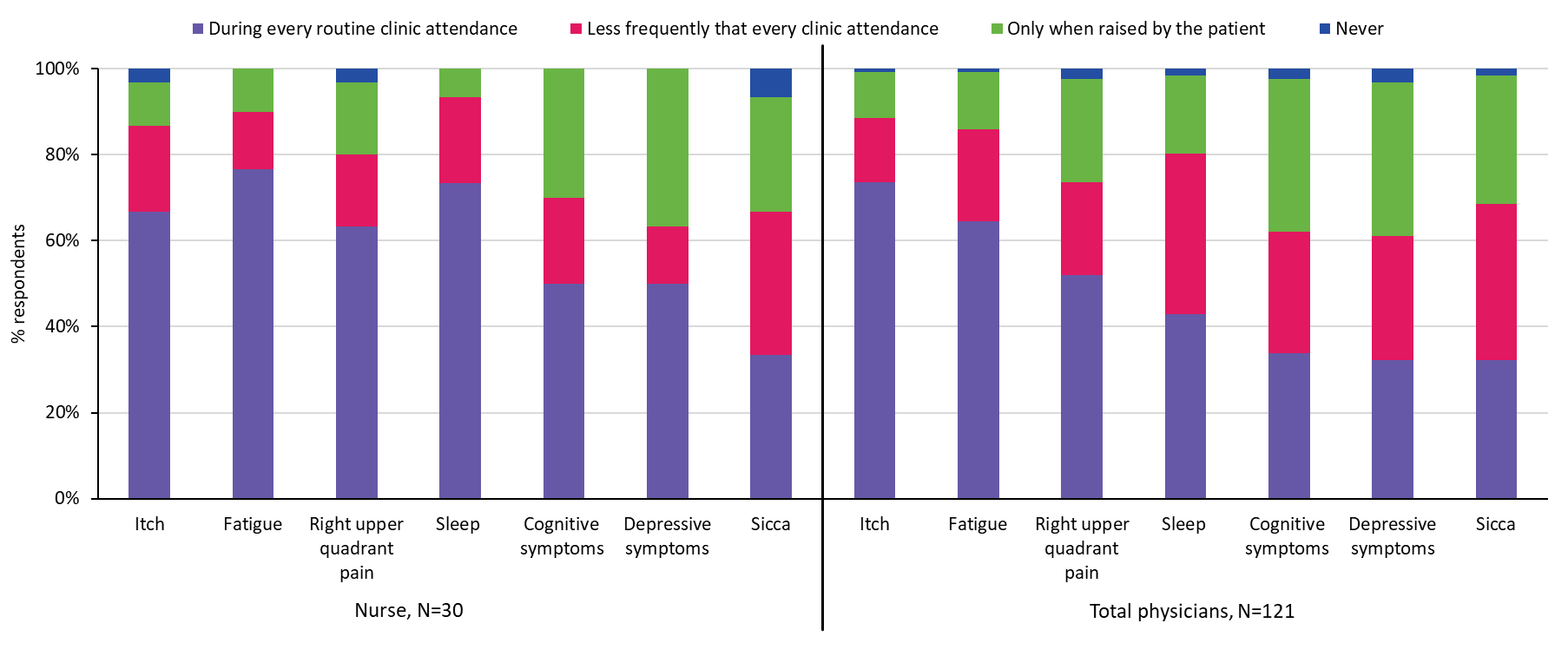


Other responses: diet (N=2); fertility, jaundice, weight change, swelling, neuropathy, urine color, nausea (N=1 each).


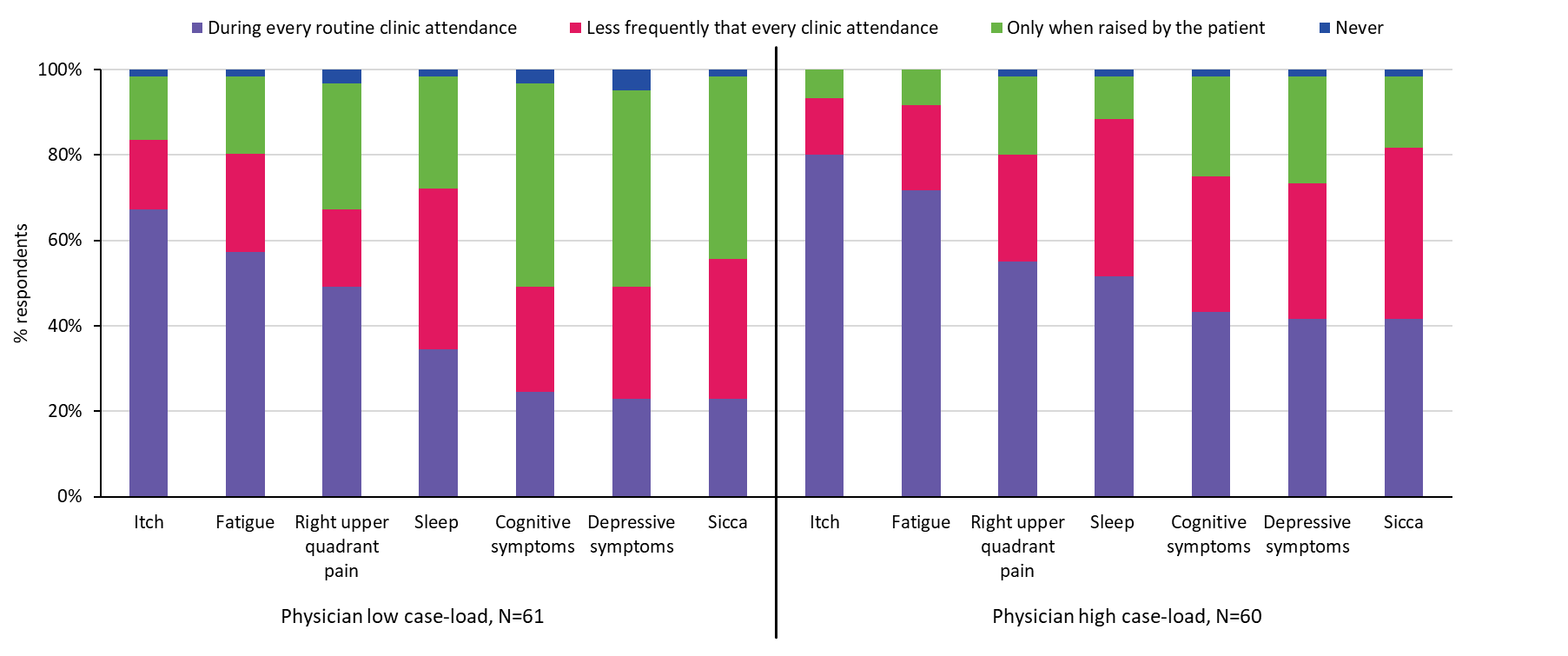


Other responses: diet (N=2); fertility, jaundice, weight change, swelling, neuropathy, urine color, nausea (N=1 each).


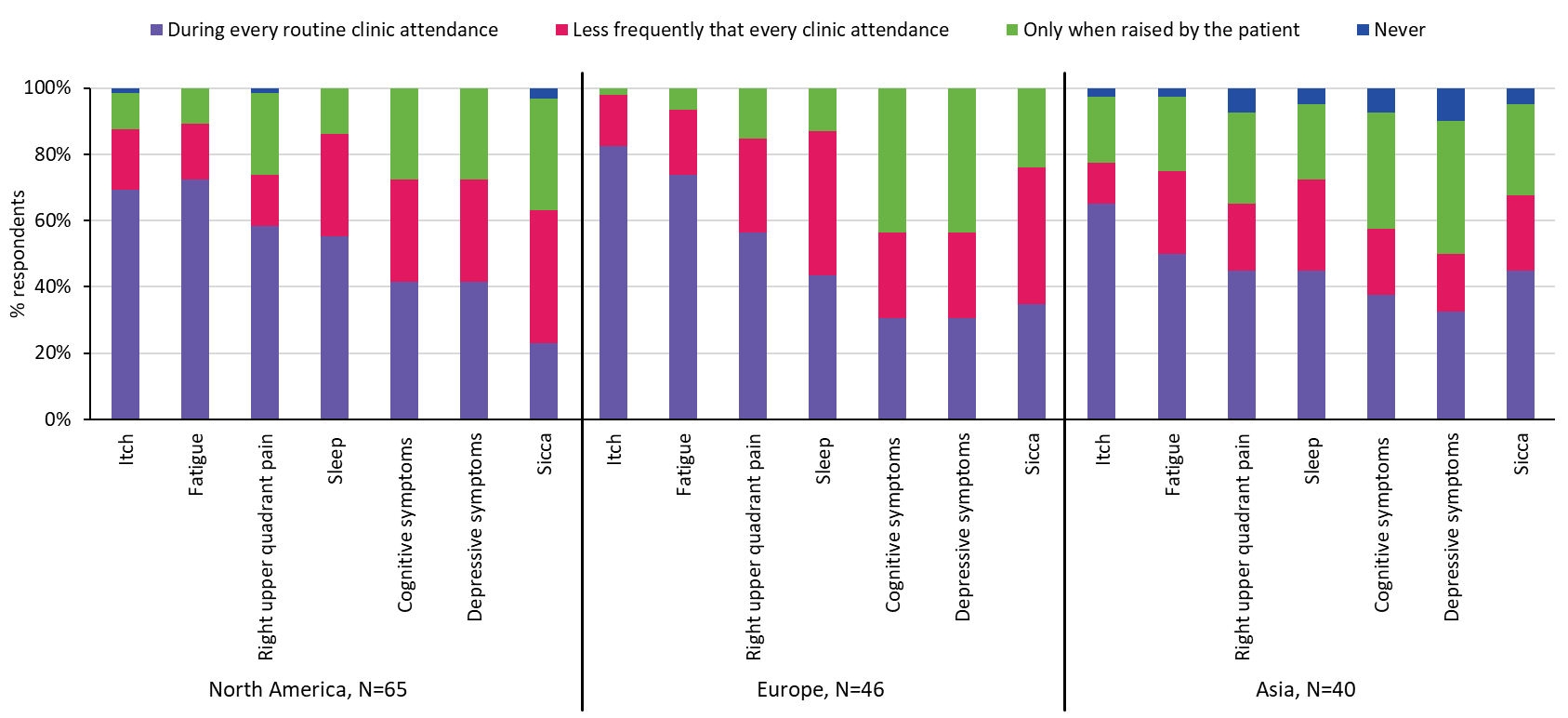


Other responses: diet (N=2); fertility, jaundice, weight change, swelling, neuropathy, urine color, nausea (N=1 each).

### Q25. When you identify a patient with symptoms, how do you prioritize which ones you should proactively manage?


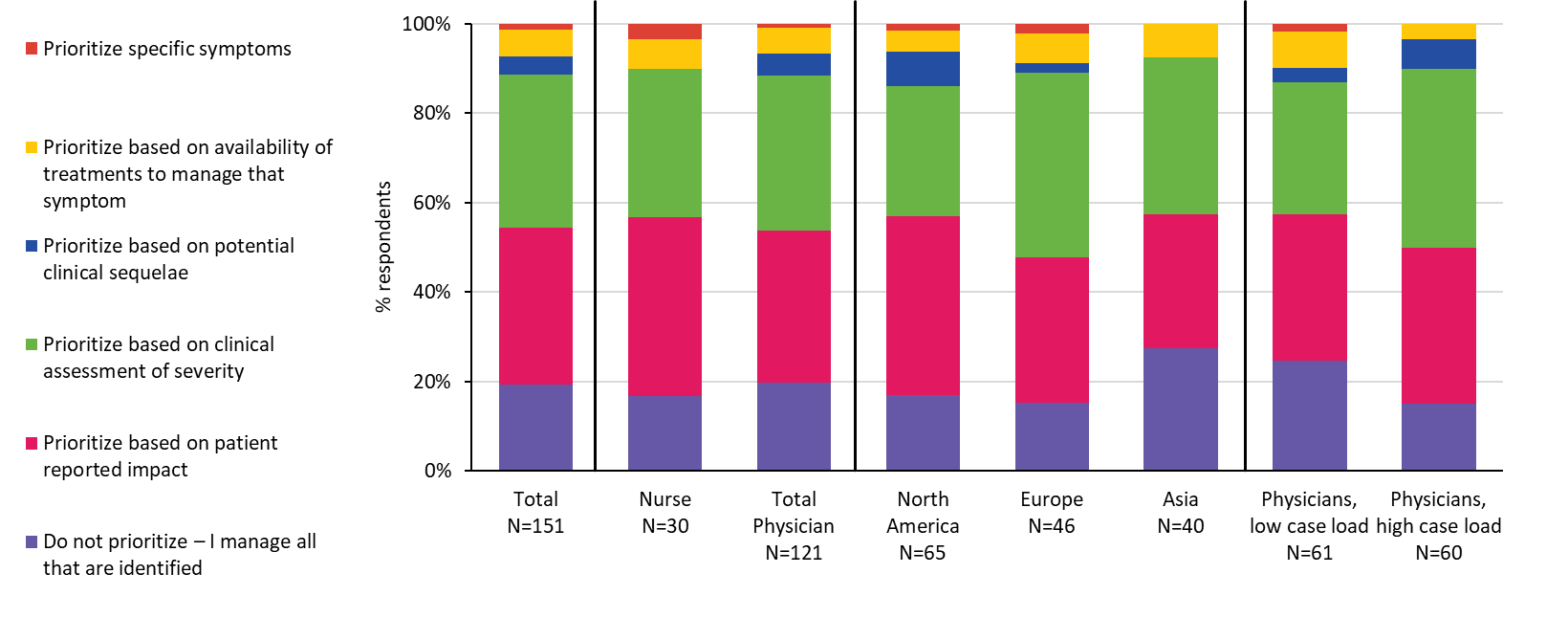


Itch, fatigue and jaundice were the specific symptoms prioritized (N=1 each).

### Q26. Does your approach to symptom management for a patient differ depending on the stage of PBC?


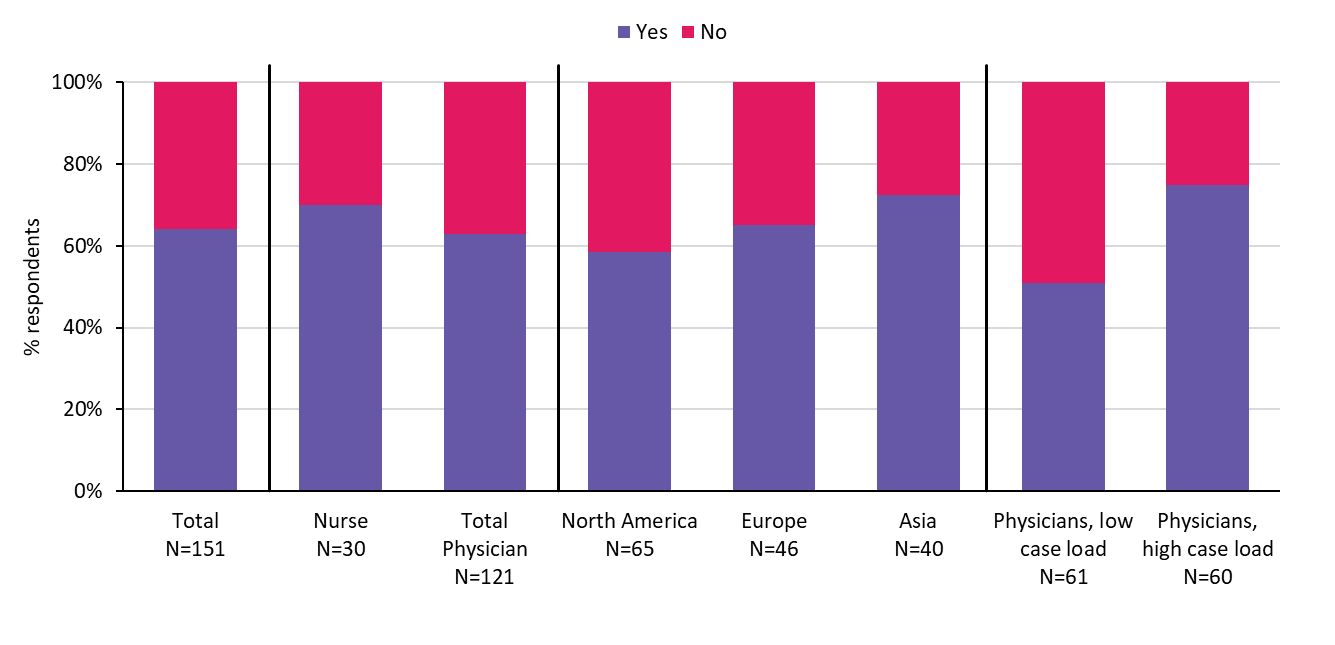


Itch, fatigue and jaundice were the specific symptoms prioritized (N=1 each).

### Q27. If a patient had a change in symptoms between scheduled consultations, which of the following options are available to them?


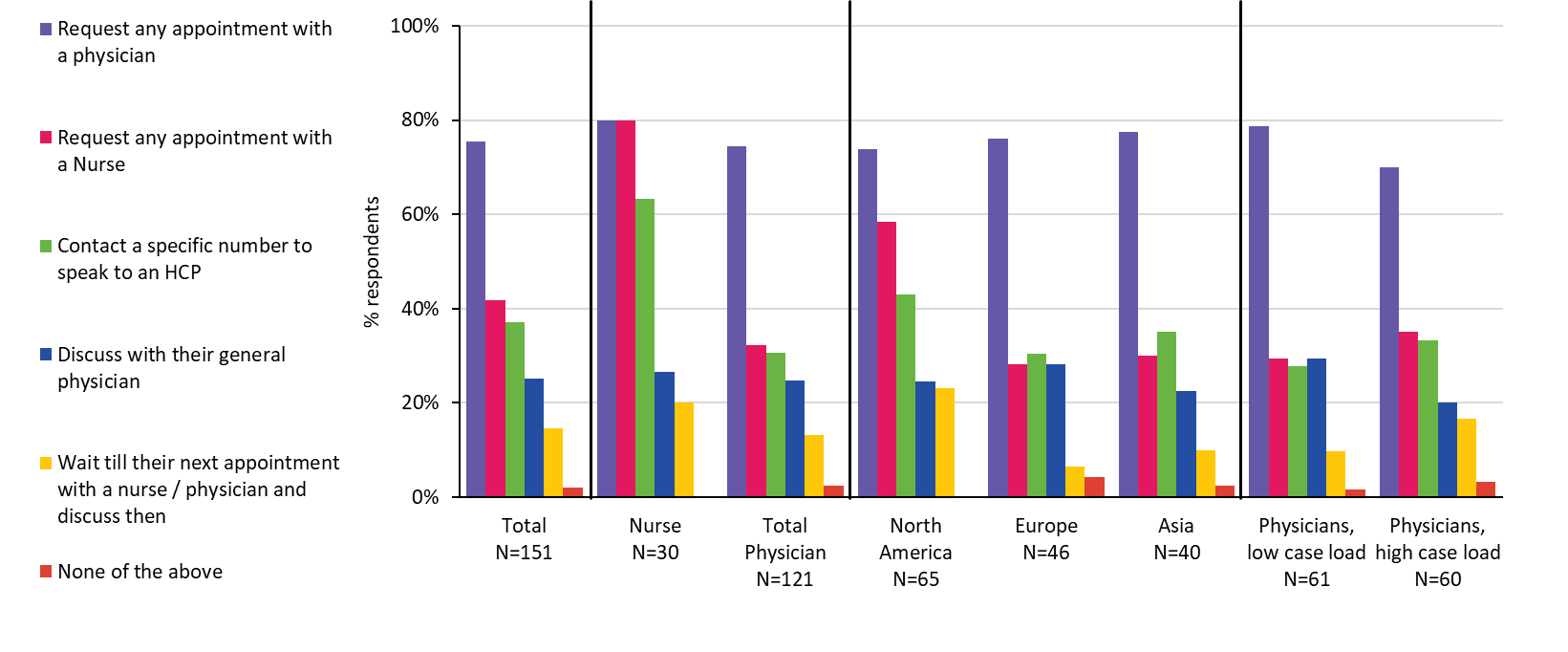


### Q28. Would quality improvement (QI) metrics that are linked to the management of PBC be useful in your practice?


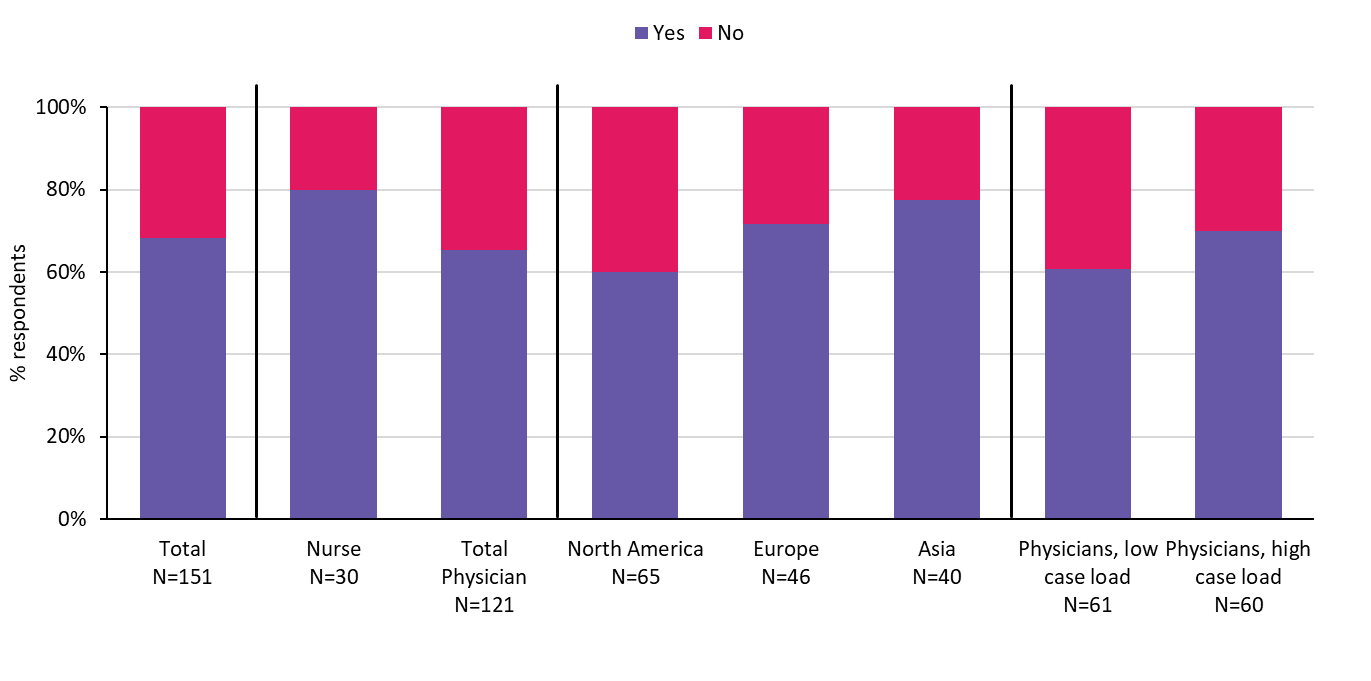


### Q28a. Would quality improvement (QI) metrics that are linked to the management of PBC be useful in your practice? If yes, please specify which aspects they should cover


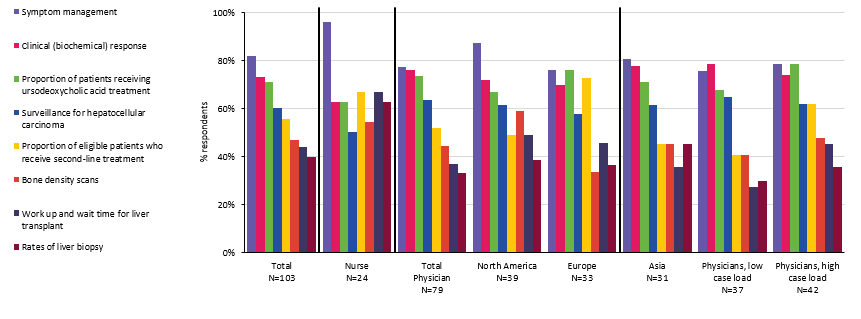


## Care and support providers

### Q29. Which wider care team members are available for your patients with PBC?


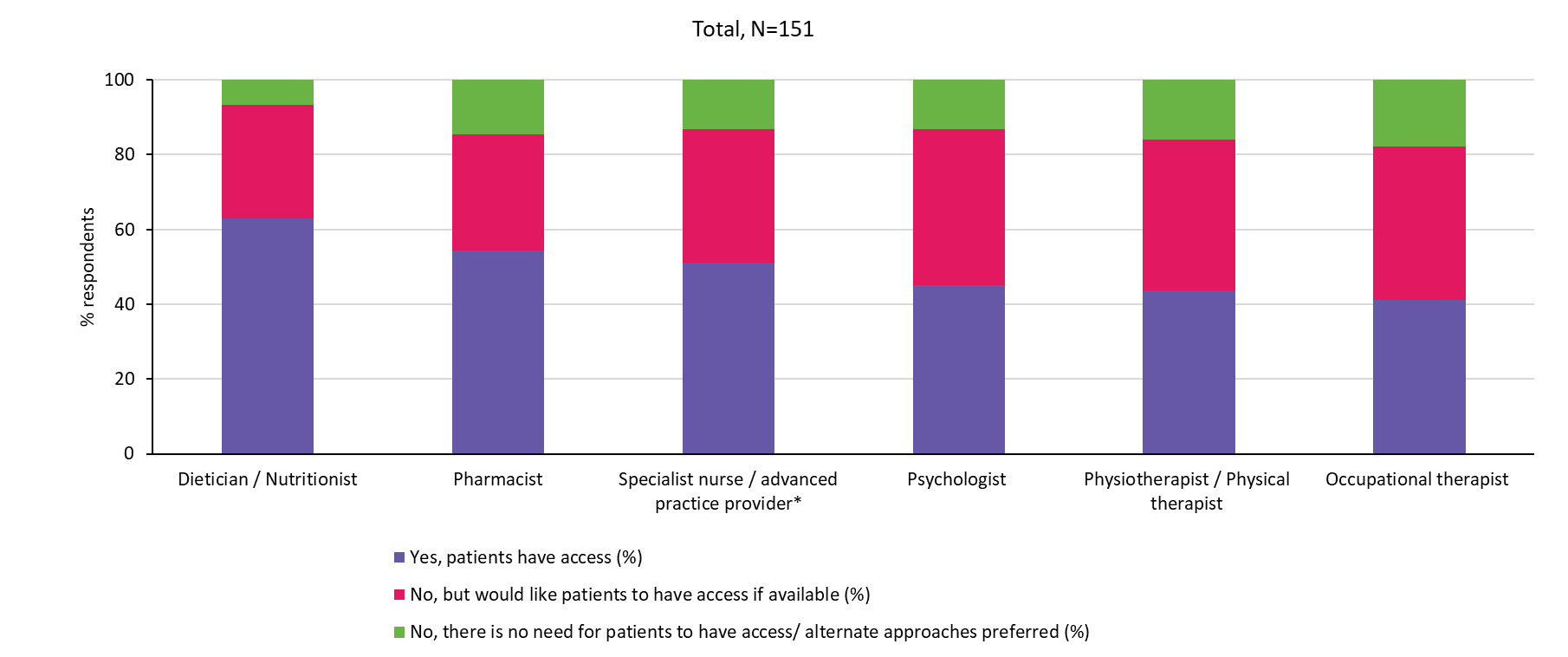


*Only physicians (N=121) were asked if their patients have access to specialist nurses.


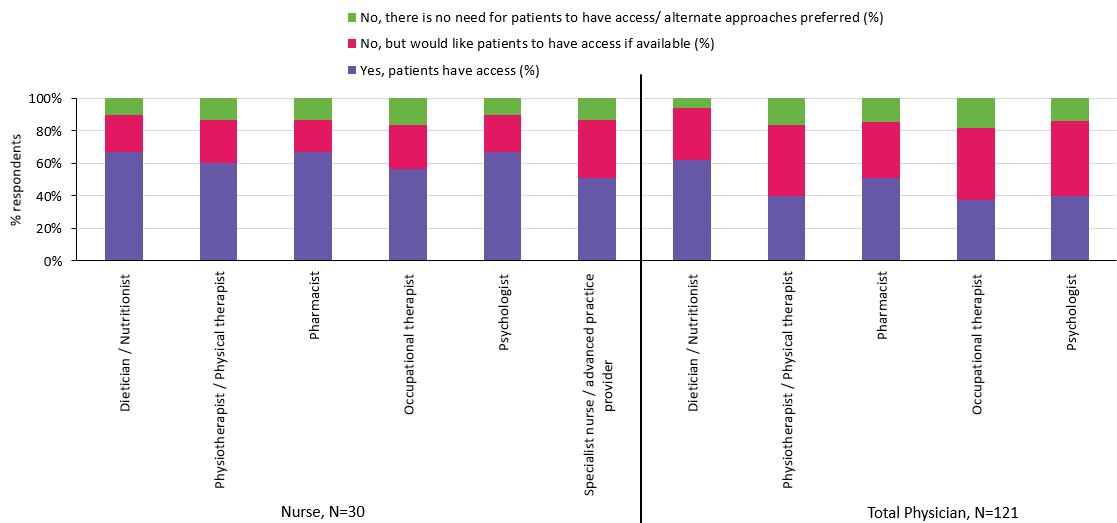


*Only physicians (N=121) were asked if their patients have access to specialist nurses.


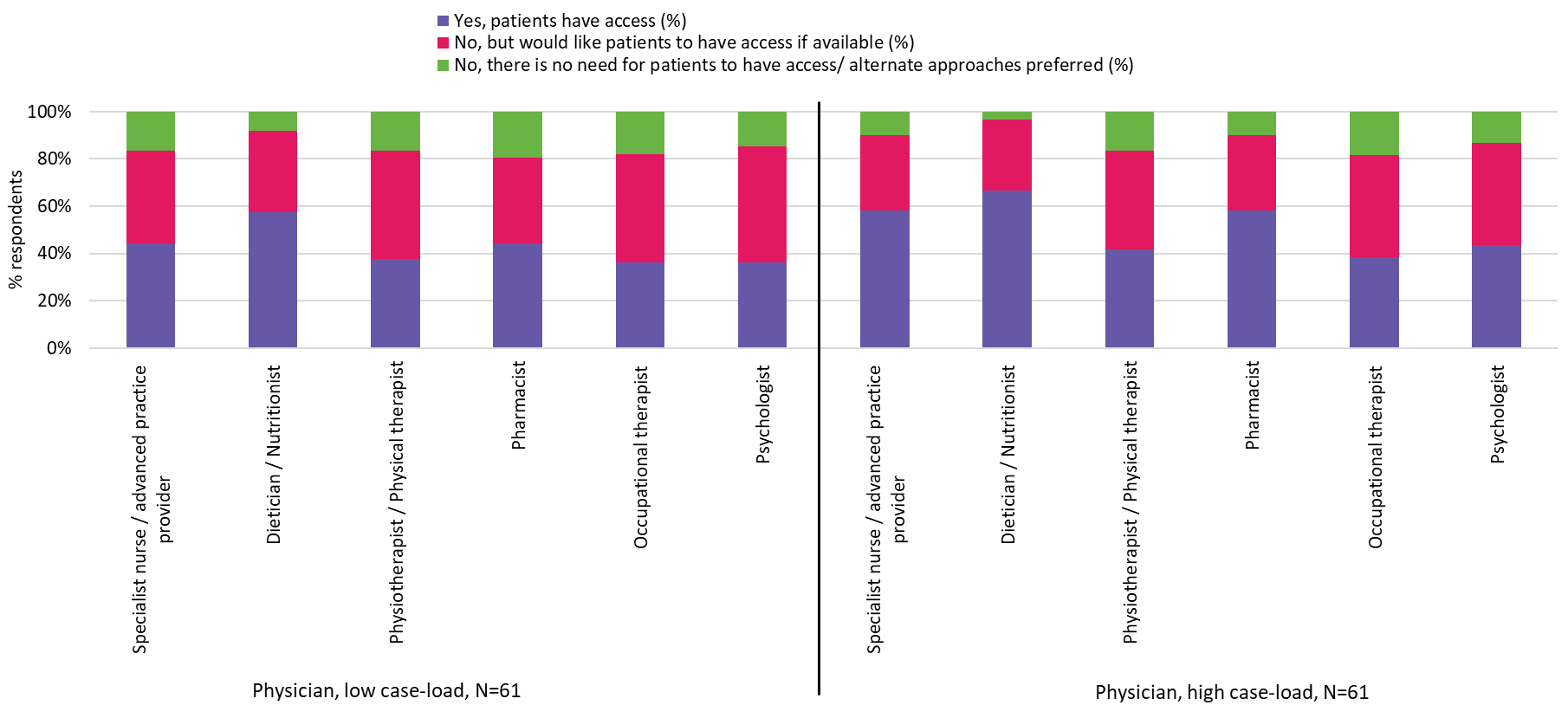


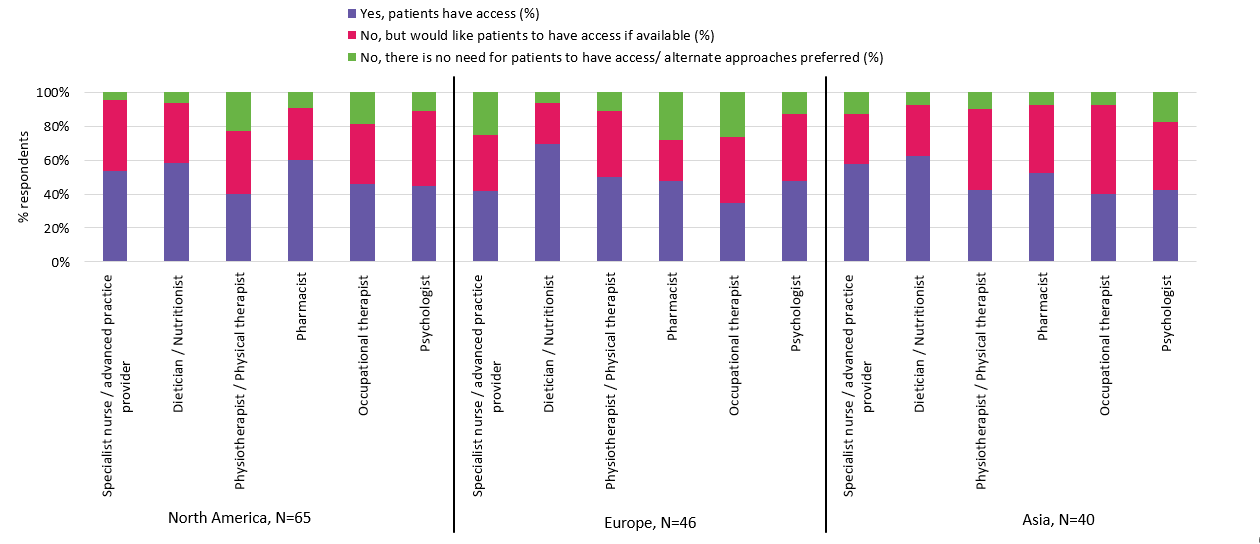


*Only physicians (N=45 North America, N=36 Europe and N=40 Asia) were asked if their patients have access to specialist nurses.

### Q30. Do you routinely suggest that patients should connect with Patient Organizations?


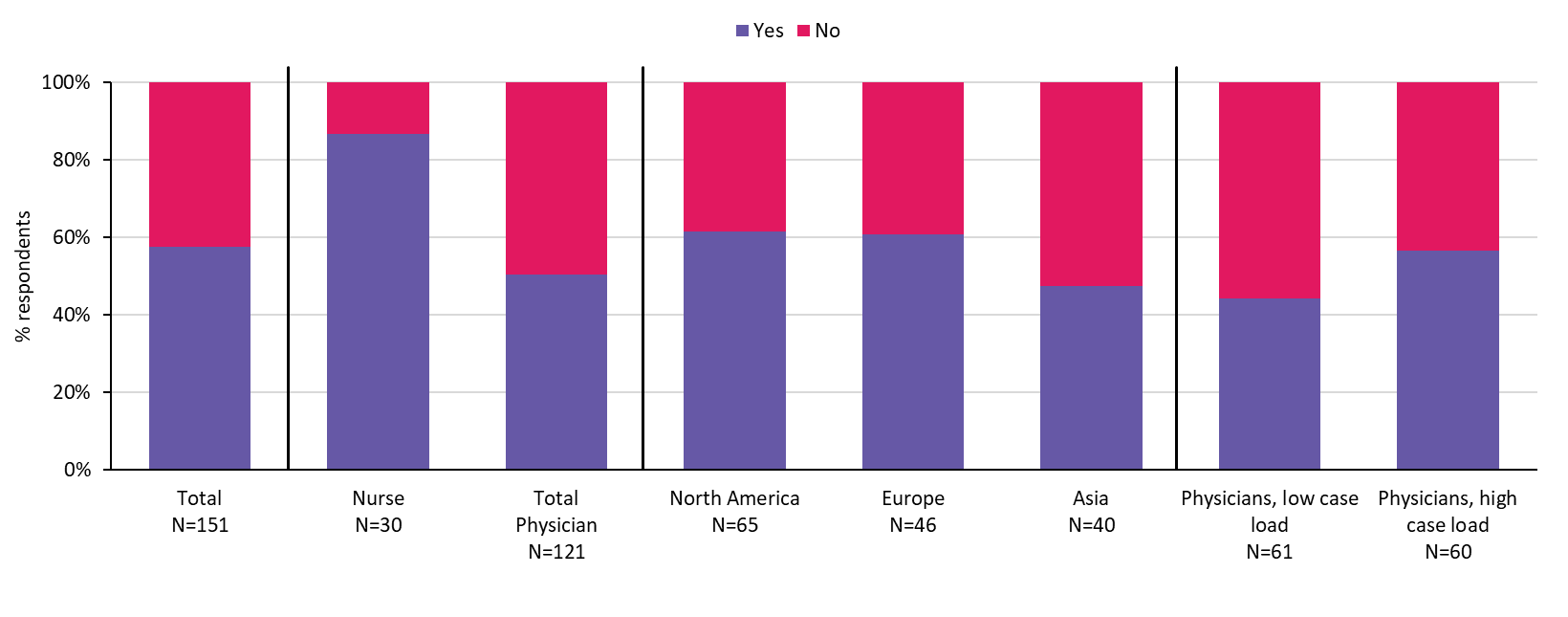


### Q30a. Do you routinely suggest that patients should connect with Patient Organizations? If so, how is this done?


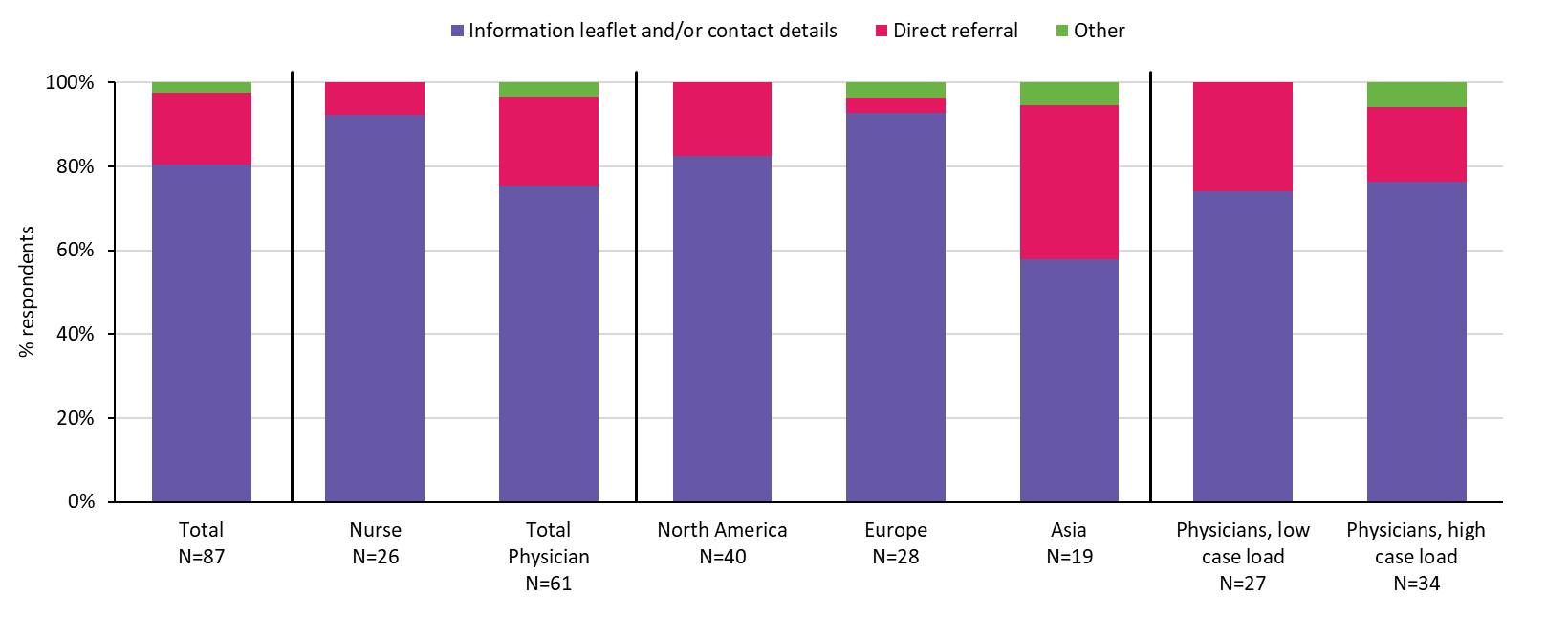


Other responses: internet / website (N=2).

### Q30b. Do you routinely suggest that patients should connect with Patient Organizations? If so, when is this done?


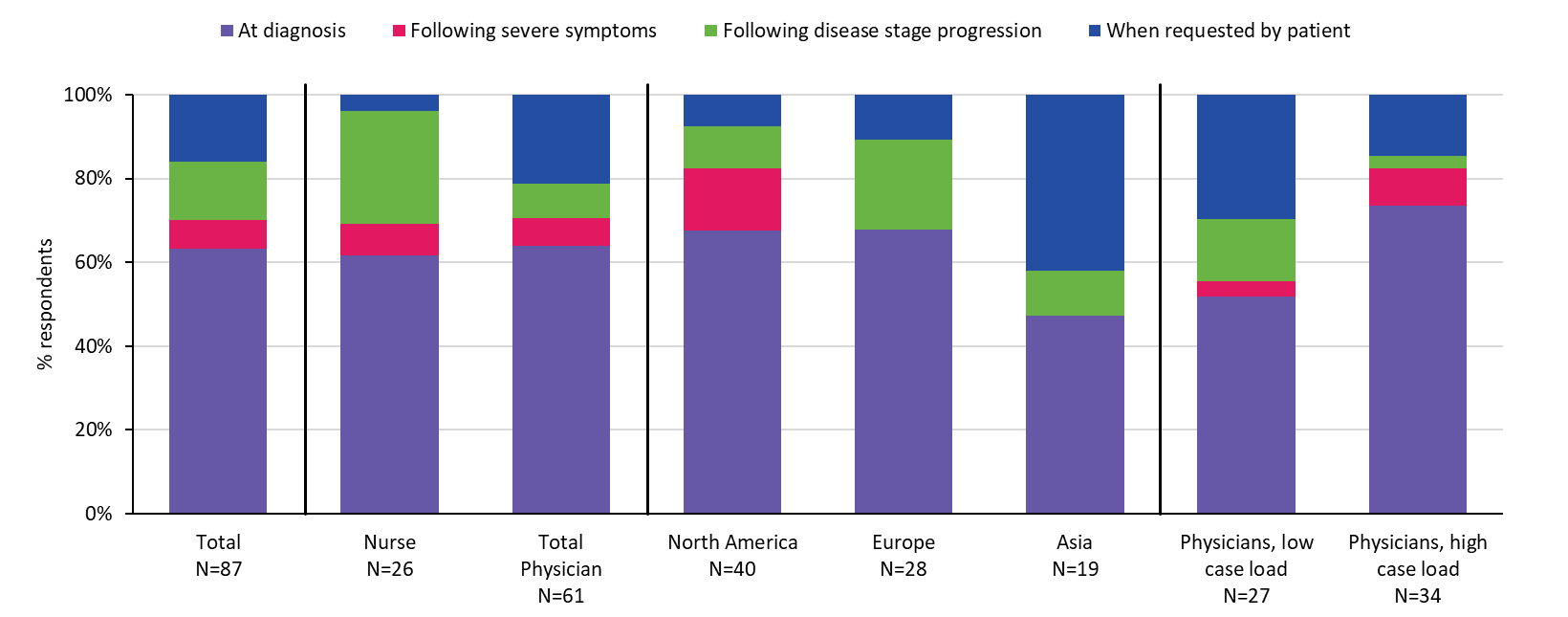


Other responses: internet / website (N=2).

### Q30c. Do you routinely suggest that patients should connect with Patient Organizations? If not, why not?


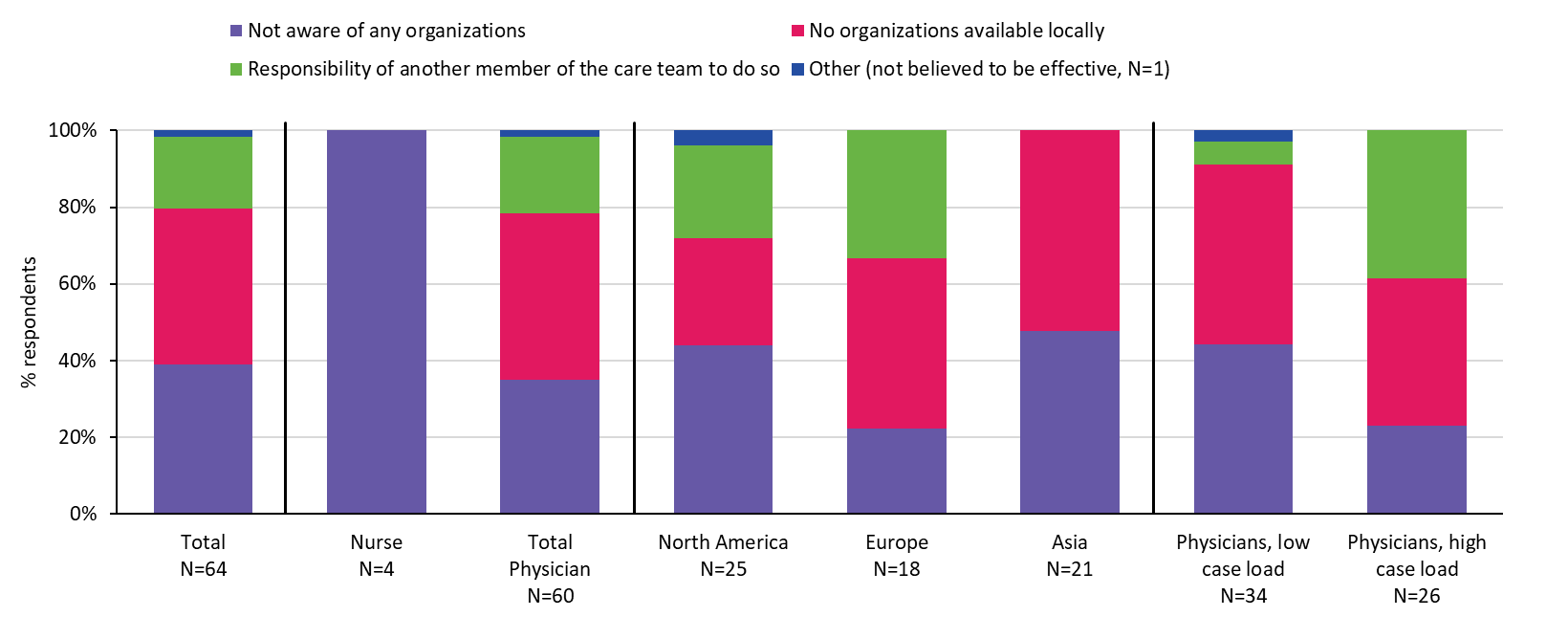


Other responses: internet / website (N=2).

### Q31. Do you ever refer a patient to a separate specialist for symptom management?


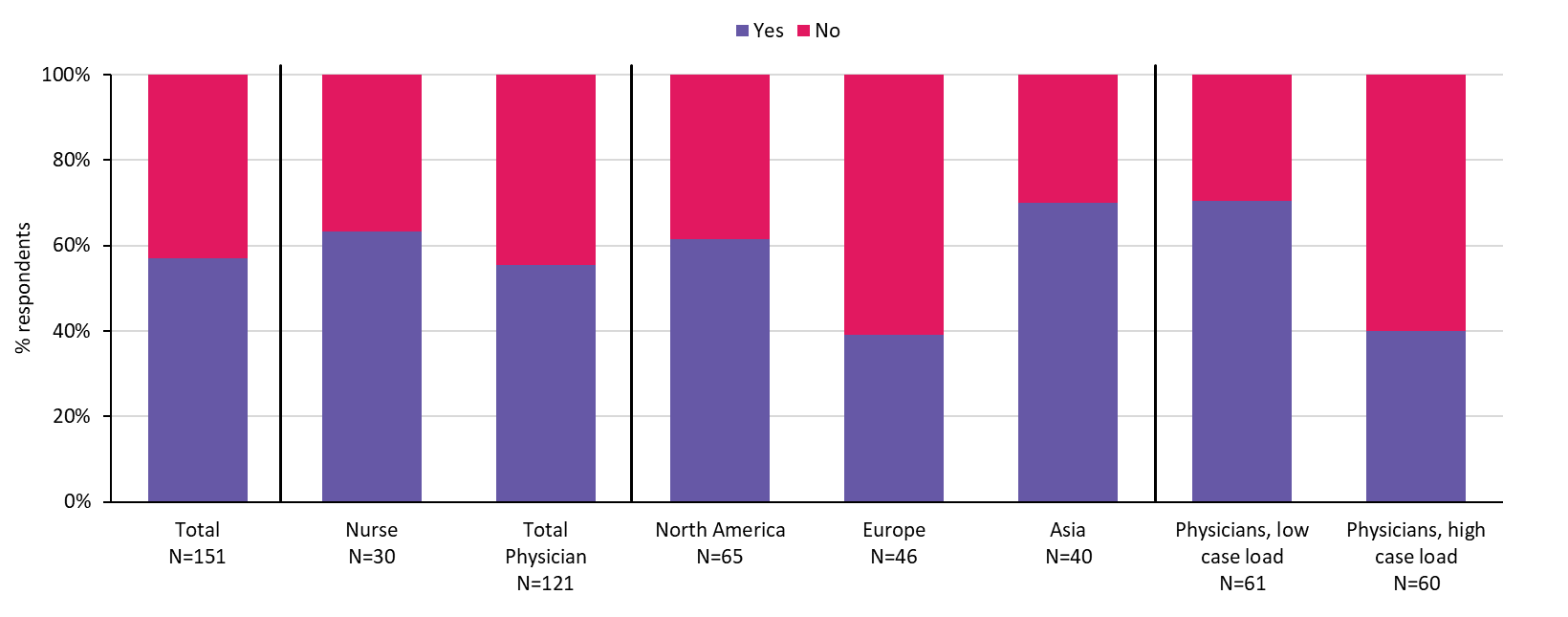


Other responses: internet / website (N=2).

### Q31a. Do you ever refer a patient to a separate specialist for symptom management? If so, what determines this?


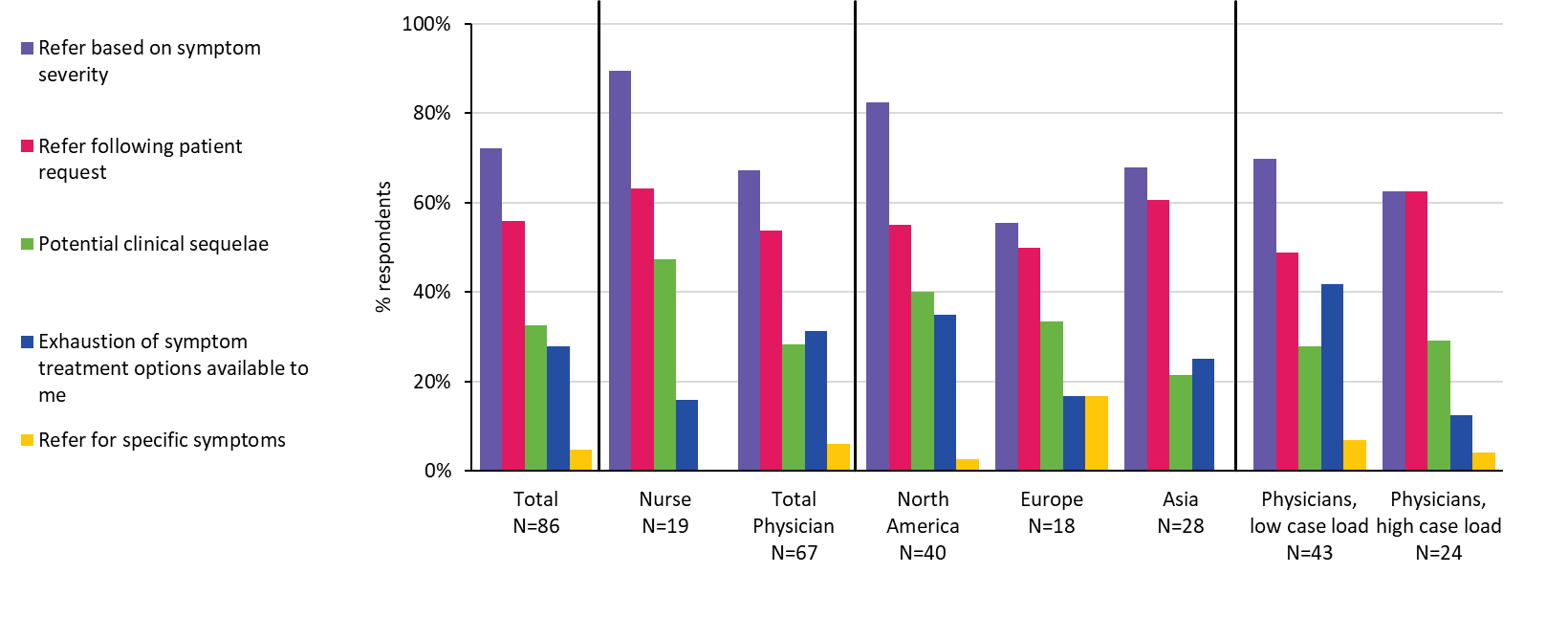


Specific symptoms: chronic pain (n=1); depression / mental health (n=2); fatigue (n=1) and dermatological symptoms (n=1).

### Q31b. Do you ever refer a patient to a separate specialist for symptom management? If yes, which are the symptoms where you would most commonly refer to a specialist?


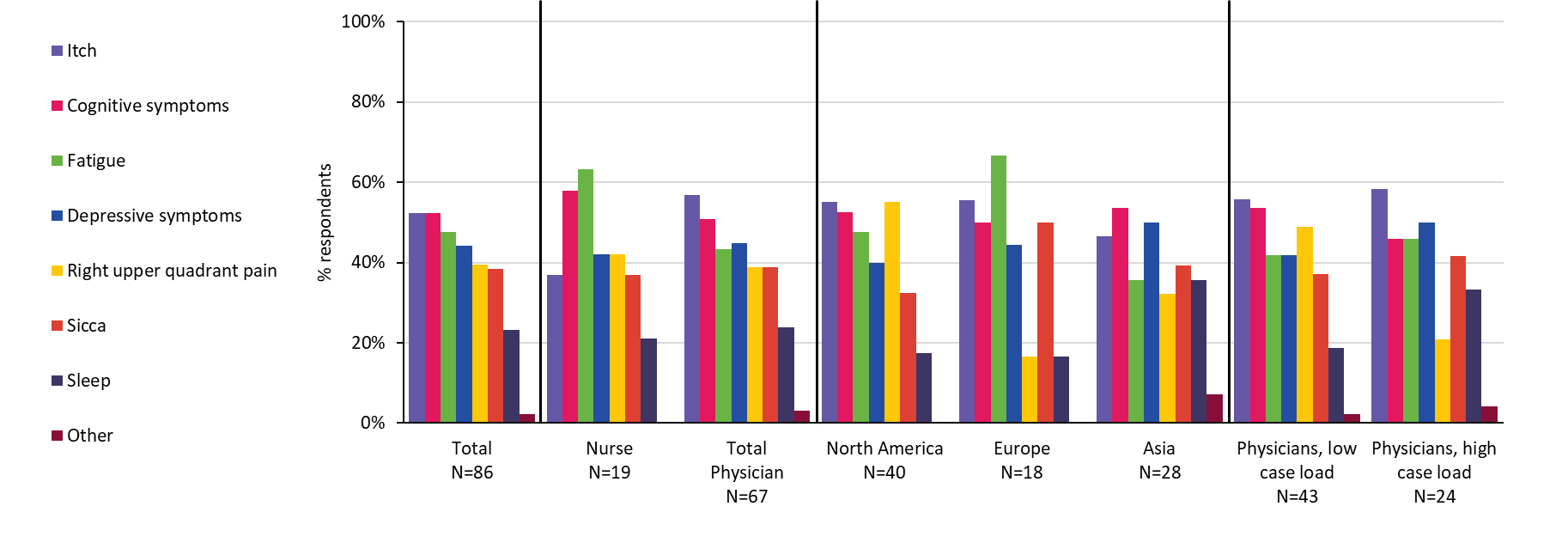


Specific symptoms: chronic pain (n=1); depression / mental health (n=2); fatigue (n=1) and dermatological symptoms (n=1).

### Q31c. Do you ever refer a patient to a separate specialist for symptom management? If no, why not?


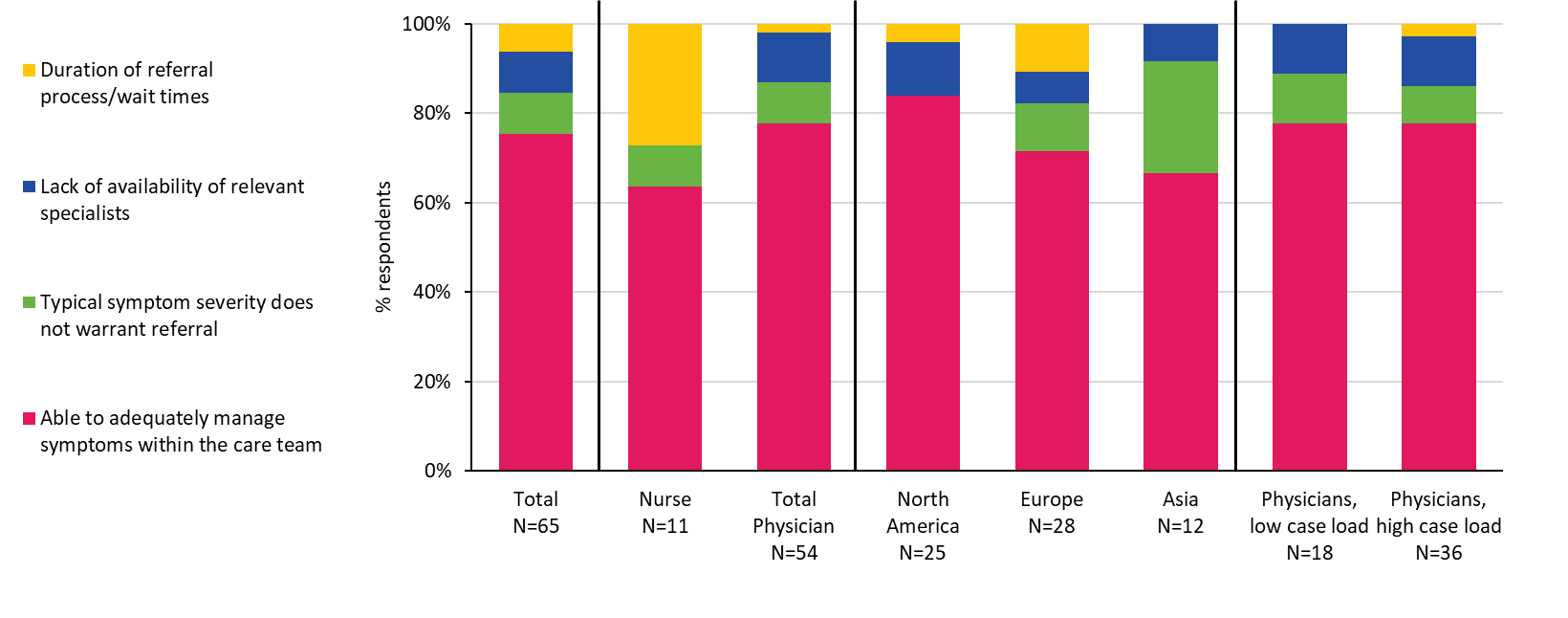


Specific symptoms: chronic pain (n=1); depression / mental health (n=2); fatigue (n=1) and dermatological symptoms (n=1).

### Q33. Who do you consider is responsible for managing extrahepatic complications of PBC (thyroid, bone density, oral health)?


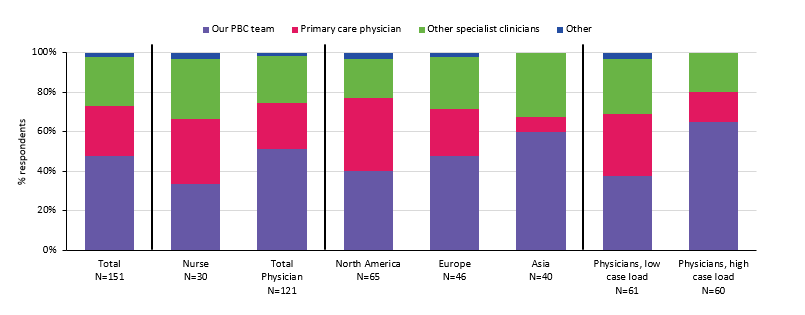


# Reference

1. Rattray J, Jones MC. Essential elements of questionnaire design and development.
*J Clin Nurs.* 2007;16(2):234-243.
